# A Proposal for the RNAome at the Dawn of the Last Universal Common Ancestor

**DOI:** 10.3390/genes15091195

**Published:** 2024-09-11

**Authors:** Miryam Palacios-Pérez, Marco V. José

**Affiliations:** 1 Instituto de Investigaciones Biomédicas, Universidad Nacional Autónoma de México, Ciudad de México 04510, Mexico; 2 Network of Researchers on the Chemical Emergence of Life (NoRCEL), Leeds LS7 3RB, UK

**Keywords:** RNA, RNY code, primeval genetic code, extended genetic codes 1 and 2, evolution of genetic code, FUCA, LUCA

## Abstract

From the most ancient RNAs, which followed an RNY pattern and folded into small hairpins, modern RNA molecules evolved by two different pathways, dubbed Extended Genetic Code 1 and 2, finally conforming to the current standard genetic code. Herein, we describe the evolutionary path of the RNAome based on these evolutionary routes. In general, all the RNA molecules analysed contain portions encoded by both genetic codes, but crucial features seem to be better recovered by Extended 2 triplets. In particular, the whole Peptidyl Transferase Centre, anti-Shine–Dalgarno motif, and a characteristic quadruplet of the RNA moiety of RNAse-P are clearly unveiled. Differences between bacteria and archaea are also detected; in most cases, the biological sequences are more stable than their controls. We then describe an evolutionary trajectory of the RNAome formation, based on two complementary evolutionary routes: one leading to the formation of essentials, while the other complemented the molecules, with the cooperative assembly of their constituents giving rise to modern RNAs.

## 1. Introduction

The evolution of translational machinery is one of the major evolutionary transitions rooted in the evolution of RNA [1]; it is estimated that ~4.36 ± 0.1 billion years ago, RNA evolution gave rise to the genetic code [2,3,4,5,6]. The primaeval genetic code (PGC) was composed of RNA molecules that followed an RNY pattern—where R represents purines, Y represents pyrimidines, while N symbolises any of the four nucleobases (A/G/C/U)—that were compliant with the R:Y parity rule for nucleotides (nt) [2,7]. The current standard genetic code (SGC) can be formed from the Eigen’s primaeval genetic code (PGC) via two different routes that conform two distinct collections of triplets with distinctive pattern each: “Extended Genetic Code 1, Ex1”, generated by frame-shift reading, forming the collection RNY+NYR+YRN; or “Extended Genetic Code 2, Ex2”, generated by transversions in first or third nucleotide (nt), forming the collection RNY+YNY+RNR [8,9].

We have previously delineated the proteome corresponding to each of the evolutionary stages, to wit the PGC [10] and both extended genetic codes (ExGCs) [11]. By starting to model the evolution of the RNA, we showed that RNY patterned-RNA molecules fold into small hairpins [12], as other works have strongly suggested, as the earliest molecular configuration of ancient RNA [13,14,15,16,17]. In addition, we were able to capture the anticodon of proto-tRNAs that can be charged by prebiotic amino acids (aa) in turn encoded by the PGC [12].

Herein, we outline a proposal for RNAome evolution based on the genetic code’s evolution, obtaining all the RNA molecules encoded by each of the ExGCs. In most cases, the biological sequences are more stable than their controls. Whether in bacteria or archaea, in sequence alignments, or in 2D or 3D structures, we discovered that the key features of RNA molecules are best recovered with triplets belonging to the Ex2 repertoire. The inability to salvage certain RNA molecules permits us to discard artefacts. On the other hand, the retrieval of tRNAs does not rely on whether the sequence is encoded by triplets of the Ex1 or Ex2 type; rather, the more ancient the encoding for a specific amino acid, the better the recovery of the corresponding tRNA.

## 2. Methods

We retrieved the RNAome from phylogenetically distant microorganisms, from which the triplets that did not belong to each of the extended genetic codes—Ex1 or Ex2—were discarded. The sequences were then classified according to their type, and each fragment was assembled in its original order in case the original gene had more than one fragment encoded by triplets of extended codes. To generate negative controls, the sequences were shuffled thrice. If more than one organism contained at least one RNA fragment encoded by RNY triplets, the fragments of each RNA type were arranged according to their original order in the gene. The RNAs were then aligned with each other to obtain a consensus sequence, and their corresponding logo sequences were also recovered. T was replaced by U in all sequences. Finally, we obtained the 2D and 3D structures of the RNAs encoded by extended triplets. The steps we took are all outlined below, and Supplementary File S1 contains a graphical flowchart.

### 2.1. Data Sources

From https://ftp.ncbi.nlm.nih.gov/genomes/refseq/ (accessed on 3 November 2023), we obtained all the RNAs (*RNA*.fna) encoded on each of the 26 organisms mentioned in Table 1 below.

### 2.2. Reconstruction of Sequences of Extended RNAomes

To reconstruct the original arrangement of the RNAome, all RNAs were assembled one after the other with an ad hoc program, just as they are reported in the file *RNA*.fna, to allow a posterior alignment. As a negative control, a random sequence using the assembled RNAome was generated, shuffling thrice the nucleotides to drop the biological sense of the sequence. From each RNAome and the corresponding shuffled control, we discarded the triplets that do not belong to each of the ExGCs (Ex1 and Ex2).

### 2.3. Classification and Assembly of RNAs

Using the standalone version of BLASTn [18], we were able to obtain the RNAs encoded by each ExGC by specifying the reconstructed RNAomes and their controls as the query and the file *RNA*.fna* of each organism as the database. As each ExGC is shorter than the SGC, we adjusted the parameters to allow as many outcomes as possible with the least restriction, setting 10 as the maximum E-value. The cut-offs for each organism were then determined by doing numerical comparisons between the E-values of each biological RNAome in Ex1 or Ex2 and their corresponding controls (those previously shuffled).

We classified all the retrieved fragments according to the RNA to which each one belongs. Additionally, the transfer RNAs were classified according to their cognate aa and the anticodon of each one, which we identified using the programs ‘tRNA finder’ [19] and ‘tRNA scan’ [20]. Please note that we annotated the anticodon in the tRNA and not the related codon.

### 2.4. MSAs and Consensuses of RNAs

To perform multiple sequence alignments (MSAs) we used the CHAOS-DIALIGN software version 2.2.2 [21], because it functions best with fragmentary sequences in local alignments. 

### 2.5. Consensuses of RNAs

From each MSA of our biological RNA sequences, encoded by ExGCs, we obtained the consensus sequence using the UGENE suite [22]. In certain instances, we aligned this consensus sequence with a consensus obtained from particular databases (like for SRP-RNA or tmRNA) or sequences that act as a patron or standard (like the Peptidyl Transferase Centre of the 23S rRNA). To eliminate each consensus sequence’s biological significance, we also shuffled it thrice, setting the outcomes as controls.

### 2.6. MFE of the Recovered Fragments 

The free energy of all the RNA sequences, including our biological sequences encoded by both ExGCs and their controls—the three shuffled ones—was predicted using the RNAfold web server of the ViennaRNA suite [23]. Within the web server, we selected the Andronescu model, avoiding isolated base pairs and leaving all other parameters the same, and then obtained the minimum free energy (MFE) values. In the case of the three shuffled controls, we averaged their MFE values and compared them with the corresponding biological sequence.

The secondary (2D) structures of biological sequences were visualised with the tool forna [24].

### 2.7. Sequence Logos

We generated a graphical depiction, of the MSAs of RNA molecules encoded by ExGCs triplets, known as sequence logos [25].

### 2.8. Representation in 3D of the Reconstructed Fragments

We reconstructed the possible three-dimensional (3D) structures of some RNA molecules encoded by the ExGCs with the aid of the web server ModeRNA [26]. Our RNA consensuses were used as targets, while the corresponding experimentally determined RNA molecule served as a template; first, to ‘build the model’ both the template sequence and the consensus sequence were aligned, then the alignment served to spatially allocate every nt of the target structure, based on the spatial distribution in the template; in some cases, the structures were cleaned, and its sequence requested before building the model, following the ModeRNA protocol.

The predicted 3D structures were compared then with the structure of the corresponding current modern molecule, obtained from the repository RCSB-PDB [27,28]. Structural comparisons were performed with the help of the size-independent nt-to-nt alignment web server program named RNA-align [29] from the Zhang Lab suite. This program’s metric, dubbed “TMscore_RNA_”, is distance-based with normalised values (0,1], and a match ≥0.45 indicates that both structures are at least in the same Rfam family. Since molecules encoded by ancient genetic codes may be shorter than molecules encoded by the SGC, the TM*_score_* is preferred over the classical RMSD score precisely because the latter considers the length of both sequences, and the results could be overestimated or underestimated.

The alignments between the target and the template were visualised in UGENE [22], and we extracted also the conservation profiles. All the 3D structures were visualised with the Chimera program [30].

## 3. Results and Analyses

All the chosen 26 organisms have a wide variety of lifestyles, anbd they are phylogenetically distant. We did not consider organisms other than bacteria and archaea, because we subscribe to the most recent updating of the Tree of Life (ToL) that classifies eukaryotes among the latter, and Lokiarchaeota as the most plausible transition [31,32,33,34,35,36]. 

De novo RNA molecule modelling presents a number of difficulties that grow with molecule length due to the fact that RNA folding is dependent on a wide range of factors [37]. Given the fragments encoded by both types of ExGCs cover sizeable chunks of each RNA, and MSAs can be carried out, we can safely expect that the consensuses will eventually fold in a manner akin to that of contemporary structures, so we could use them as templates.

In the MSA step, we used different online software programs, such as ClustalW (https://www.ebi.ac.uk/jdispatcher/msa/clustalo, accessed on 1 November 2023), MUSCLE (https://www.ebi.ac.uk/jdispatcher/msa/muscle?stype=protein, accessed on 1 November 2023), and T-coffee (https://www.ebi.ac.uk/jdispatcher/msa/tcoffee?stype=protein, 1 November 2023). We even used the specialised online aligner of the SILVA project, which functions very well with whole ribosomal sequences, although its reconstructions were not as reliable as those produced with the unpenalised CHAOS-DIALIGN software version 2.2.2 and DIALIGN-Tx software version 2.1. The local reliability of alignments was evaluated with the webservice Transitive Consistency Score (TCS) provided by T-coffee [38] and the Gobics program was particularly useful in detecting local homologies in sequences with low overall similarity, probably because it preserves the position of each fragment. It is also evident from a visual inspection of the results that, in contrast to the MUSCLE software which was supposedly better but did not maintain order and position, the DIALIGN program performs exceptionally well making the consensus sequences more reliable. This is especially true when dealing with short fragments from some organisms, as opposed to nearly whole sequences in others.

An RNA structure’s MFE value represents its stability; the more stable a structure is, the further away from zero its MFE value will be. Therefore, it is preferable for a biological sequence’s MFE to be lower (more negative) than that of the corresponding randomised sequence, which will serve then as a negative control; in our case, we shuffled the biological sequence three times and obtained the average of their MFE values. Most of the biological RNA molecules (Bio. Seq.) have lower MFE values than their controls, indicated in Supplemental Material S2 as “AVG_3shffs” in pale-yellow cells (Supplemental Material S2). Of course, there are exceptions that support the assertion that our methodology does not generate artefacts. The background of the cell is coloured red poppy if the average of the shuffles has a lower MFE value than the Bio. Seq. In rare instances where the averaged MFE values closely match the MFE values of Bio. Seq. (less than one unit), we show the control to be slightly higher or lower in pale pink or pale turquoise, respectively.

We reconstructed the 3D structure of some RNA molecules encoded by both types of ExGC, using as templates the corresponding structure experimentally obtained by X-ray crystallography. Then, we compared the crystallographic structure (in purple) with the structures encoded by Ex1 triplets coloured in salmon and encoded by Ex2 coloured in sky blue. The TM*_score_* relative to the experimental molecule is given.

Overall, this work includes MSAs reported as logos, 2D structural predictions of the consensuses, and 3D structural templated-based predictions using crystallographic-determined structures. From these, we derived conservation profiles and alignments between the consensus sequence and the structure sequence in the PDB database. All the figures in the main text can be found in the Supplemental Material S3.

In general, we found that all the RNA molecules analysed contain portions encoded by both ExGCs, but the crucial features are better recovered by Ex2 triplets. In other cases, differences lie between bacteria and archaea.

A take-home message is included at the beginning of each subsequent subsection to provide a quick overview of the content. 

### 3.1. 5S rRNA

5S rRNA, an integrating molecule for ribosome functionality, exhibits variations in conservation and structure, depending on whether it is encoded by Ex1 or Ex2 triplets. Additionally, the structures encoded by Ex2 triplets are slightly more similar to modern ones than those encoded by Ex1 triplets, and with archaeal sequences showing a greater resemblance to their modern counterparts compared to bacterial ones

5S rRNA is a small RNA molecule approximately 120 nt long that consists of three domains. It is positioned in the central part of the ribosome, amalgamating all the components necessary for translation. It acts as a structural and informational transducer that coordinates all the events catalysed by the ribosome, meaning any ribosome without 5SrRNA becomes non-functional [39,40,41,42]. 

The averages of shuffled 5S rRNAs show lower MFE values than the biological sequences for triplets pertaining to both ExGCs in nearly all cases (shadowed in poppy), with one exception where, as expected, the MFE value of the bacterial 5S rRNA encoded by Ex2 triplets is lower than the average of its randomised sequence (Supplemental Material S2).

**Figure 1:**
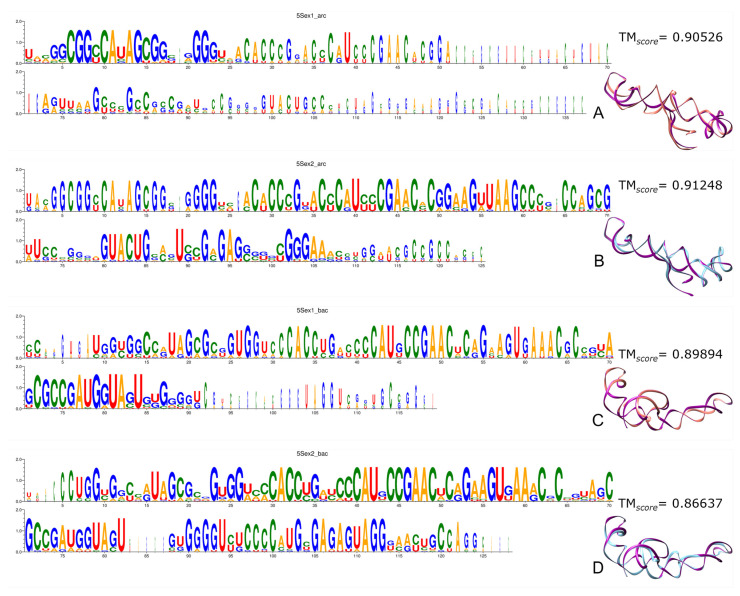
Logo sequences and 3D structures of ExGCs-encoded 5S rRNA. In (**A**) the Ex1-encoded 5S rRNAs from arc., Ex1-encoded portion (salmon) is superimposed with current 5S rRNA (1ffk purple, TM = 0.90526); in (**B**) the Ex2-encoded 5S rRNA from arc, Ex2-encoded portion (sky blue) is superimposed with current 5S rRNA (1ffk purple, TM = 0.91248); in (**C**) the Ex1-encoded 5S rRNA from bac., Ex1-encoded portion (salmon) is superimposed with current 5S rRNA (5gaf purple, TM = 0.89894); in (**D**) the Ex2-encoded 5S rRNA from bac., Ex2-encoded portion (sky blue) is superimposed with current 5S rRNA (5gaf purple, TM = 0.86637).

This molecule 5S rRNA, which brings together all the translation components to ensure the process is carried out in a coordinated manner, shows good sequence coverage in this work. However, while MSAs suggest that bacterial recover more readily, the structural reconstructions reveal that archaeal predictions—whether encoded by Ex1 or Ex2 triplets—are marginally more similar to their corresponding experimental molecules than those of bacteria (Figure 1 and Supplementary Material S3).

In the multiple analyses of the archaeal 5S rRNA, including comparisons with the structure 1ffk found in the PDB, we can observe that only the first third of the molecule encoded by Ex1 triplets is conserved (Figure 1A), while the more conserved rRNA encoded by Ex2 triplets also has a less conserved final region (Figure 1B).

On the other side, as we can visualise in the multiple analyses of bacterial 5S rRNA or in the comparisons with the structure 5gaf found in the PDB, Ex1 triplets encode most of the sequence well, although the end region is also poorly conserved (Figure 1C). Meanwhile, Ex2 triplets retrieve the molecule more effectively, with conservation well distributed along the length (Figure 1D). It should be remembered that this later molecule was the only one with expected MFE values.

The reason for the irregular behaviour of the molecule in our sample could be the previously reported high diversity of 5S rRNA [43].

### 3.2. 16S rRNA

16S rRNA, crucial for the translation process and widely used as a molecular marker in phylogenetic studies, shows significantly better conservation of the anti-SD sequence when encoded by Ex2 triplets in both archaea and bacteria compared to Ex1 triplets.

16S rRNA consists of little more than 1500 nt in length and is folded into three domains. It belongs to the small ribosomal subunit (30S), which interacts with all proteins to some extent [44,45,46], and is essential in the translation process. 16S rRNA contains a sequence, at the last 3′-end, that forms base-pairs with the Shine–Dalgarno (SD) messenger-RNA (mRNA) sequences of prokaryotic organisms. The pairing of SD with the anti-SD motif, which is minimally the sequence CCUCC (GAUCACCUCC in “EcoK12” and AUCACCUCCU in “Mejan”), correctly positions the translation initiator codon (AUG) [47,48,49]. Additionally, bacterial 16S rRNA contains two adenines—A1492 and A1493—that form hydrogen bonds on the minor groove of the complex codon-anticodon complex, fastening them, even with tRNA wobbling, avoiding the near-cognate tRNAs, and thus ensuring rapid translocation in the decoding centre [50,51,52].

**Figure 2:**
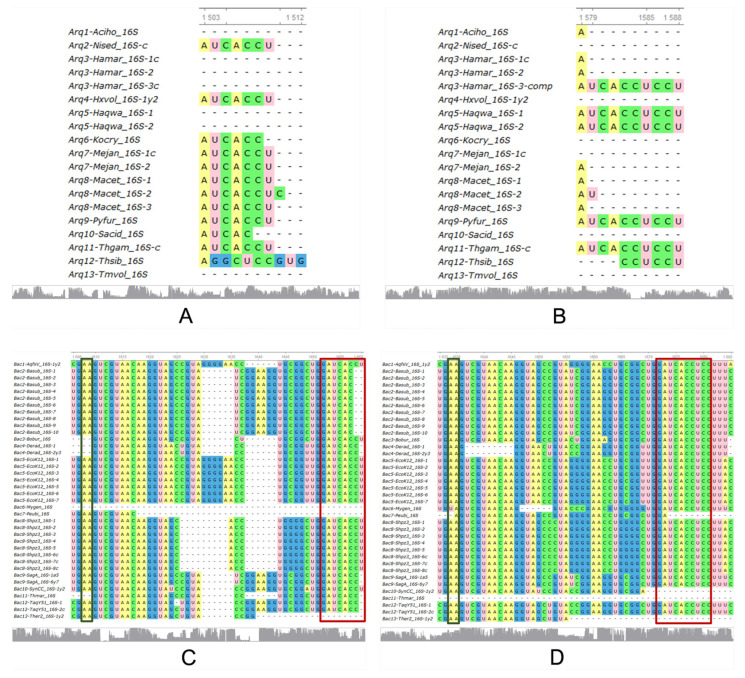
Subalignments (subalns.) extracted from the MSA of the ExGCs-encoded portion of 16S rRNAs anti-SD sequence context and the conservation profiles of the whole MSAs (grey strip below each one). In (**A**) the subaln. from arc. Ex1-encoded 16S rRNA, in (**B**) the subaln. from arc. Ex2-encoded 16S rRNA, in (**C**) the subaln. from bac. Ex1-encoded 16S rRNA, in (**D**) the subaln. from bac. Ex2-encoded 16S rRNA. In bacterial subalns., the antiSD sequence is marked by a red rectangle and the two essential adenines by a green rectangle.

All the 2D structures of 16S rRNA exhibit the predicted behaviour in terms of MFE values, in that the biological sequences have much lower MFE values than the average MFE values of the controls (Supplemental Material S2). Interestingly, the anti-SD sequence—a key feature of this molecule—appears to be more readily recovered by Ex2 triplets (Figure 2 and Supplemental Material S3).

According to the conservation profile, Ex2 triplets also seem to cover better the whole 16S rRNA sequences, than Ex1 triplets, in both arc. (low strip of Figure 2A,B) and bac. (low strip of Figure 2C,D). Supplemental Material S3 includes the corresponding 2D structures of consensuses, the conservation profiles, the sequence logos of all alignments, highlighting with rectangles the anti-SD (red) and the two adenines (green), as well as the predicted structure of bacterial 16S rRNA encoded by Ex1 triplets, which is highly similar to the experimental structure.

In particular, the region anti-SD (red rectangle) is absent in many archaea (taken from “Mejan”), and when present, is not completely encoded by Ex1 triplets (subaln. of Figure 2A) but by Ex2 triplets (subaln. of Figure 2B). The same behaviour is observed in bacteria, where the anti-SD is partially encoded by Ex1 triplets, but it is fully encoded by Ex2 triplets (red rectangles in Figure 2C,D). Meanwhile, the very essential adenines A1492 and A1493, are encoded by both ExGCs (narrow green rectangles in Figure 2C,D) in almost all 13 bacteria.

The 16S rRNA is well retrieved by both ExGCs from both types of organisms distinguishing the bacterial and archaeal variants, while the crucial anti-SD sequence is better captured by Ex2 triplets than by Ex1 in both archaea and bacteria. This rRNA has been widely used for decades as the “gold standard” molecular marker to discern between species and even subspecies and classify them because it shows a low enough evolutionary rate to be considered unique to every organism on the Earth [53]. Based on the characteristics of 16S rRNA, three kingdoms were proposed [54], and it has been used in the attempt to make the most complete tree of life [55], even if the organisms remain uncultured [56]. There is a patent intra-genomic heterogeneity of 16S rRNA genes on one hand and high similarity between such sequences from distantly related organisms on the other. This could result in taxonomic misclassifications, producing an overestimation of prokaryotic diversity [57,58,59].

### 3.3. 23S rRNA

The 23S rRNA, which contains the peptidyl transferase centre (PTC)—a sine qua non for protein translation—shows significantly better retrieval of this centre and the critical sites G2447 and A2451 when encoded by Ex2 triplets in both archaea and bacteria, compared to Ex1 triplets.

23S rRNA is approximately 2900 nt in length and constitutes the largest RNA molecule of the ribosome, located in the 50S subunit, where it interacts with some ribosomal proteins and other translation rRNAs [60,61]. Leaving aside the recent proposal that 23S rRNA has six domains rooted in a central zero domain [62], classically the molecule has been described to be folded into six domains, from which the domain V interacts with the tRNAs in the ribosomal sites A, P, and E during all translations [63]. Domain V is thus the crucial region of the 23S rRNA because it contains the catalytic peptidyl transferase centre (PTC) of ribosome [64,65]. The PTC is a ribozyme because the peptide bond occurs even in the absence of all ribosomal proteins [66]. Due to the universally conserved nt G2447 and A2451 (*E. coli* numbering), their catalytic functional role in the translation process was proposed. A2451 could change the charge distribution, providing a functional group that could act as a general acid-base catalyst (in the same way as the amino acid histidine in the catalytic active site of a classic protein enzyme) [67], and G2447 should play an important role in such catalysis. However, point mutations in both sites did not affect the translation rate [68,69]. This gave rise to the proposal that both G2447 and A2451 position the tRNAs, and form the entropic pocket to gear both reactants—the growing polypeptide chain and the charged tRNA [70]—in a controlled nearly water-free environment [71]. Actually, the only water-filled space is the exit tunnel, which is almost entirely lined with 23S rRNA and provides such ribosome surfaces with a water-resistant coat, enabling the synthesised peptide to slide easily while avoiding its preterm folding [72].

**Figure 3:**
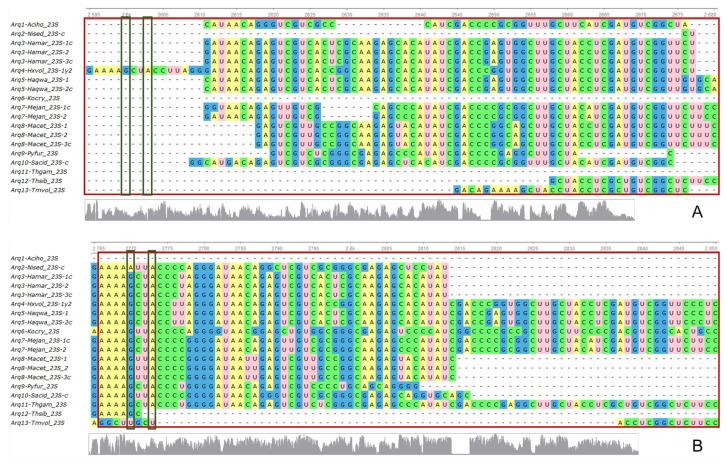

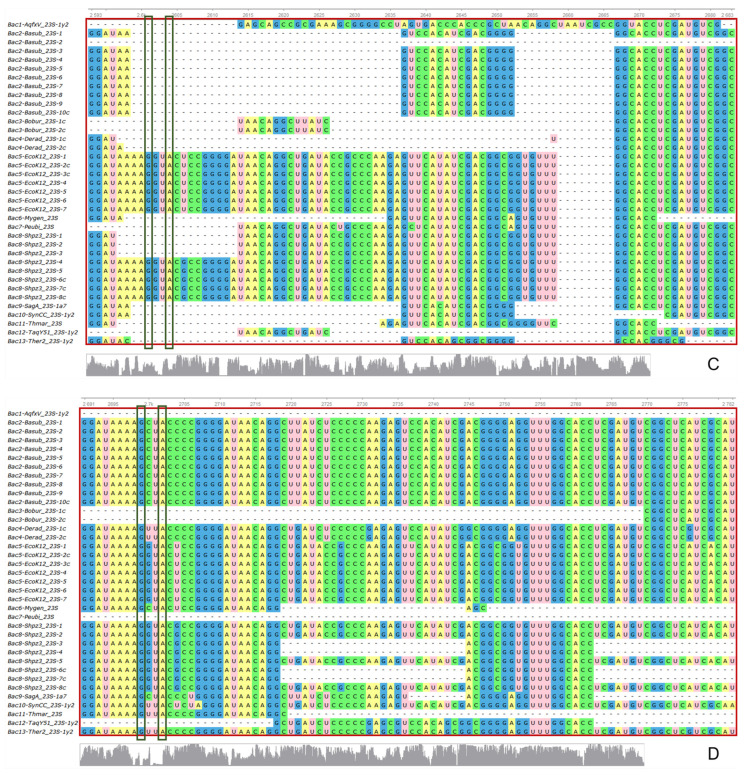
Subalignments (subalns.) extracted from the MSA of the ExGCs-encoded portion of 23S rRNAs PTC and the conservation profiles of the whole MSAs (grey strip below each one). In (**A**) the subaln. from archaeal Ex1-encoded 23S rRNA, in (**B**) the subaln. from archaeal Ex2-encoded 23S rRNA, in (**C**) the subaln. from bacterial Ex1-encoded 23S rRNA, in (**D**) the subaln. from bacterial Ex2-encoded 23S rRNA. In all subalns., the PTC sequence is marked by a red rectangle, with the two essential nucleotides G2447 and A2451 by green rectangles.

Regardless of the function of nt G2447 and A2451 in the PTC, it is undeniable they both are essential and universally conserved; remarkably, this key feature of 23S rRNA—the PTC including both sites aforementioned—is better recovered by Ex2 triplets (Figure 3). Regarding the MFE values, all 2D structures display the expected behaviour, with much lower MFE values of the biological ones than the averaged MFE values of controls (Supplemental Material S2). 

According to the conservation profiles, Ex2 triplets recover better the 23S rRNA sequences, than Ex1 triplets, in archaea (low strip of Figure 3A,B) and bacteria (low strip of Figure 3C,D). When the PTC from the model organism “Ther2” is aligned with the archaeal 23S rRNAs, it becomes more evident that Ex1 cannot capture this essential region (red rectangle in subaln. of Figure 3A), while Ex2 triplets (red rectangle in subaln. of Figure 3B) does it better. Remarkably the important nt G2447 and A2451 are barely encoded by Ex1 in bacteria or archaea (green narrow rectangles in subaln. of Figure 3A,B, respectively).

The same behaviour, with little better performance, is observed with bacterial 23S rRNA because Ex1 triplets can capture the PTC of only a few organisms, while it is almost covered by Ex2 triplets encoding; however, when the 23S rRNAs are aligned with PTC from bacteria “Ther”, some portions of the PTC are missing with the Ex1-encoding, while the PTC is completely retrieved if 23S rRNA is encoded by Ex2 triplets (red rectangle in subaln. of Figure 3C,D, respectively). The same happens with the very essential nt G2447 and A2451, encoded by both ExGCs but much better retrieved with Ex2 triplets than with Ex1 (enclosed in narrow green rectangles in Figure 3C,D).

The 23S rRNA, critical for translation, is slightly better retrieved in bacteria than in archaea; conspicuously, the PTC with its two pivotal sites G2447 and A2451 are much better retrieved by Ex2 triplets in both archaea and bacteria than Ex1 triplets. Supplemental Material S3 illustrates the 2D structures of consensuses and the sequence logos of all alignments, highlighting the full PTC with the two highly essential nucleotide positions. We were able to reconstruct the 3D predicted structure of the bacterial 16S rRNA encoded by Ex1-triplets, and we compared it with the crystallographic structure with PDB ID 6cao, and we discovered a high degree of similarity between the two (Supplemental Material S3). 

23S rRNA shows several INDELS along the sequence and has higher intragenomic diversity than 16S rRNA. However, each slightly different molecule of 23S rRNA in the same organism could help it to survive under different environmental conditions. Additionally, unlike the apparently more uniform 16S rRNA, the 23S rRNA cluster appears to be more different from organism to organism [73]. This could be the reason why 23S rRNA has been used lately as a coarser molecular marker, in addition to 16S rRNA, to identify and classify organisms in metagenomic studies [74].

### 3.4. 6S RNA

6S RNA, an abundant bacterial molecule that regulates gene expression by mimicking DNA structure, exhibits high sequence variability among species, making it challenging to obtain reliable consensus sequences and accurate structural predictions. While this outcome does not facilitate the elucidation of its evolution via Ex1 or Ex2 triplets, it does suggest that our methodology does not generate artefacts that consistently indicate favourable results.

Bacterial 6S is an abundant, not universal, RNA molecule of nearly 200 nt that binds to bacterial DNA-dependent RNA polymerase (DdRp) to regulate the expression of genes [75]. 6S is structured in three main regions: two long double-helical regions with at least 15 base pairs each, and one bulky large internal loop of 12 nt to 21 nt (median 15 nt) with low G content, which is highly variable among different species and therefore highly specific for the active site of each DdRp. There are also taxa-dependent small internal loops; the central bubble emulates unwound DNA at the promoter site [75,76,77]. The binding to DdRp occurs because 6S is not only highly similar to the open DNA that binds the DdRp for transcription [75], but it strictly mimics the same conformational B shape that DNA adopts when it binds to the DdRp. The larger stem of 6S resembles the same structure as DNA in promoter sites -35 and -10, while the central bulge bent over DdRp in an arrangement that is equal to the DNA helix that opens for the transcription process, including the Mg^2+^ presence, and 6S interacts with the same amino acids as DdRp does with DNA during transcription [78].

In the transition from the growth exponential phase to the stationary phase, 6S RNA is synthesised to modulate bacterial growth in response to nutrient deprivation or some other stress conditions [75,79]. 6S then binds to major DdRp to pause transcription of housekeeping genes (while other DdRps that function under stress conditions keep functioning). If stress conditions begin to reverse, cells can enter a new exponential growth phase. DdRp then synthesises short “product RNAs” (pRNAs) using 6S as a template. These short pRNAs bind unsteadily to the central bulge of 6S. When ATP molecules or some other NTPs are provided in high levels—by metabolism or external resupply—long pRNAs are synthesised. They attach more firmly to 6S and the complex completely disassembles from the DdRp, releasing it from inhibition. While the complex 6S:pRNA goes to degradation, the DdRp reassemble into a DNA promoter site [80]. This way, 6S RNA functions for long-term survival [76].

**Figure 4:**
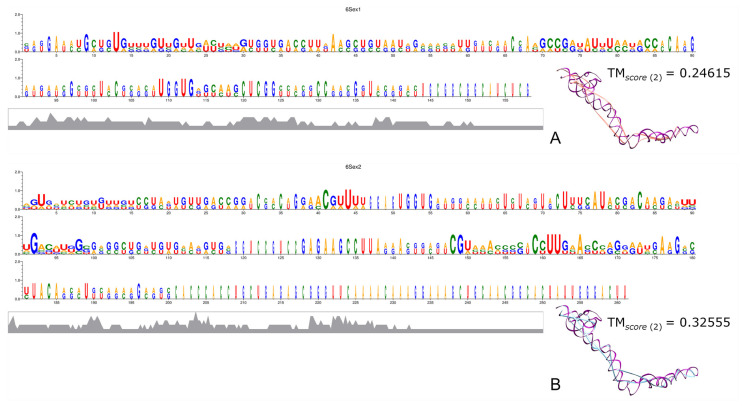
Logo sequences, conservation profiles of the whole MSAs (grey strip below each logo), and 3D structures of ExGCs-encoded 6S rRNAs. In (**A**) the Ex1-encoded 6S rRNA from bac., Ex1-encoded portion (salmon) is superimposed with current 5S rRNA (4ue4 purple, TM = 0.24615); in (**B**) the Ex2-encoded 5S rRNA from bac., Ex2-encoded portion (sky blue) is superimposed with current 5S rRNA (4ue4 purple, TM = 0.32555).

The many different 6S RNAs broadly present in bacteria have different accumulation patterns along the growth phase, and could even bind to different types of promoters. Additionally, they do not share strong sequence homology either inter-species or intra-specie, except for the structural central loop that binds to the active site of DdRp [76,77,79]. Despite the importance of this interesting molecule, the high variability among the bacterial 6S RNA sequences made it exceedingly difficult to generate reliable MSAs from which we could obtain reliable consensuses to compare with the crystallographic obtained structures (Figure 4). This also probably contributed to the fact that the MFE values of the biological sequences are higher (less stable structures) than the MFE values of their controls (Supplementary Material S2).

From the alignments of the fragments encoded by each ExGC, we obtained consensus sequences to predict 2D structures (see Supplementary Material S3), which apparently resemble the originals because they mostly exhibit self-complementary base-pairing, excluding only the formation of the main central bulge. Notwithstanding this, the consensuses came from mischievous MSAs, and we failed to obtain a 3D prediction when the corresponding crystallographic templates were used. We skipped the template-based structural reconstruction step; instead, we only aligned the consensus encoded by each ExGC to the experimentally determined structure (PDB ID 4ue4). As can be seen, although all structures and sequences come from 6S RNA molecules, the TM*_score_* indicates rather a random structural similarity (Figure 4A,B).

The poor MSAs and deficient structural reconstructions were expected due to the low sequence conservation that was previously reported [75,76,77,78,79,80], which in turn makes the 6S molecule unique and distinctive.

### 3.5. SRP-RNA

In general, for all the SRP-RNA types analysed (archaeal, small bacterial, and long bacterial with Alu insertion), Ex2 triplets tend to better conserve the sequence, especially in important core structural and functional regions. However, the specific importance of each type of triplet may vary depending on the specific region of the SRP-RNA. The central portion formed by key helices tends to be better conserved by Ex2 triplets in all SRP-RNA types studied, but the exact contribution may depend on the type of SRP-RNA and the helix in question.

The discovery and initial characterisation of the signal recognition particle (SRP) was worth a Nobel Prize to Günter Blobel in 1999 [81]. SRP is a GTP-dependent ubiquitous ribonucleoprotein that binds specific N-end marked proteins to cell membranes, while the ribosome still synthesises them [82,83,84]. The RNA-moiety of SRP (SRP-RNA) of all organisms have common structural and functional features; so, the eukaryotic 7SL RNA, the bacterial short 4.5S RNA of ~100 nt, the longer 6S RNA of ~270 nt from “Basub”, and the longest archaeal 7S RNA of ~300 nt [85], probably have an ancient common origin, given the universal conservation of all SRP components [86].

The SRP-RNA is composed of four structural domains, with human SRP-RNA as a reference [87]. It is arranged into 8 helixes [82] in such a way that helixes 1 to 4 shape domain I; helix 5 corresponds entirely to domain II; helixes 6 and helix 7 form domain III; and helix 8 corresponds to domain IV. Mammalian SRP-RNA is highly similar to the archaeal SRP-RNAs, albeit helix 7 is only present in humans [82] and some other eukarya contain four additional helixes (9 to 12) [88], the long “Basub” SRP-RNA lacks the domain III, and all the short bacterial SRP-RNAs only show the domain IV; this latter domain is conserved across all organisms, and therefore actually it constitutes the minimal SRP-RNA unit [82]. What is more, such a great marked difference between the small bacterial and all the other longer SRP-RNA is an Alu sequence, which gives the name to the domain formed by helixes 1 to 4 and the proximal half of the helix 5 (Alu domain), leaving the S domain to the rest of the molecule [82,84].

From the SRP database [83,84], we retrieved the MSA from the SRP-RNAs, labelled and curated according to its structural constitution. The order of helixes (H) in bacterial and archaeal SRP-RNA is as follows (H7 is omitted because it is exclusive to eukarya):

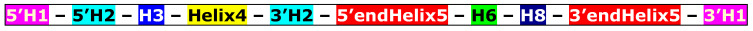



**Figure 5:**
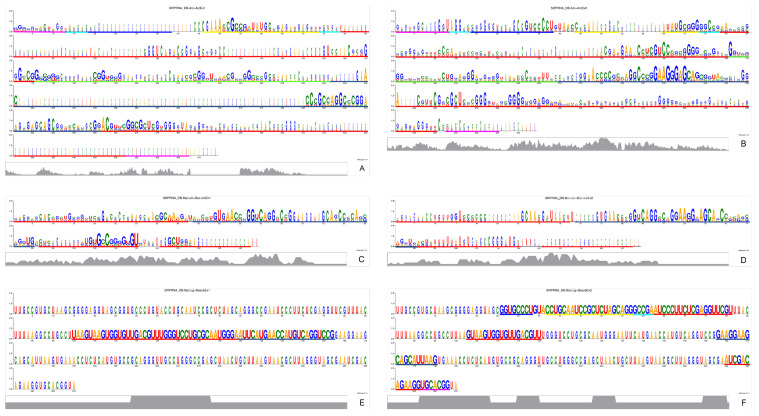
Logo sequences from the MSAs of ExGCs-encoded SRP-RNAs and the sequences in the corresponding database [83,84], as well as their conservation profiles (grey strip below each logo). In (**A**) the Ex1-encoded archaeal SRP-RNAs; in (**B**) the Ex2-encoded archaeal SRP-RNAs; in (**C**) the Ex1-encoded small bacterial SRP-RNAs; in (**D**) the Ex2-encoded small bacterial SRP-RNAs; in (**E**) the Ex1-encoded large bacterial SRP-RNAs; in (**F**) the Ex2-encoded large bacterial SRP-RNAs.

We obtained the consensuses from each MSA, and we aligned them with our sequences to visualise how each ExGC can reconstitute each type of SRP-RNA (Figure 5). We therefore used the same helix colour coding defined above in the sequence logos that we obtained.

#### 3.5.1. Archaeal SRP-RNA 

For archaeal SRP-RNA, sequences encoded by Ex2 triplets tend to be better conserved compared to those encoded by Ex1 triplets, particularly in key structural regions such as helix 5 and the central region of the SRP-RNA. This is consistent with the observation that Ex2 triplets tend to provide greater structural stability.

Archaeal SRP-RNA sequence logos are depicted in Figure 5A for Ex1-encoding and Figure 5B for Ex2-encoding. Regarding the stability of the archaeal SRP-RNA, the consensus sequence encoded by Ex1 triplets is less stable than the average of its controls, but the MFE values are as expected in the case of Ex2 triplets encoding the consensus sequence (Supplemental Material S2). When we reconstructed the 3D structure of the Ex1 consensus sequence (Supplemental Material S3), it was quite similar to the experimentally determined molecule (PDB ID 4uyk).

The 5′-end of helix 1 is mildly recovered by both ExGCs, while its 3′-end is practically not encoded by either. This helix has been proposed to latch the ends of this SRP-RNA, principally to prevent it from unfolding under extreme environmental conditions in modern organisms [87,88].

Helixes 2 and 3 have not been assigned to any relevant function [87,88], but we noticed that they are encoded by Ex2 triplets and minimally by Ex1 triplets in some organisms.

Helix 4 is mostly encoded by Ex1 triplets, while its encoding in Ex2 triplets is quite low. It is coaxial to helix 2 and shows the highest compensatory base changes (CBCs) among several organisms and with respect to all the other helixes [87,88], which provokes the highest sequence variations.

Helix 5 is probably the most important for the function of large SRP-RNAs, because its first half functions as the bridge between the Alu domain and the S domain, maintaining them separated to allow the proper interactions of the SRP proteins with the ribosome [87,88]. Accordingly, only the middle portion of this helix, divided by helixes 6 and 8, is encoded by both ExGCs in most of our archaea, with G and C as the most conserved nucleotides, while its 3′-end is not encoded by either of the ExGCs.

Archaea are the only prokaryotes whose SRP-RNA contains a helix 6 [87,88]. We found that this helix does not contain many Ex1 triplets, with only an initial G totally conserved. On the other hand, helix 6 has several portions encoded by Ex2 triplets, but none of them are totally conserved.

Only the last half of helix 8 has aligned portions encoded by Ex1 triplets, while it has more conserved portions encoded by Ex2 triplets. In both cases, G is the most conserved nucleotide, followed by C in the Ex1-triplet-encoded helix and A in the Ex2 triplet-encoded helix. This helix has regions abundant in purines, as well as noncanonical base pairs [87,88].

#### 3.5.2. Small Bacterial SRP-RNA 

In the case of the small bacterial SRP-RNA, the Ex2 triplets also seem to better preserve certain regions, such as the central domain and helix 8, which is critical for the function of the molecule. Meanwhile, the impact of the Ex1 triplets is lower, perhaps reflecting the lower complexity of this SRP-RNA.

Small bacterial SRP-RNA sequence logo encoded by Ex1 triplets is shown in Figure 5C and Ex2 triplets-encoding in Figure 5D. This version of SRP-RNA only contains helixes 5 and 8, the only ones preserved in all organisms, which is essential for the stabilisation of all SRP [87,88].

Helix 5 is the smallest of all helixes of the SRP-RNAs [82]. Given that bacterial small SRP-RNA lacks an Alu domain, this helix does not work as a bridge. The bacterial small SRP does not occlude the exit channel of the ribosome, and helix 5 barely contacts the nascent peptide chain [89]. It seems that small bacterial helix 5 does not fulfil a critical function, which coincides with the low conservation of the portion encoded by Ex2 triplets that we observed; while only a small fragment towards the 3′-end of this helix, encoded by Ex1 triplets, is conserved.

Small bacterial SRP does not occlude the exit tunnel of the ribosome or interfere with translation elongation factor G (EF-G), as do all the other large SRPs, yet it functions as an efficient carrier of the corresponding nascent proteins to the bacterial membrane [89]. The small bacterial SRP-RNA is vital in such processes [90,91]. In particular, helix 8 in its apical region [92] interacts with the protein moiety of the SRP, stabilising it [93,94], but also with the membrane receptor FtsY [95,96] and the EF-G by means of the conserved sequence GAAGCAGCCA [82,87,97,98]. It has been indicated that helix 8 could also interact directly with the N-terminal region of the signal peptide [99]. We observe that the middle portion of helix 8 is encoded by triplets belonging to both ExGCs, enriched with nucleotides A, G, and C, has very few T/U, and even the characteristic sequence GAAGCAGCCA can be partially recognised. This helix 8 is the only one universally conserved [86].

The bacterial small SRP-RNA biological consensus sequences, whether encoded by Ex1 or Ex2 triplets, have in both cases higher MFE values than their corresponding controls (Supplemental Material S2). Additionally, when we reconstructed the 3D structure of the Ex1 consensus sequence (Supplemental Material S3), it resembled the experimental molecule but was not very well conserved (PDB ID 3zn8).

#### 3.5.3. Large Bacterial SRP-RNA

For bacterial long SRP-RNA, again Ex2 triplets tend to be more prominent in coding important structural regions, such as helix 5 and parts of helix 8. 

Large bacterial SRP-RNA is present only in some Firmicutes and in a few Thermotogae [84]. In our bacterial group, only “Basub” has a large SRP-RNA. Our “Basub” sequences, with triplets of both ExGCs, were aligned with all strains of *Bacillus subtilis* in the database [83], and the results are depicted in Figure 5E for Ex1 triplets-encoding and Figure 5F for Ex2 triplets-encoding. In “Basub” SRP-RNA, only one region is encoded by Ex1 triplets and four fragments by Ex2 triplets.

Ex2 triplets encode a few nucleotides of the small helix 3 and some bases of the large helix 4, which interacts between them in tertiary structure [87,88], as well as all the five nucleotides of the contiguous 5′-end of helix 2. 

Helix 5 of “Basub” not only functions as a bridge between the Alu and S domains [87,88], but it is essential for SRP function and the formation of heat-resistant spores [100]. Additionally, a portion of helix 5, whose terminals meet with helixes 1, 2, 3, and 4, are encoded by Ex2 triplets. Together, they shape a compact structure in the Alu domain that binds the protein moiety of SRP. All such interactions probably shape a compact structure in the “Basub” Alu domain that binds with the protein moiety of SRP and the first bacterial histone-like described, which probably helps to condense “Basub” DNA [82,87,88]. By comparison, the only portion of helix 5 that is encoded by Ex1 triplets occurs proximal to helix 8, recalling that this segment of the former only seems to provide structural support to the latter helix.

Helix 8 is the only one conserved in absolutely all organisms [86]. It is important for the binding of SRP proteins [82], and the characteristic sequence GAAGCAGCCA in “Basub” [87] is virtually the only fragment encoded by Ex2 triplets and absolutely not by Ex1 triplets. Conversely, the portion encoded by Ex1 triplets of helix 8 is proximal to helix 5.

Finally, Ex2 triplets encode only 7 nt of helix 1 in its 3′-end, a helix that has been stated to fasten the SRP-RNA to stop it from unfolding under extreme environmental conditions [87,88].

Due to the proven high homology between Alu sequences, it has been stressed that DNA Alu elements, essential for eukaryotes in supercoiling its genetic material around histones to package nucleosomes, arise in the Alu moiety of the RNA of archaeal SRP [84,101]. The archaeal Alu sequence is highly homologous to “Basub” Alu that interacts with its histone-like proteins [100]. Hence, it is riveting to detect some portions of the Alu sequences encoded by both ExGCs in the large SRP-RNAs of archaea and bacteria.

Whether encoded by Ex1 or Ex2 triplets, the bacterial large SRP-RNA biological consensus sequences behave as expected, with their MFE values lower than those of their corresponding controls (Supplemental Material S2). In contrast, the consensus reconstructed structure encoded by Ex1 triplets is the least close to the experimental molecule when it comes to 3D reconstructions (Supplemental Material S3), while the structure encoded by Ex2 triplets is marginally better conserved (PDB ID 4wfl).

Supplemental Material S3 contains the 2D structures of consensus sequences, some 3D structures superposed with the crystallographic ones, and all the figures in a larger format.

### 3.6. tmRNA

For tmRNA, a dual-function RNA combining tRNA and mRNA properties, Ex2 triplets generally provide better conservation of larger tmRNA fragments compared to Ex1 triplets, both in sequence and to an even greater extent in structural reconstruction.

The tmRNA is a dual RNA with properties and structure of tRNA and mRNA combined in a single molecule and is present in diverse bacteria, yet is not essential. It weighs approximately 10 Svedberg units, which gives it its alternative name of 10Sa. It is a stable molecule, from what it takes its other alternative name: small stable RNA A (ssRA) [102]. The tmRNA is a highly structured complex molecule with numerous compensatory base changes (CBCs) and little identity in sequences from distantly related bacteria; one-third consists of the tRNA-like domain (TLD), one pseudoknot, and the mRNA-like domain (MLD) with seemingly well-defined functions, whereas two-thirds of the molecule consists of RNA helices and pseudoknots with largely unknown functions [103,104,105].

The tRNA-like domain (TLD) is a fully conserved minihelix of 35 nt, and is composed by the pairing of both the 5′-end and 3′-end of the RNA molecule. It has a T-arm without an anticodon or a D-arm, possesses characteristic modified nucleotides such as thymine and pseudouridine, and an acceptor stem CCA where one alanine is charged by the classic alanyl-tRNA ligase (AlaRL). This is probably because the AlaRL has the simplest tRNA recognition requirement of all tRNA ligases and tmRNA fulfils all requirements, such as an AA-stem that possesses a GU pair and ends with a CCA trinucleotide. After the TLD there is one essential pseudoknot (PK1) endonuclease resistant. After the PK1, 11 nt downstream, begins the mRNA-like domain (MLD), i.e., an open reading frame (ORF) that finishes with a stop codon. This is the least conserved feature in the middle of the structure. Such an ORF encodes a peptide with barely conserved positions and a variable length from 8 to 35 aa, which is also similar to some eukaryotic immunity proteins. Three to four additional pseudoknots link the MLD to the 3′-end of the TLD, although these pseudoknots (PK2, PK3, PK4, and PK5) are not conserved throughout all bacterial tmRNAs and seem to be dispensable for tmRNA function [103,104,106,107,108,109,110,111,112].

The dual function of tmRNA enables a quality control system called trans-translation, which consists of the rescue of a ribosome by substituting an original gene sequence by the ORF contained in the MLD. The newly encoded peptide, which begins with alanine and is highly rich in hydrophobic amino acids with two alanine residues near the end, is thus added to the originally synthesised protein, tagging it for its destruction by proteases. Though tmRNA is not essential in normal bacterial growth situations, it becomes so when the 3′-end of mRNA lacks a stop codon, during the translation of rare codons, when protein synthesis slowdowns or stalls, for bacterial survival under stress conditions such as amino acid starvation, or due to the absence of sufficient cognate tRNAs. Trans-translation is also used in different cell regulatory processes, as well as in enhancing bacterial pathogenesis [102,103,104,105,106,107,108,109,110,111,112]. Trans-translation process is achieved not only by tmRNA but also with the aid of not exclusive proteins, such as the aforementioned AlaRL, the translation elongation factor Tu (EF-Tu), the ribosomal protein S1 (S1), and the small protein B (SmpB, aka SsrA-binding protein), as well as the phosphoribosyl pyrophosphate synthetase (PrsA) and the ribonuclease R (RNAseR) [102,113].

When the 70S ribosome is stalled for any of the above reasons, tmRNA is charged by the AlaRL. Protein SmpB binds to the TLD, making tmRNA more stable and mimicking it as a regular tRNA. Because tmRNA lacks a D-loop and anticodon, the interaction TLD+SmpB also replaces the binding energy of codon-anticodon and so it enhances the tmRNA activity. Ala-tmRNA is charged, in complex with SmpB, onto the A site of the ribosome by EF-Tu by GTP hydrolysis, and the alanine is then transferred to the incomplete protein. The reading frame of MLD replaces the mRNA, and elongation resumes because the tmRNA translocates from the A site to the P site. The first pseudoknot PK1 arranges the correct positioning of the functional elements of tmRNA and S1 stabilises the entire molecule. This possibly exposes the resume codon for efficient trans-translation but without restricting the conformation inside the ribosome, where tmRNA adopts a more open structure. The tmRNA molecule thus enters the ribosome, despite its complicated topology. However, PK3 to PK5 remain outside of the ribosome during the peptidyl transfer and ORF translocation, otherwise the bulkage would distort the ribosome conformation. The original mRNA is then released from the ribosome, and the tmRNA becomes the template for the translation of the tagging sequence. The translation of MLD finishes and all the translational complex dissociates, releasing the tagged peptide that is finally degraded by proteolysis, whereas the translation components can be recycled [102,103,104,105,106,107,108,109,110,111,112,113,114,115,116,117,118,119,120,121].

Given TLD is the most conserved and MLD and PKs are the least conserved features of tmRNA, the alignments have been always achieved considering compensatory base changes (CBCs) and other covariations, and the secondary structures have been determined according to idiosyncratic features, not only by alignment [117,122,123]. Broadly speaking, tmRNA structures (based on *E. coli* numbering) are as follows [102]:

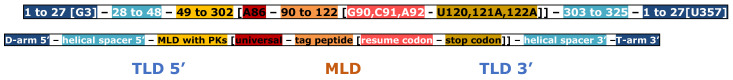



Blue hues indicate the TLD portion and the warm colours correspond to the MLD middle portion. Even though they could not be assigned to the sequence logos due to the considerable variation in the length of tmRNAs, an approximation was used to describe them.

**Figure 6:**
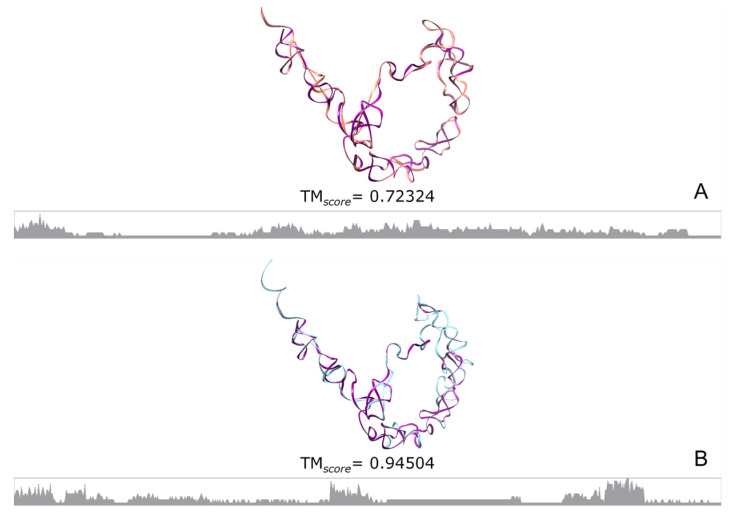
Conservation profiles (grey strip) of the MSAs of ExGCs-encoded tmRNAs, as well as a structural comparison of the reconstructed 3D structure and the crystallographic structure. In (**A**), the Ex1-encoded tmRNAs, Ex1-encoded portion (salmon) is superimposed with current tmRNA (3iyq purple, TM = 0.72324); (**B**) the Ex2-encoded tmRNAs, Ex2-encoded portion (sky blue) is superimposed with current 5S rRNA (3iyq purple, TM = 0.94504).

As expected, the tmRNA sequence logos in both ExGCs-encodings reveal patches of sparsely conserved portions (Figure 6). On one side, the logo of the tmRNAs (Figure 6A) encoded by Ex1 triplets has preserved the first 30 sites, as well as the portion from 150 to 170. Most of the other conserved positions are punctual scattered sites from nucleotide 200 to 400, like 201, 203, 310, 313, and so on. On the other side, the conserved positions of tmRNA in Ex2-encoding (Figure 6B) are rather well-clustered. Thus, we can observe the first 35 nt with several conserved nt, the portion from 46 to 61, the three A in 73 to 75, and a portion highly enriched in adenines from 275 to 300. Additionally, the portion from 479 to 493 has some conserved nt, and finally, the segment from 515 to 545 seems the most conserved.

It is important to point out that Ex1 triplets encode shorter and more distributed conserved sites, whereas Ex2 triplets encode larger and more conserved fragments of each tmRNA, yet the region unambiguously conserved by both encodings corresponds to the D arm of the TLD portion. In terms of the stability of bacterial tmRNA consensuses encoded by Ex1 or Ex2 triplets, we note that MFE values are as expected (Supplementary Material S2), though the Ex1-encoding consensus is nearly identical to the average of the controls. Interestingly, the 3D reconstruction is also closer to the crystallographic structure (PDB ID 3iyq) when dealing with the consensus sequence encoded by Ex2 triplets than Ex1 triplets, which is consistent also with the MSAs. Supplemental Material S3 contains the logo sequences of the MSAs and all the figures in a larger format.

Given both ends of tmRNA are conserved, though not its middle portion, it can be used in fluorescence in situ hybridisation (FISH) assessments to identify bacteria [124]. In fact, tmRNAs perform well as phylogenetic markers despite their small size because tmRNA sequences display considerable divergence, even within the same genera and intra-specie at the MLD, and their structural differences are reflected in their phylogenetic distribution, especially in more recent branches [102,107,124,125]. Although tmRNA is present in very ancient organisms [107], there are some bacteriophages that possess tmRNAs [123], and the tmRNA is similar to the 3′-end of certain eukaryal viral RNAs [106].

### 3.7. RNA-P

For the RNA component of ribonuclease P (RNA-P), an enzyme essential for cleaving immature tRNAs, Ex2 triplets generally provide better conservation of larger RNA-P fragments than Ex1 triplets. Notably, Ex2 triplets are the only ones to preserve the distinctive tetraloop GAAM and its context nucleotides, which are crucial for RNA-P function. However, sequence preservation by Ex2 triplets does not translate into more accurate structural reconstruction for either type of triplet, which is slightly more challenging for the Ex1 type. 

The RNA-based ribonuclease P (RNAseP) is a ubiquitous ribonucleoprotein. In modern prokaryotes, it is composed of a mildly dispensable protein portion and an essential RNA moiety (RNA-P) that cleaves immature tRNAs at its 5′-end by removing nucleotides from the 5′ end of the precursor molecule. RNA-P alone is catalytically active in vitro in bacteria and some archaea, [126], and therefore it is the only portion that we have focused on.

There are six types of prokaryotic RNA-based RNAsesP: three bacterial (A (ancestral, the majority), B (first described in *Bacillus*), and C (characteristic of *Chloroflexi*)), and three archaeal RNAseP (A (ancestral, the majority), M (first described in *Methanococci*), and T/P (characteristic of *Thermoproteaceae*)) [126,127,128,129,130,131].

RNA-P is a crucial component of RNAseP [132], constituting the catalytic subunit of the ribozyme [133] that recognises the 5′-end of immature tRNAs [134] with the help of divalent cations, (Mg^2+^) to achieve its catalytic activity [135]. RNA-P is about 350 to 450 nt in length with the sequence 5′-GAG**GAAM**GUCC-3′—where M stands for nucleotides in the amino tautomeric configuration, whether A or C—around 60 to 70 nt, as the major common characteristic [136], and with the tetraloop GAAA as the principal characteristic. The RNA-P moiety of bacterial RNAseP is catalytically active by itself at high ionic concentrations, unlike the archaeal RNAseP, which requires its protein counterpart to hydrolyse the tRNAs. At the same time, such proteins confer the RNA with chemical and physical protection against external degrading agents [129,137,138,139]. Structurally, RNAseP is disposed into five conserved regions (CR-I to CR-V), distributed into two domains (S and C). The RNA-P component of bacterial RNAseP is bigger than that of archaeal RNAseP, while the opposite happens with the protein component because bacterial RNAseP has only one small protein, and archaeal RNAseP has four or five proteins [129,140,141,142]. On one side, bacterial (Bac) RNAseP is subdivided into three groups: type A is dubbed because it is *A*ncestral, present in most bacteria (BacA), and its RNA-P is folded into 16 helixes. Type B is typical of lineage *Bacilli*, but also in some others such as *Streptococcus* and *Mycoplasma* to a minor extent, and its RNA-P is folded into 19 helixes (BacB) [143,144,145]. The C type of the phylum *Chloroflexi* has two smaller helixes, instead of the one present in type A, and lacks the GAAA tetraloop [146]. For its part, archaeal (Arc) RNA-P is folded into only 9 or 10 helixes [142,147,148]. Type A is the most prevalent among archaea, also named *A*ncestral consideration (ArcA). It is the most similar to bacterial type A [142,147,148], and even the most recent works cluster both types A together [129]. The type M (ArcM) exists only in phyla *Archaeoglobi* and *Methanococci* [148], and the structure of Mejan was experimentally determined by cryo-electron-microscopy (cryo-EM) [128]. The smallest RNA component of all the RNAses P was first identified in *Pyrobaculum* and was named type P [127]. However, after it became evident that it is characteristic of all the family *Thermoproteaceae*, its name was changed to type T [129]. Finally, there is one archaeon without the presence of RNAseP [149] because it lacks 5′ tRNA leaders [128], probably because it is an obligate symbiont of another archaeon.

**Figure 7:**
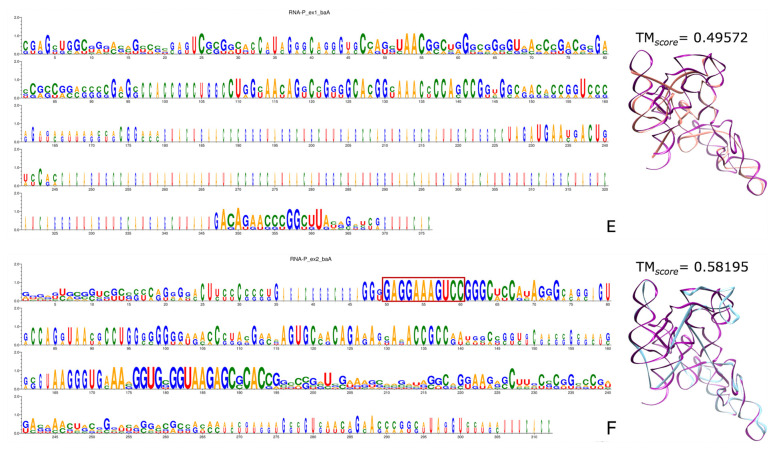
Logo sequences and 3D structures of bacterial type A RNA-P encoded by ExGCs. In (**E**) the RNA-P encoded by Ex1, Ex1-encoded portion (salmon) is superimposed with current RNA-P (3q1q purple, TM = 0.49572); in (**F**) the RNA-P encoded by Ex2, the characteristic sequence GAG**GAAM**GUCC is marked by a red rectangle in logo sequence, whereas Ex2-encoded portion (sky blue) is superimposed with current RNA-P (3q1q purple, TM = 0.58195).

For all the types of RNA-P, the MFE values of biological consensuses are consistently lower than the MFE values of their respective controls (Supplementary Material S3). Every figure in Section 3.7 is indicated with a letter S beside the corresponding consecutive letter; for example, Figure S7A is Figure 7A but is found in Supplementary Material S3, whereas Figure 7E is found here below.

According to all the sequence logos and conservation profiles of the RNA-P MSA (Figure 7), Ex1 triplets barely recover some common characteristics that form short specific clusters, and not even the tetraloop **GAAM** is identifiable in almost any of the Ex1-encodings. The worst performance occurs with the Ex1-encoding of the RNA-P from ArcA (Figure S7A), while ArcM (Figure S7C) and BacA (Figure 7E) performed little better. On the opposite side, Ex2 triplets encoded more of the RNAs-P where the characteristic sequence GAG**GAAM**GUCC is clearly distinguishable (red rectangles in all panels). The best results were seen in ArcA (Figure S7B), ArcM (Figure S7D), and BacA (Figure 7F), which were also acceptable. An intermediate situation can be found with the sequence of BacB, encoded by both ExGCs (Figure S7G,H). This is because both kinds of triplets encode the characteristic sequence GAG**GAAM**GUCC (red rectangles), although the Ex2-encoding is not reliable because the characteristic sequence is positioned in the wrong place, and the MSA is poor in both cases. The 3D reconstructions of the BacA consensus sequences are not particularly good when they are compared with the crystallographic structure, but the trend revealed with the MSAs regarding the Ex2-encoding (Figure 7F) is better than when Ex1 triplets (Figure 7E) are followed. It is important to recall that fragments encoded by both ExGCs from our sequences of ArcM and BacB were compared with the corresponding whole original sequences extracted from the database [126], precisely for the logo sequences to highlight the portions encoded by ExGCs.

In short, the RNA moieties of RNAseP (RNA-P) are homologous among organisms, but they are barely encoded by Ex1 triplets. Even the tetraloop GAAM into the sequence GAG**GAAM**GUCC is only encoded by Ex2 triplets. Also, the resemblance of RNA-P, at least the bacterial type A, to the crystallographic structure (PDB ID 3q1q) is barely satisfactory in both encodings, as indicated by the low TM*_score_*. However, it is better retrieved by Ex2 triplets.

Supplemental Material S3 contains all the sequence logos, conservation profiles, and the structures of consensus sequences (Figure S7A–H, with Figure S7A,B,G,H in Supplemental Material S3). We show some examples below (Figure 7E,F).

Based on the components and characteristics of RNAseP, it has been proposed that the primaeval RNAseP did not contain proteins, i.e., the ancient RNAseP was not a ribonucleoprotein but a pure ribozyme. As organisms evolved in complexity, the proteins gained an increasingly prominent role in the catalytic cleavage of immature tRNAs, until the emergence of a pure-protein enzyme, and the existence of organisms without any RNAseP at all [129,135,138,140,149,150,151].

### 3.8. tRNAs

In the study of tRNAs, the type of aa loaded onto different tRNAs seems to hold greater significance than the coding triplet (Ex1 or Ex2). While some tRNAs exhibit notable conservation in sequence and structure, the common pattern in 3D reconstructions varies depending on both the aa and the anticodon type. Ancient codon and aa incorporations are associated with higher recovery of tRNAs encoded by both ExGC triplets, with the anticodon frequently included, as observed in tRNAs for aa such as alanine, compared to more recently incorporated aa like tryptophan, which is somewhat less well recovered by ExGCs.

Francis Crick was the first to hypothesise the adaptor hypothesis for an RNA molecule that mediates the language between DNA (nt) and protein (aa) [152], and its presence was soon demonstrated [153]. Such an adaptor molecule is the smallest RNA and has been dubbed transfer RNA (tRNA). It is arranged into four double-helical stems and three single-stranded loops that generate a cloverleaf schema in 2D and compact L-shapes in 3D [72,154,155,156,157,158].

All tRNAs have a similar structure, which is well suited to their role as adaptors. The aa attachment occurs at the unpaired CCA 3′-end of the acceptor arm, facilitated by an aminoacyl-tRNA ligase (aaRL). Meanwhile, the middle portion of the L-shape contains the anticodon, which interacts with a specific codon in an mRNA, by virtue of its complementary and antiparallel nature. This allows for a wobble between the first base of the anticodon and the third codon position, yet maintains the correct reading frame in sequential sets of 3 nt. Although some tRNAs are missing portions, the anticodon and acceptor arms are always retained [65,72,154,155,156,157,158,159,160,161].

In the SGC, 61 out of the 64 possible trinucleotides code for a specific aa (whereas UAA, UGA, and UAG encode for none) [159]. Because of nonstandard interactions like wobbling, a tRNA may base-pair with more than one mRNA codon, and conversely, one codon may base-pair with multiple tRNAs, a phenomenon dubbed degeneracy or redundancy of the genetic code. The different tRNAs that accept the same aa but with different anticodon are called isoacceptors [72,154,155,159,161].

The tRNAs constitute adaptor molecules that form the link between the mRNA and the polypeptide that is being synthesized. It is both a physical link (operational code) in the attachment between the polypeptide and the nucleic acids and an informational link (informational code) in the tRNA carrying the aa specified by the codon to which it attaches. Such specificity ensures that protein synthesis follows the rules of the genetic code, and the attachment of a given aa to a particular tRNA establishes the genetic code [154,158].

The vast majority of the MFE values of the biological consensus and the average of their controls behave as expected, with the exception of a few cases where the average MFE value of the controls is lower than the biological consensus MFE value. In a few cases, both types of sequence have almost equal stability.

**Figure 8:**
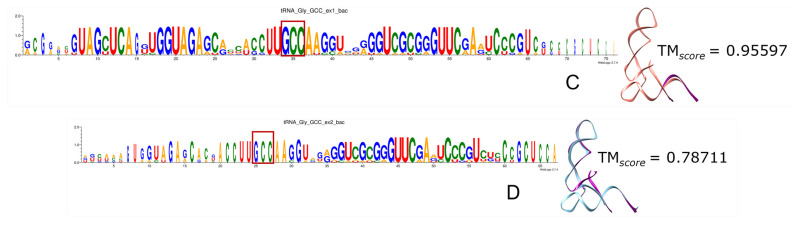
Logo sequence of the MSA of the ExGCs-encoded bacterial Gly-tRNA and the corresponding 3D reconstruction, with the anticodon GCC highlighted by a red square. In (**C**) the Ex1-encoded Gly-tRNA (salmon) is superimposed with current Gly-tRNA (4mgn purple, TM = 0.95597); in (**D**) the Ex2-encoded Gly-tRNA (sky blue) is superimposed with current Gly-tRNA (4mgn purple, TM = 0.78711).

**Figure 9:**
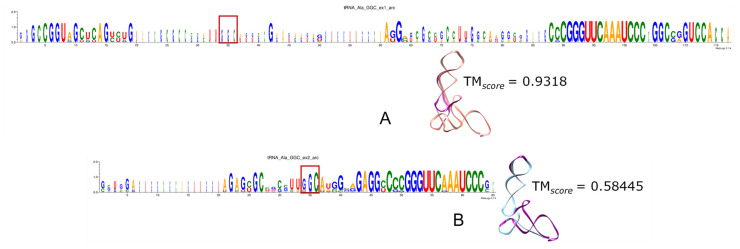
Logo sequence of the MSA of the ExGCs-encoded bacterial Ala-tRNA and the corresponding 3D reconstruction, with the anticodon GGC highlighted by a red square. In (**A**) the Ex1-encoded Gly-tRNA (salmon) is superimposed with current Gly-tRNA (3wqz purple, TM = 0.9318); in (**B**) the Ex2-encoded Gly-tRNA (sky blue) is superimposed with current Gly-tRNA (3wqz purple, TM = 0.58445).

**Figure 10:**
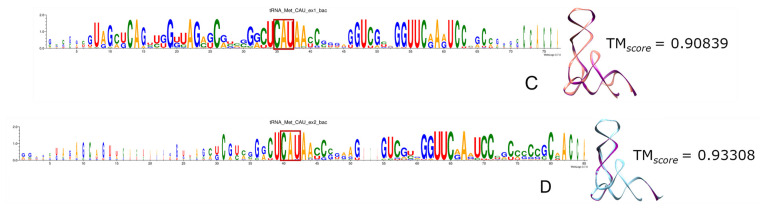
Logo sequence of the MSA of the ExGCs-encoded bacterial Met-tRNA and the corresponding 3D reconstruction, with the anticodon CAU highlighted by a red square. In (**C**) the Ex1-encoded Met-tRNA (salmon) is superimposed with current Met-tRNA (2csx purple, TM = 0.90839); in (**D**) the Ex2-encoded Met-tRNA (sky blue) is superimposed with current Met-tRNA (2csx purple, TM = 0.93308).

**Figure 11:**
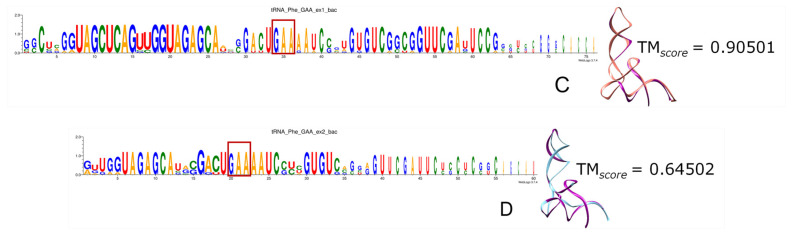
Logo sequence of the MSA of the ExGCs-encoded bacterial Phe-tRNA and the corresponding 3D reconstruction, with the anticodon GAA highlighted by a red square. In (**C**) the Ex1-encoded Phe-tRNA (salmon) is superimposed with current Phe-tRNA (3l0u purple, TM = 0.90501); in (**D**) the Ex2-encoded Phe-tRNA (sky blue) is superimposed with current Phe-tRNA (3l0u purple, TM = 0.64502).

**Figure 12:**
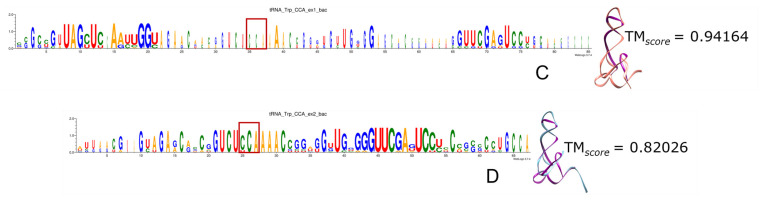
Logo sequence of the MSA of the ExGCs-encoded bacterial Trp-tRNA and the corresponding 3D reconstruction, with the anticodon CCA highlighted by a red square. In (**C**) the Ex1-encoded Trp-tRNA (salmon) is superimposed with the current Trp-tRNA (4ycp purple, TM = 0.94164); in (**D**) the Ex2-encoded Trp-tRNA (sky blue) is superimposed with current Trp-tRNA (4ycp purple, TM = 0.82026).

Remarkably, the more ancient the incorporation of specific codons and aa to the genetic code, the higher the portion of tRNAs recovered by triplets of both ExGCs. Most frequently the anticodon is included, as we can see in the logo sequences, where anticodon (anc.) is highlighted in red rectangles, and in the 3D reconstructions when compared with the modern experimentally determined tRNA molecules, whether from bacteria (bac.) or from archaea (arc.). From all tRNAs, the nucleotide positions retrieved include the identity elements correlated with the anticodon and contribute to the specificity of their aminoacyl-tRNA ligases (a.k.a. aminoacyl-tRNA synthetases) [162,163]. Supplementary Material S3 contains all the logo sequences that we obtained, with the anticodon highlighted by a red square, and 2D and 3D structures of consensuses (Figure 8, Figure 9, Figure 10, Figure 11 and Figure 12). We show some examples below. Every figure in Section 3.8 is indicated with a letter S beside the corresponding consecutive letter; for example, Figure S8A is Figure 8A but is found in Supplementary Material S3, whereas Figure 8C is found here below.

First, we have tRNAs charged by prebiotic aa and whose anc. follows the RNY pattern. These tRNAs are among the best-conserved sequences, and they include the anticodon totally. For example, Gly_GCC is shown in Figure S8A–D, and Ala_GGC in Figure 9A,B and Figure S8C,D, along with Asp_GUC and Val_GAC. We can see some exceptions in archaeal Gly-tRNA and Ala-tRNA because they diverge enough not to show a high degree of conservation, and even the anticodon is poorly retrieved. Regarding structures from both bacterial Gly-tRNA and archaeal Ala-tRNA, Ex1 reconstructions are more similar to the experimental molecule than the Ex2 reconstructions.

Among the aa whose triplets belong to both ExGCs, we found histidine and cysteine methionine. An interesting case is found for Met-tRNA sequences, whose codon AUG belongs to both ExGCs because they even exhibit the highest inter- and intra-species divergence, the anticodon CAU is retrieved from all sequences and MSAs can be performed. The MSA shows better conservation with Ex1 triplets than with Ex2 in both archaea and bacteria; however, the opposite case occurs with the bacterial 3D reconstructions because there is slightly lower conservation with Ex1-encoding than with Ex2 (Figure S10A–D). The large variability in Met-tRNAs could be to ensure that all proteins that start with Met do so, regardless of the environmental or physiological conditions of the organisms, or it could be the result of convergent evolution, or many other possibilities [164].

Phenylalanine and tyrosine are examples of aa encoded only by Ex2 triplets. The Phe-tRNAs are more or less encoded by triplets of both ExGCs. However, the bacterial molecule shows better sequence conservation than the archaeal one, for which even the anticodon GAA is barely captured. Moreover, the reconstruction encoded by Ex1 triplets is much more similar to the experimental structure than to the Ex2-encoding reconstruction (Figure S11A–D).

Tryptophan is one of the aa exclusively encoded by Ex1 genetic code triplets, but the anticodon CCA is hardly encoded at all, and just a few organisms encode the gene. Given the small portions encoded by both ExGCs, we observe in general a low degree of conservation, although the fragments encoded by triplets Ex1 are shorter than Ex2 encoded fragments. Noting subtle differences, we observe that the logo sequence of bacterial Trp-tRNA encoded by Ex2 triplets exhibits the best-conserved portions, but the 3D structures indicate a better reconstruction with Ex1 triplets than with Ex2 (Figure S12A–D).

We noticed that almost no tRNA have A in the 5′ wobble position of the anticodon, which has been explained previously for the three domains of life as a way to avoid a perfect complementary pairing which could halt the translation process [165,166]. Due to the same cloverleaf pattern in all tRNAs, it has been suggested that they all had a common evolutionary origin [154] that predates the progenote [167].

### 3.9. C/D Box Small Nucleolar RNAs

The C/D box small nucleolar RNAs, which guide RNA methylation [168], are partially encoded by triplets of both ExGCs. However, due to significant differences among these C/D box RNAs, they are unsuitable for comparative analyses. C/D box RNAs have been reported in several archaea that we selected [168]. However, according to our data, only “Pyfur” contains such type of RNA, and the 16 sequences are different to each other.

## 4. RNA Beginnings and Evolution before LUCA: Discussion

RNA is one of the most versatile of all the biological macromolecules, probably the molecule which is behind the origin of the genetic code, dating back to ~4.36 ± 0.1 billion years ago [2,3,4,5,6]. Due to its two error-prone properties of storing information and catalysing mechanisms such as self-excision, RNA was considered the first biological molecule from which life emerged on a hypothetical ‘RNA world’, before the appearance of catalytic proteins on the one side and DNA for inheritance on the other side, as separate biomolecules [133,169,170].

Such primaeval genetic code began with only 16 triplets following the pattern RNY, and the earliest RNA molecules folded as hairpins or mini-helixes [12]. The PGC evolved via two distinct pathways, Ex1 and Ex2, which gave rise to molecules that look much more like modern RNAs in most cases. This “extended RNAome”, encoded by the ExGCs, formed at the dawn of the Last Universal Common Ancestor (LUCA), and we have been able to delineate it.

By examining the results, it becomes clear that the most significant and evident common denominator of this study is the ability of the extended genetic code 2 (Ex2) to more effectively capture crucial characteristics of the various RNA molecules analysed. This is generally true for 5S rRNA, as well as 16S rRNA, where the anti-SD sequence is only recovered by Ex2 triplets. The same applies to the PTC, with its two critical positions, G2447 and A2451, being almost exclusively recovered by Ex2 triplets. Likewise, the common quadruplet GAAM of RNA-P is only recovered by Ex2 triplets, and the dual molecule tmRNA is better recovered by Ex2 triplets. However, in the case of SRP-RNA, both types of triplets seem to recover the molecules similarly, albeit slightly better with the Ex2 genetic code. Although the recovery of the 6S molecule was also better with Ex2 triplets, this observation should be approached with caution due to the overall poor recovery.

On the other side, retrieving tRNAs does not rely on what type of ExGC the tRNA molecule encodes, but rather on the antiquity of incorporation of the tRNA-activating aa into the genetic code, which means that the more ancient is the incorporation of the corresponding aa, the better the recovery of the tRNA molecule. For example, glycyl-tRNA (Gly-tRNA) is better recovered than triptophanyl-tRNA (Trp-tRNA), because Gly was among the first aa to be incorporated into the genetic code and Trp among the latter [171,172].

In most cases, the free energy of biological sequences is lower than their controls. Looking at the logos, which graphically show the outcome of the MSAs, it is also noticeable that the fragments encoded by each of the two types of ExGCs appear to be complementary to each other.

Distinctively, the more ancient an RNA molecule is—i.e., a molecule that has its primordia in the PGC [12]—the more probable it was nearly complete before LUCA, but if it is encoded only with ExGCs triplets, the molecule will not look so much like the corresponding modern RNA. Based on our results herein presented, we should stress that most of the molecules that were rather fixed along evolution due to their usefulness were mostly derived from Ex2 triplets, such as the PTC or the anti-SD sequence, while the Ex1 triplets complemented them as if they were to fill in the spaces, whereas in the case of tRNAs, Ex1 and Ex2 triplets just seem to complement each other without any obvious bias in functionality. Previously, RNA families involved in protein synthesis and export, i.e., rRNAs, tRNAs, RNA-P, and SRP-RNA, were only traced to the last common ancestor [173], but now we can observe that their origins can be traced to the dawn of the LUCA. 

We emphasize that the current SGC did not appear in a single stroke of nature’s creation. Rather, it started from the recognizable RNY pattern from which it evolved two intermediate stages before the complete code—the SGC—was formed. The PGC based on RNY polynucleotides [7] that evolved by the two different complementary pathways ExGCs to finally shape the current SGC [8,9] is an equivalent model to the Rodin–Rodin–Ohno hypothesis on the evolution of genetic code [174]. It may be possible that initial metabolisms could have driven the subsequent formation of the genetic code [175,176], but at some point, a genetic code must have prevailed that allowed the advantageous attributes to be conserved and inherited more efficiently. Be that as it may, it is revealed that the LUCA’s ancestors were already highly complex entities and horizontal gene transfer (HGT) was prevalent among the earliest life forms [177].

According to the evolution of the genetic code model starting from a PGC of RNY to a dichotomous pathway of ExGCs, what we obtained are the RNA molecules before its final constitution. It is noteworthy to point out that with the Ex2, we identified the PTC, which is the key molecule for the encoding of proteins and the capacity of inheritance. This stage is known as FUCA [178,179,180,181]. Thereby, the RNA sequences encoded by triplets pertaining to each ExGC complemented each other and therefore may have assembled cooperatively instead of competing. Meanwhile, a new and more stable genetic molecule began to emerge (DNA), and proteins took the role of main structures with functionalities. Eventually, it shaped the whole RNAome of the LUCA.

These findings provide a basis for future research into the evolution of the genetic code and the function of RNA in different domains of life. On the other side, by exploring the diversity of RNA processing pathways in modern organisms, we can infer some evolutionary pressures that may have shaped the RNA world, including the selection of robust and versatile RNA processing systems capable of responding to fluctuating environments, potentially offering clues into how early life forms managed environmental challenges and laid the foundation for more complex cellular responses [182].

Understanding the emergence of life before the LUCAs (assuming commonalities instead of a single primordial organism) involves investigating the interplay of RNA and diverse molecular mechanisms, whereas understanding RNA’s evolutionary history is critical to resolving the origins of life’s molecular complexity [183].

## 5. Conclusions

While the outcomes of the present work need to be proven in wet lab conditions, this study emphasises the importance of RNA as a pivotal molecule in the establishment of genetic code. Moreover, it suggests that Ex2, formed by transversions in the first or third base of the RNY-based PGC, led such evolution before LUCA, with Ex1 complementing. However, as Ex1 is the only code that introduced stop codons, it should have been necessary for the establishment of the genetic code, giving it completion. Seeing it as a fabric, the Ex1 triplets provided the final stitches to make it consistent and provide an end that started with the RNA’s RNY pattern.

Evolution lacks foresight, and the transition from prebiotic to biotic is blurred. Likewise, the transition from non-coding to coding is a monumental task to define. It may be the case that older RNA molecules possessed different functions from current molecules, and the RNA molecules analysed in this work must be regarded as potentially relevant biological molecules that would eventually become essential components of the translation apparatus: What is available to evolution is what it uses to create novelty. Understanding these processes may illuminate the origins of life and the subsequent emergence of the ToL.

## Figures and Tables

**Table 1:** The names of the organisms that we employed for this work are listed with their corresponding abbreviation, “Abbrev.”.

Bacteria		Archaea	
Organism’s name	Abbrev.	Organism’s name	Abbrev.
*Aquifex aeolicus* VF5	“AqfxV”	*Acidianus hospitalis* W1	“Aciho”
*Bacillus subtilis* 168	“Basub”	*Haloarcula marismortui* ATCC43049	“Hamar”
*Borreliella burgdorferi* B31	“Bobur”	*Haloferax volcanii* DS2	“Hxvol”
*Deinococcus radiodurans* R1	“Derad”	*Haloquadratum walsbyi* DSM16790	“Haqwa”
*Escherichia coli* K12	“EcoK12”	*Korarchaeum cryptofilum* OPF8	“Kocry”
*Mycoplasma genitalium* G37	“Mygen”	*Methanocaldococcus janashii* DSM 2661	“Mejan”
Ca. *Pelagibacter ubique* HTCC1062	“Peubi”	*Methanosarcina acetivorans* C2A	“Macet”
*Shewanella piezotolerans* WP3	“Shpz3”	Ca. *Nitrosopumilus sediminis* AR2	“Nised”
*Streptococcus agalactiae* A909	“SagA”	*Pyrococcus furiosus* DSM3638	“Pyfur”
*Synechococcus* CC9902	“SynCC”	*Sulfolobus acidocaldarius* DSM639	“Sacid”
*Thermotoga maritima* MSB8	“Thmar”	*Thermococcus gammatolerans* EJ3	“Thgam”
*Thermus aquaticus* Y51MC23	“TaqY51”	*Thermococcus sibiricus* MM739	“Thsib”
*Thermus thermophilus* HB8	“Ther2”	*Thermoplasma volcanium* GSS1	“Tmvol”

## Data Availability

The original contributions presented in the study are included in the article/Supplementary Material, further inquiries can be directed to the corresponding author.

## Supplementary Materials

The following supporting information can be downloaded at: https://www.mdpi.com/article/10.3390/genes15091195/s1, S1 contains a graphical flowchart of the methodology followed in this work. S2 contains the tables comparing the MFE values of the biological sequences vs the average of their controls. S3 contains all the RNA sequence logos, 2D structures, conserved profiles, sub-alignments, 3D structures, and structural alignment figures declared in this work.
