# Supplementary material for: A Proposal for the RNAome at the Dawn of the Last Universal Common Ancestor
Source: Genes (Basel). 2024 Sep 11;15(9):1195. doi: 10.3390/genes15091195 (PMC11431127; doi:10.3390/genes15091195)
Supplement: Supplementary file 1 [file genes-15-01195-s001.zip › S3. AllFigures_RNAsbeforeLUCA_aug2024.pdf]

S3. Supplementary material for

A proposal of the RNAome to the very dawn of LUCA

Miryam Palacios-Pérez [a] & Marco V. José [a,*]

[a] *Theoretical Biology Group, Instituto de Investigaciones Biomédicas, Universidad Nacional Autónoma de México, Ciudad de México CDMX, C.P. 04510, México*
First author E-mail address: mir.pape@iibiomedicas.unam.mx
[*] *Corresponding author E-mail address*: marcojose@biomedicas.unam.mx

1.- 5S rRNA, ExGCs
- A.- Archaea, Ex**1**: logo, 2D, 3D, comparison, and conservation profile (cons. prof.).
- B.- Archaea, Ex**2**: logo, 2D, 3D, comparison, and cons. prof.
- C.- Bacteria, Ex**1**: logo, 2D, 3D, comparison, and cons. prof.
- D.- Bacteria, Ex**2**: logo, 2D, 3D, comparison, and cons. prof.

2.- 16S rRNA, ExGCs
- A.- Archaea, Ex**1**: subaln. with anti-SD, 2D, cons. prof.and logo with anti-SD.
- B.- Archaea, Ex**2**: subaln. with anti-SD, 2D, cons. prof., and logo with anti-SD.
- C.- Bacteria, Ex**1**: subaln. with anti-SD, 2D, cons. prof., logo with anti-SD, and 3D.
- D.- Bacteria, Ex**2**: subaln. with anti-SD, 2D, cons. prof., and logo with anti-SD.

3.- 23S rRNA, ExGCs
- A.- Archaea, Ex**1**: subalignment (subaln.) with PTC, 2D, cons. prof., and logo with PTC.
- B.- Archaea, Ex**2**: subaln. with PTC, 2D, cons. prof., and logo with PTC.
- C.- Bacteria Ex**1**: subaln. with PTC, 2D, cons. prof., and logo with PTC.
- D.- Bacteria Ex**2**: subaln. with PTC, 2D, cons. prof., and logo with PTC.

4.- 6S RNA, ExGCs
- A.- Bacteria, Ex1: logo, cons. prof., 2D, and 3D
- B.- Bacteria, Ex2: logo, cons. prof., 2D, and 3D

5.- SRP, ExGCs
- A.- Logo of ConsensusArchaea(DB)+ Archaea_Ex**1**, coloured as DB; 2D, cons. prof., and 3D.
- B.- Logo of ConsensusArchaea(DB)+ Archaea_Ex**2**, coloured as DB; 2D, cons. prof.
- C.- Logo of ConsensusBacteriaSmall(DB) + BacteriaSmall_Ex**1**, coloured as DB; 2D, 3D, and cons. prof.
- D.- ConsensusBacteriaSmall(DB) + BacteriaSmall_Ex**2**, coloured as DB; 2D, 3D, and cons. prof.
- E.- Logo of ConsensusBacterialLarge-Basub(DB)+BacteriaLarge-Basub_Ex**1**, coloured as DB; 2D, 3D, and cons. prof.
- F.- Logo of ConsensusBacterialLarge- Basub(DB)+BacteriaLarge-Basub_Ex**2**, coloured as DB; 2D, 3D, and cons. prof.

6.- tmRNA, ExGCs
- A.- Bacteria, Ex**1**: logo, cons. prof, 2D, and 3D
- B.- Bacteria, Ex**2**: logo, cons. prof, 2D, and 3D

7.- RNA-P, ExGCs
- A.- Archaea type A, Ex**1**: logo, 2D, and cons. prof.
- B.- Archaea type A, Ex**2**: logo with tetraloop sequence, 2D, and cons. prof.
- C.- Archaea type M, Ex**1**: logo, 2D, and cons. prof.
- D.- Archaea type M, Ex**2**: logo with tetraloop sequence, 2D, and cons. prof.
- E.- Bacteria type A, Ex**1**: logo, 2D, 3D, and cons. prof.
- F.- Bacteria type A, Ex**2**: logo with tetraloop sequence, 2D, 3D, and cons. prof.
- G.- Bacteria type B, Ex**1**: logo, 2D, and cons. prof.
- H.- Bacteria type B, Ex**2**: logo with tetraloop sequence, 2D, and cons. prof.

8.- tRNA-Gly_GCC, ExGCs
- A.- Archaea, Ex**1**: logo with anticodon, and 2D
- B.- Archaea, Ex**2**: logo with anticodon, and 2D
- C.- Bacteria, Ex**1**: logo with anticodon, 2D and 3D
- D.- Bacteria, Ex**2**: logo with anticodon, 2D and 3D

9.- tRNA-Ala_GGC, ExGCs
- A.- Archaea, Ex**1**: logo with anticodon, 2D and 3D
- B.- Archaea, Ex**2**: logo with anticodon, 2D and 3D
- C.- Bacteria, Ex**1**: logo with anticodon, and 2D
- D.- Bacteria, Ex**2**: logo with anticodon, and 2D

10.- tRNA-Met_CAU, ExGCs
- A.- Archaea, Ex**1**: logo with anticodon, and 2D
- B.- Archaea, Ex**2**: logo with anticodon, and 2D
- C.- Bacteria, Ex**1**: logo with anticodon, 2D and 3D
- D.- Bacteria, Ex**2**: logo with anticodon, 2D and 3D

11.- tRNA-Phe_GAA, ExGCs
- A.- Archaea, Ex**1**: logo with anticodon, and 2D
- B.- Archaea, Ex**2**: logo with anticodon, and 2D
- C.- Bacteria, Ex**1**: logo with anticodon, 2D and 3D
- D.- Bacteria, Ex**2**: logo with anticodon, 2D and 3D

12.- tRNA-Trp_CCA, ExGCs
- A.- Archaea, Ex**1**: logo with anticodon, and 2D
- B.- Archaea, Ex**2**: logo with anticodon, and 2D
- C.- Bacteria, Ex**1**: logo with anticodon, 2D and 3D
- D.- Bacteria, Ex**2**: logo with anticodon, 2D and 3D

Notes

The value Tm_{score} can be given in relation to the target (1), that in the present work corresponds to each of the consensuses, or in relation to the template (2). Since all the consensuses are compared with the same template per RNA molecule, we provide TM_{score} 2. For the sake of clarity, in the main text the value is only referred as TM_{score}.

Ex1-encoded portions are in salmon, Ex2-encoded portions are in sky blue. The modern structures are in purple.

Current structures (PDBcode_chain) used for comparison:

Archaeal 5S rRNA 1ffk_9
Bacterial 5S rRNA 5gaf_B

Bacterial 16S rRNA 6cao_A

Bacterial 6S RNA 4ue4_A

Archaeal SRP 4uyk_R
Bacterial small SRP 3zn8_G
Bacterial large SRP 4wfl_A

Bacterial tmRNA 3iyq_A

Bacterial RNA-P type A 3q1q_B

Bacterial Gly-tRNA_GCC 4mgn_D

Archaeal Ala-tRNA_GGC 3wqz_C

Bacterial Met-tRNA_CAU 2csx_D

Bacterial Phe-tRNA_GAA 3l0u_A

Bacterial Trp-tRNA_CCA 4ycp_B

rRNAs

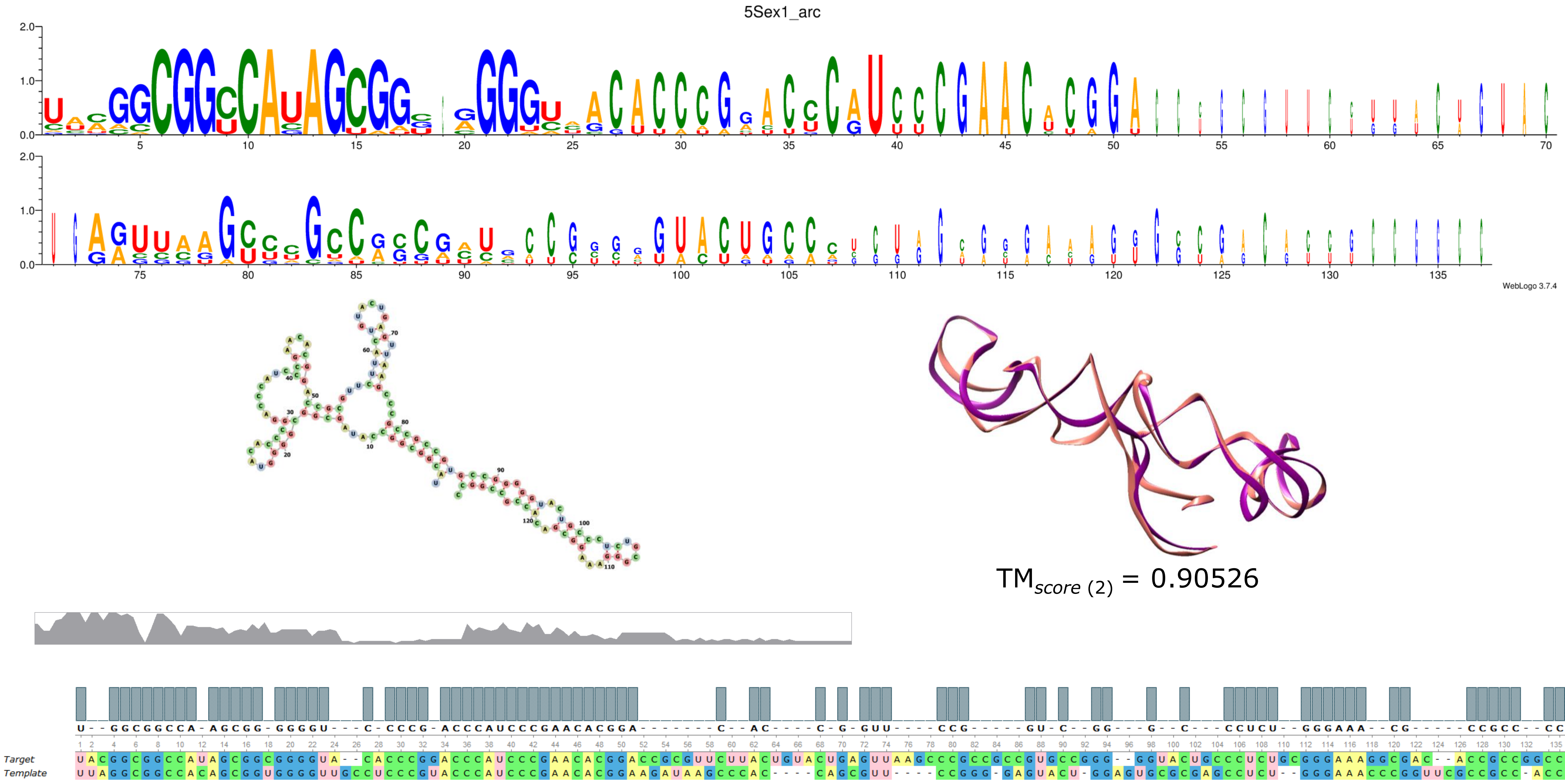

1A

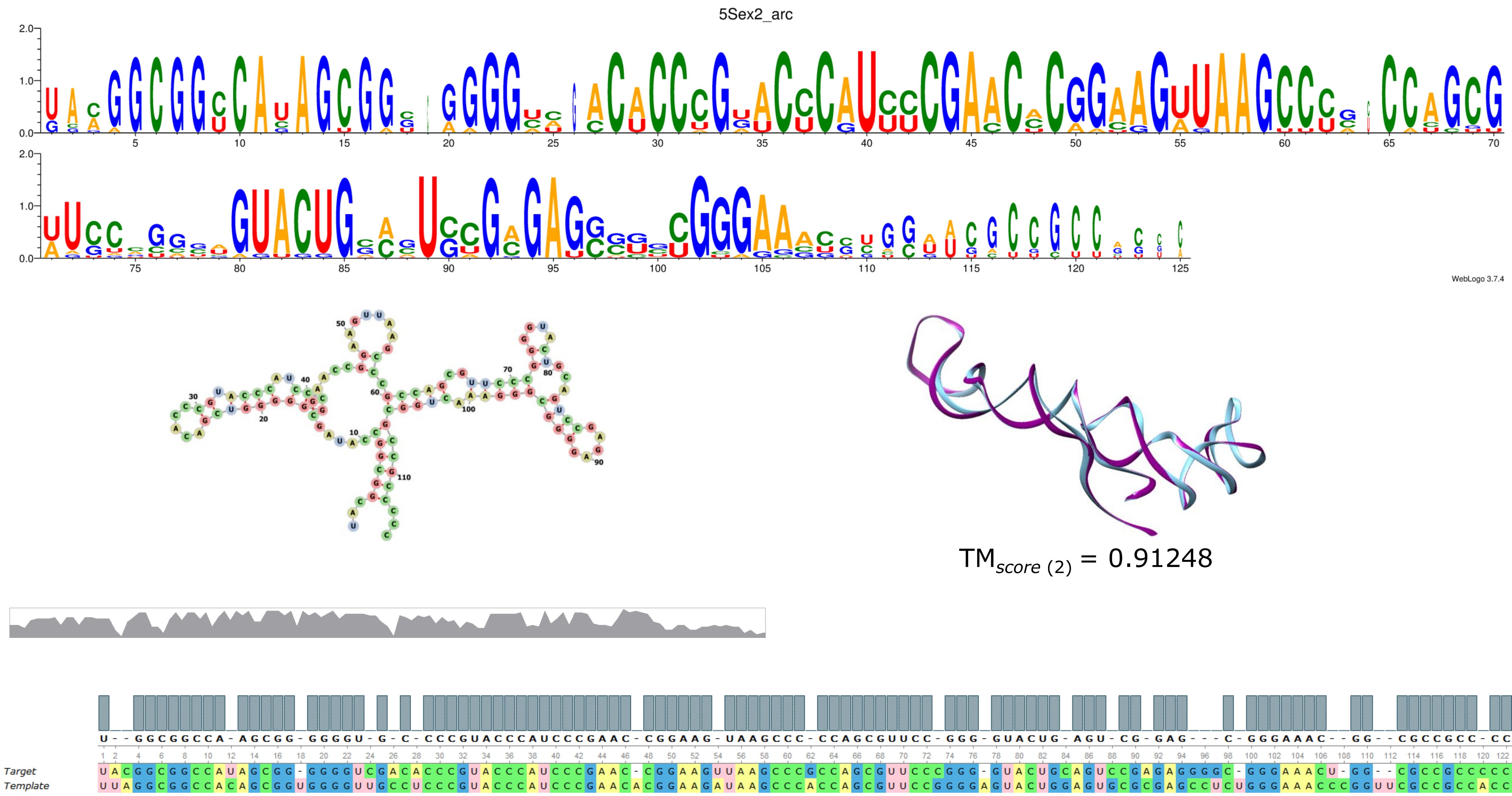

1B

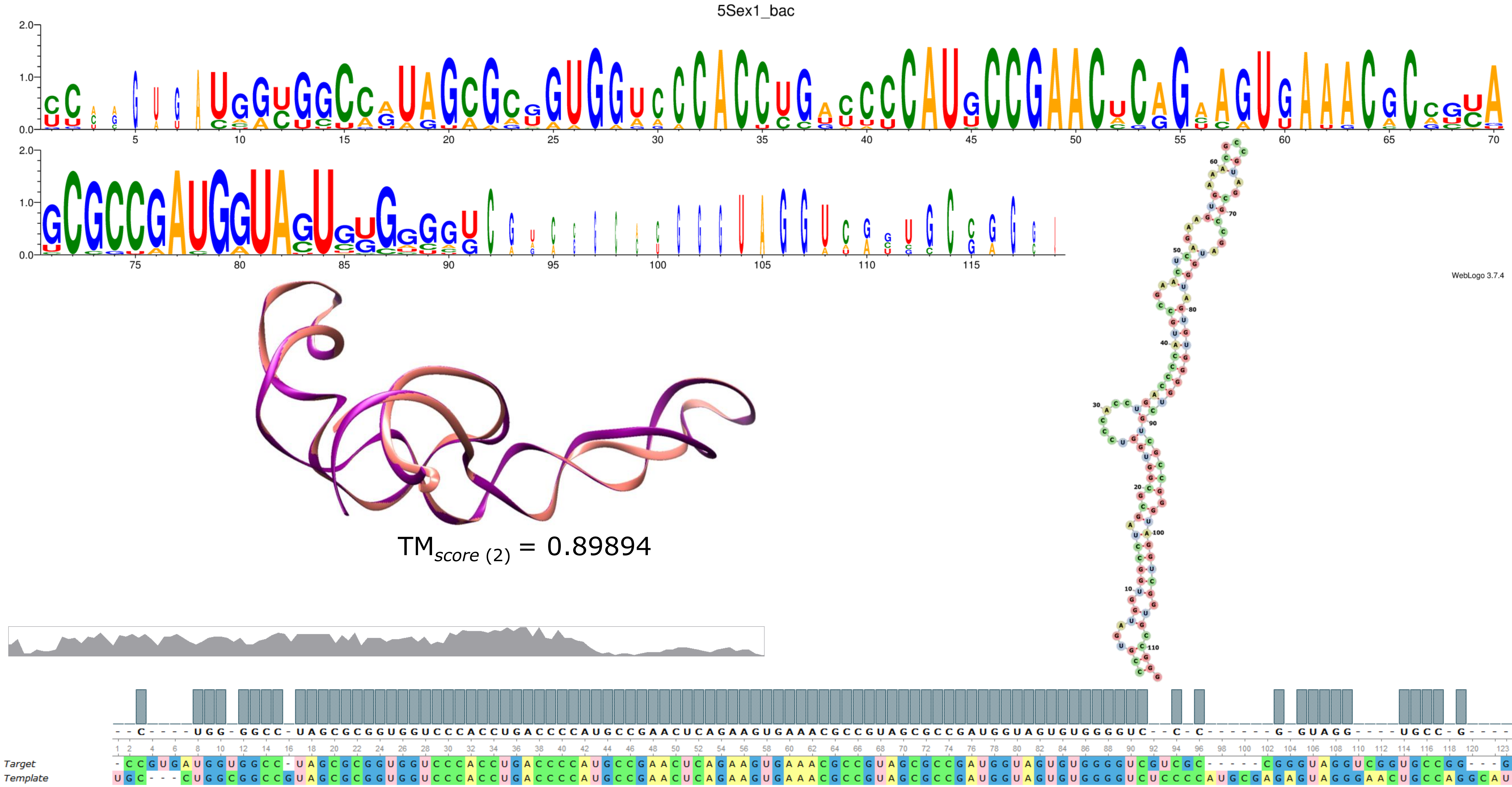

1C

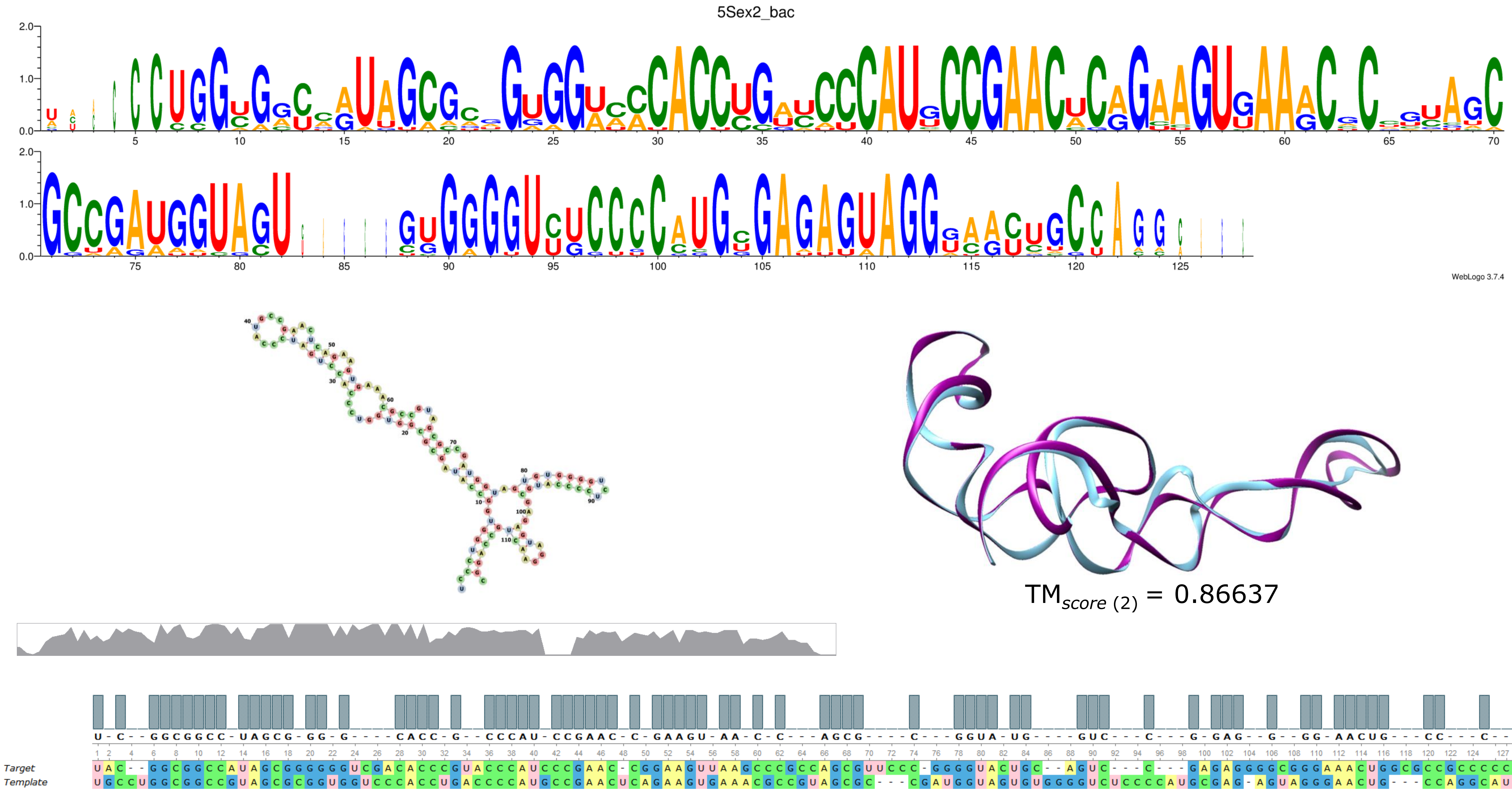

1D

	1 503									1 512
Arq1-Aciho_16S	-	-	-	-	-	-	-	-	-	-
Arq2-Nised_16S-c	A	U	C	A	C	C	U	-	-	-
Arq3-Hamar_16S-1c	-	-	-	-	-	-	-	-	-	-
Arq3-Hamar_16S-2	-	-	-	-	-	-	-	-	-	-
Arq3-Hamar_16S-3c	-	-	-	-	-	-	-	-	-	-
Arq4-Hxvol_16S-1y2	A	U	C	A	C	C	U	-	-	-
Arq5-Haqwa_16S-1	-	-	-	-	-	-	-	-	-	-
Arq5-Haqwa_16S-2	-	-	-	-	-	-	-	-	-	-
Arq6-Kocry_16S	A	U	C	A	C	C	-	-	-	-
Arq7-Mejan_16S-1c	A	U	C	A	C	C	U	-	-	-
Arq7-Mejan_16S-2	A	U	C	A	C	C	U	-	-	-
Arq8-Macet_16S-1	A	U	C	A	C	C	U	-	-	-
Arq8-Macet_16S-2	A	U	C	A	C	C	U	C	-	-
Arq8-Macet_16S-3	A	U	C	A	C	C	U	-	-	-
Arq9-Pyfur_16S	A	U	C	A	C	C	U	-	-	-
Arq10-Sacid_16S	A	U	C	A	C	-	-	-	-	-
Arq11-Thgam_16S-c	A	U	C	A	C	C	U	-	-	-
Arq12-Thsib_16S	A	G	G	C	U	C	C	G	U	G
Arq13-Tmvol_16S	-	-	-	-	-	-	-	-	-	-

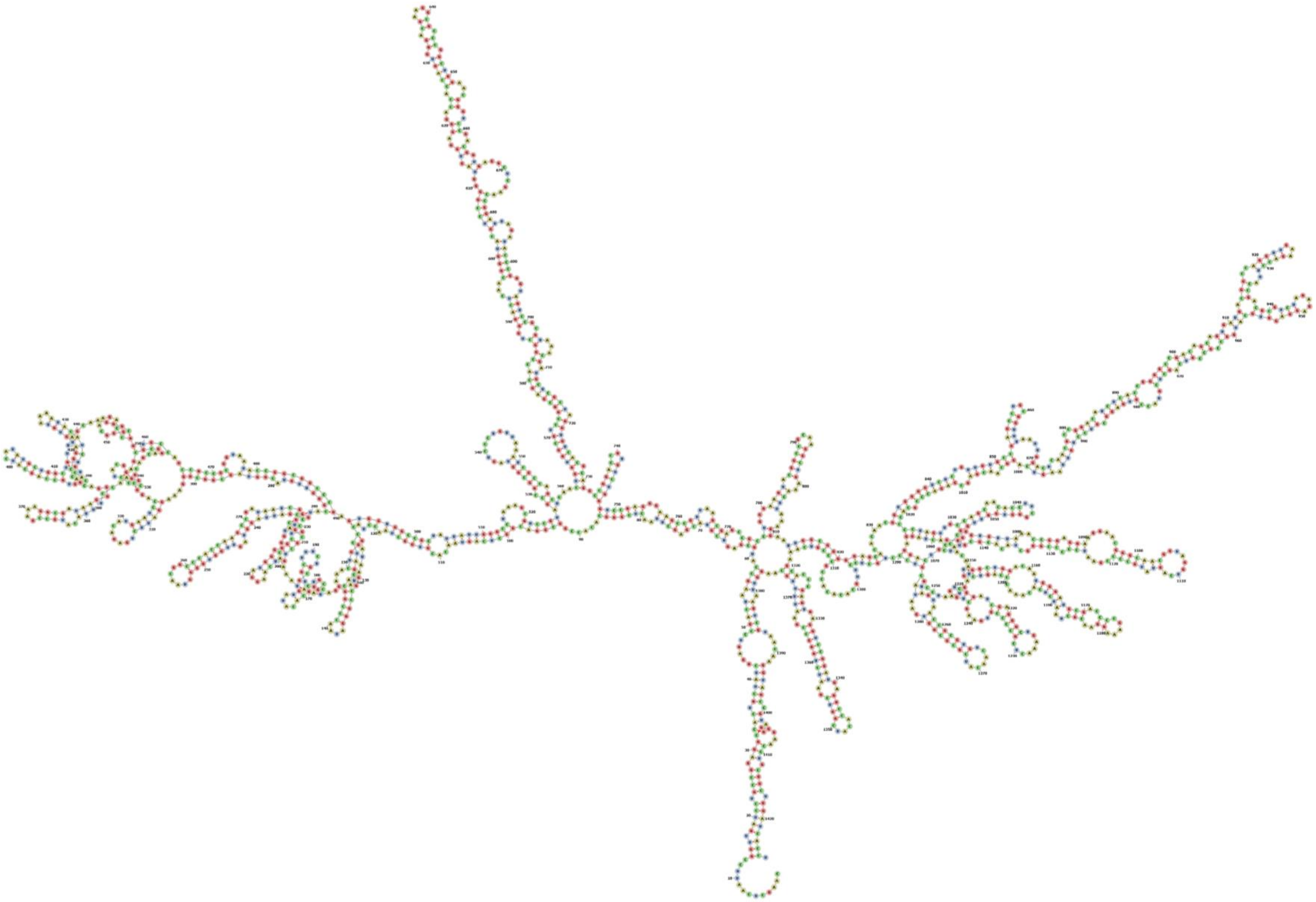

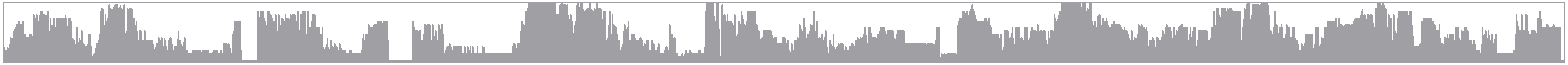

2A

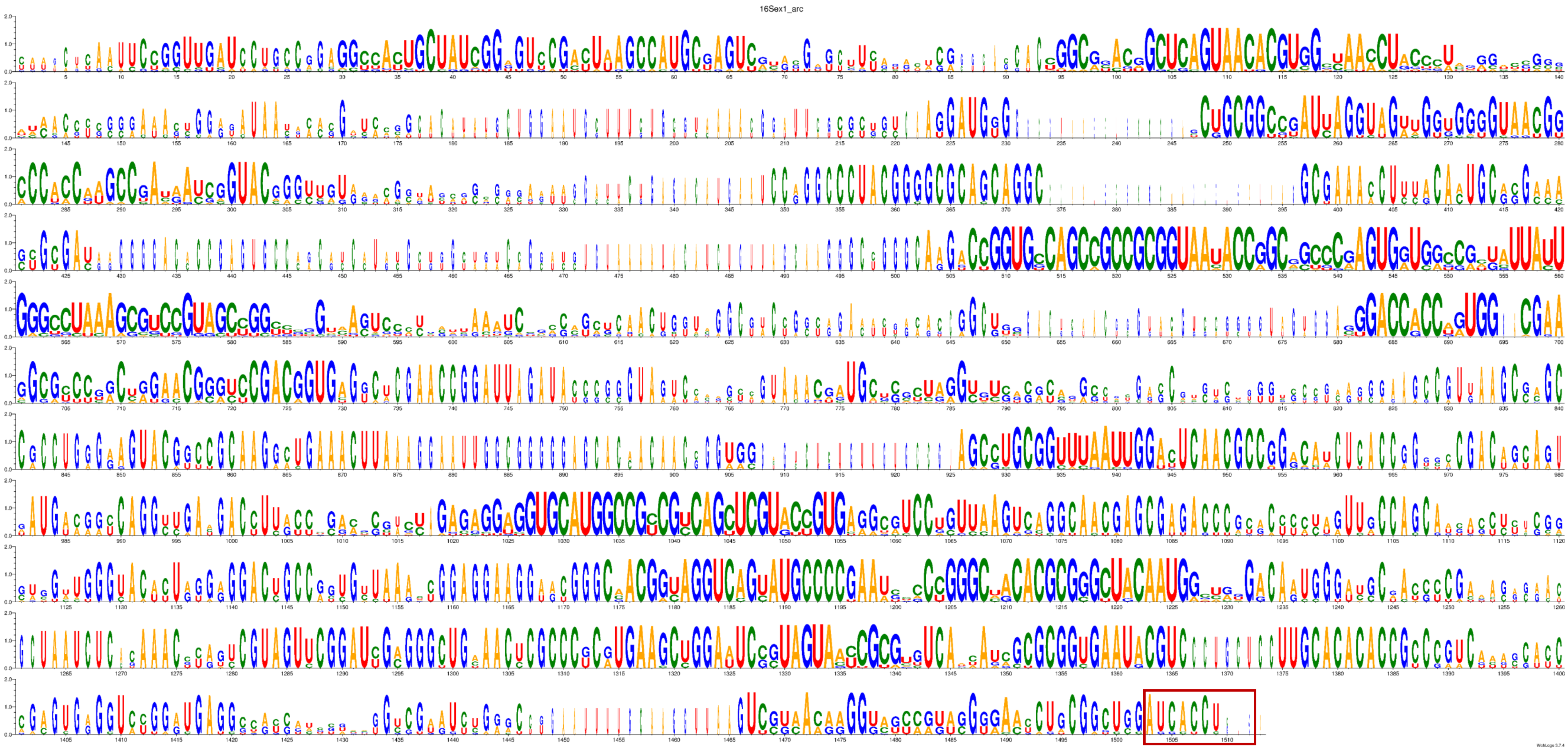

2A

	1 579						1585			1 588
Arq1-Aciho_16S	A	-	-	-	-	-	-	-	-	-
Arq2-Nised_16S-c	-	-	-	-	-	-	-	-	-	-
Arq3-Hamar_16S-1c	A	-	-	-	-	-	-	-	-	-
Arq3-Hamar_16S-2	A	-	-	-	-	-	-	-	-	-
Arq3-Hamar_16S-3-comp	A	U	C	A	C	C	U	C	C	U
Arq4-Hxvol_16S-1y2	-	-	-	-	-	-	-	-	-	-
Arq5-Haqwa_16S-1	A	U	C	A	C	C	U	C	C	U
Arq5-Haqwa_16S-2	A	U	C	A	C	C	U	C	C	U
Arq6-Kocry_16S	-	-	-	-	-	-	-	-	-	-
Arq7-Mejan_16S-1c	-	-	-	-	-	-	-	-	-	-
Arq7-Mejan_16S-2	A	-	-	-	-	-	-	-	-	-
Arq8-Macet_16S-1	A	-	-	-	-	-	-	-	-	-
Arq8-Macet_16S-2	A	U	-	-	-	-	-	-	-	-
Arq8-Macet_16S-3	A	-	-	-	-	-	-	-	-	-
Arq9-Pyfur_16S	A	U	C	A	C	C	U	C	C	U
Arq10-Sacid_16S	-	-	-	-	-	-	-	-	-	-
Arq11-Thgam_16S-c	A	U	C	A	C	C	U	C	C	U
Arq12-Thsib_16S	-	-	-	-	C	C	U	C	C	U
Arq13-Tmvol_16S	-	-	-	-	-	-	-	-	-	-

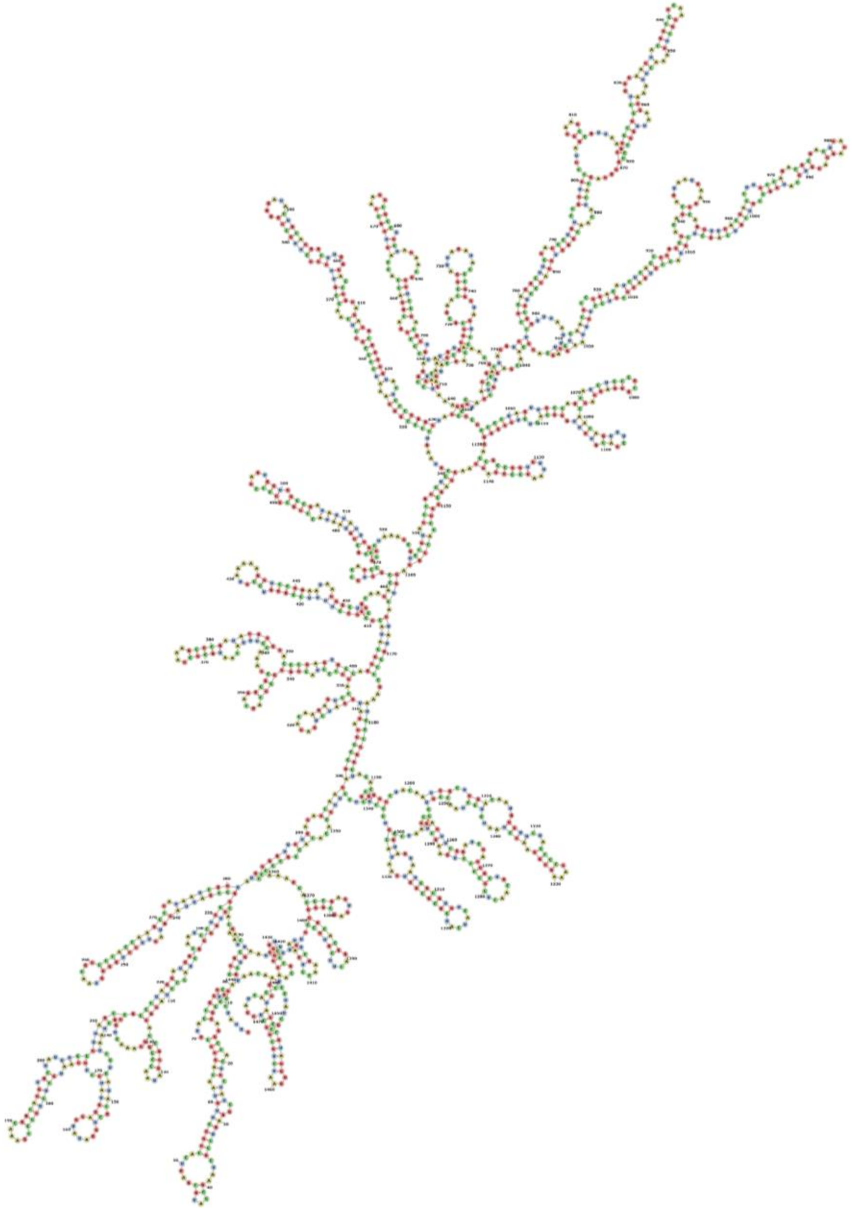

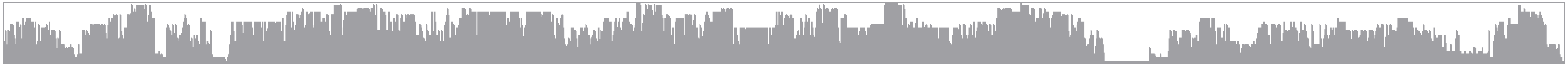

2B

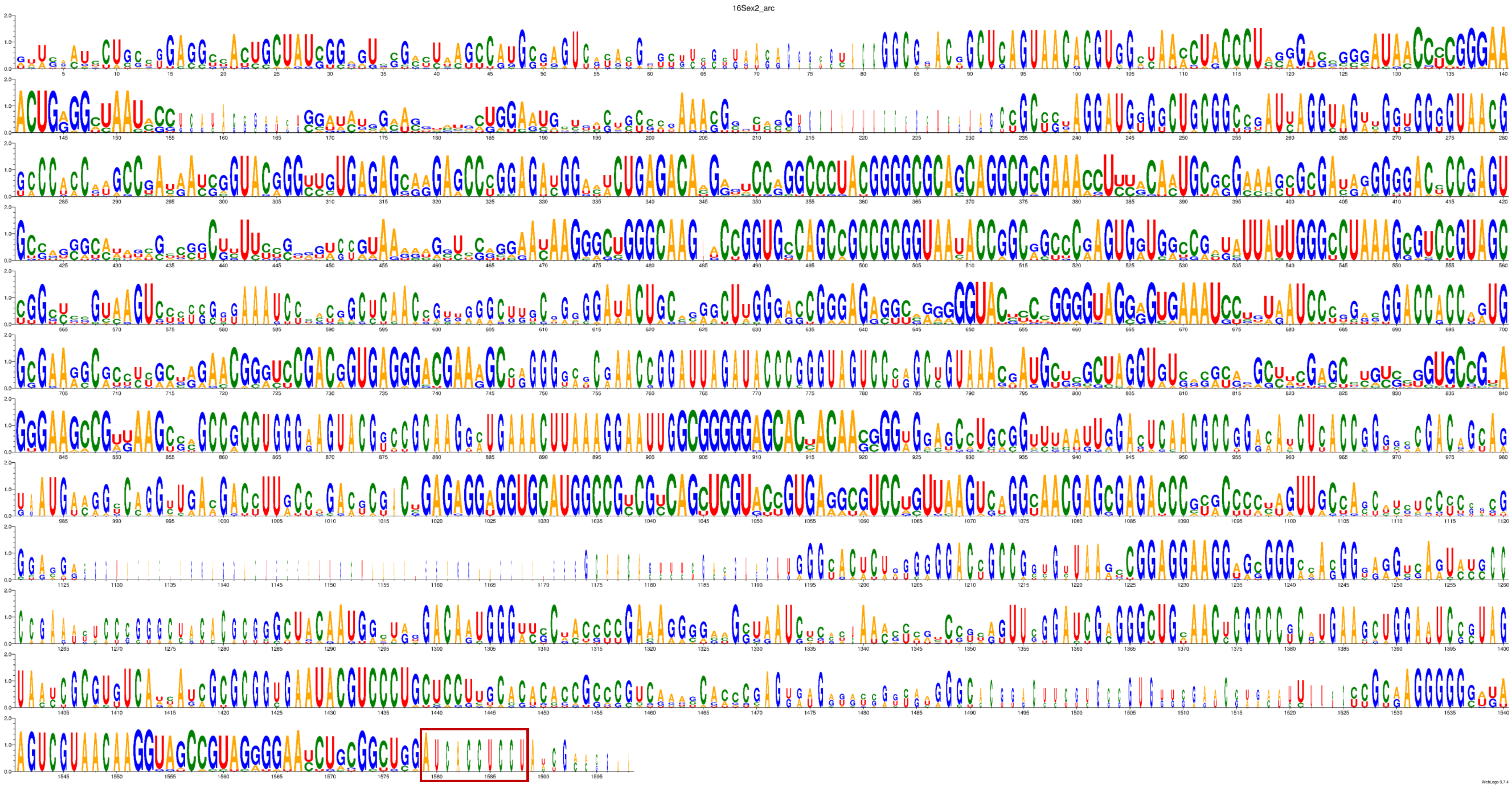

2B

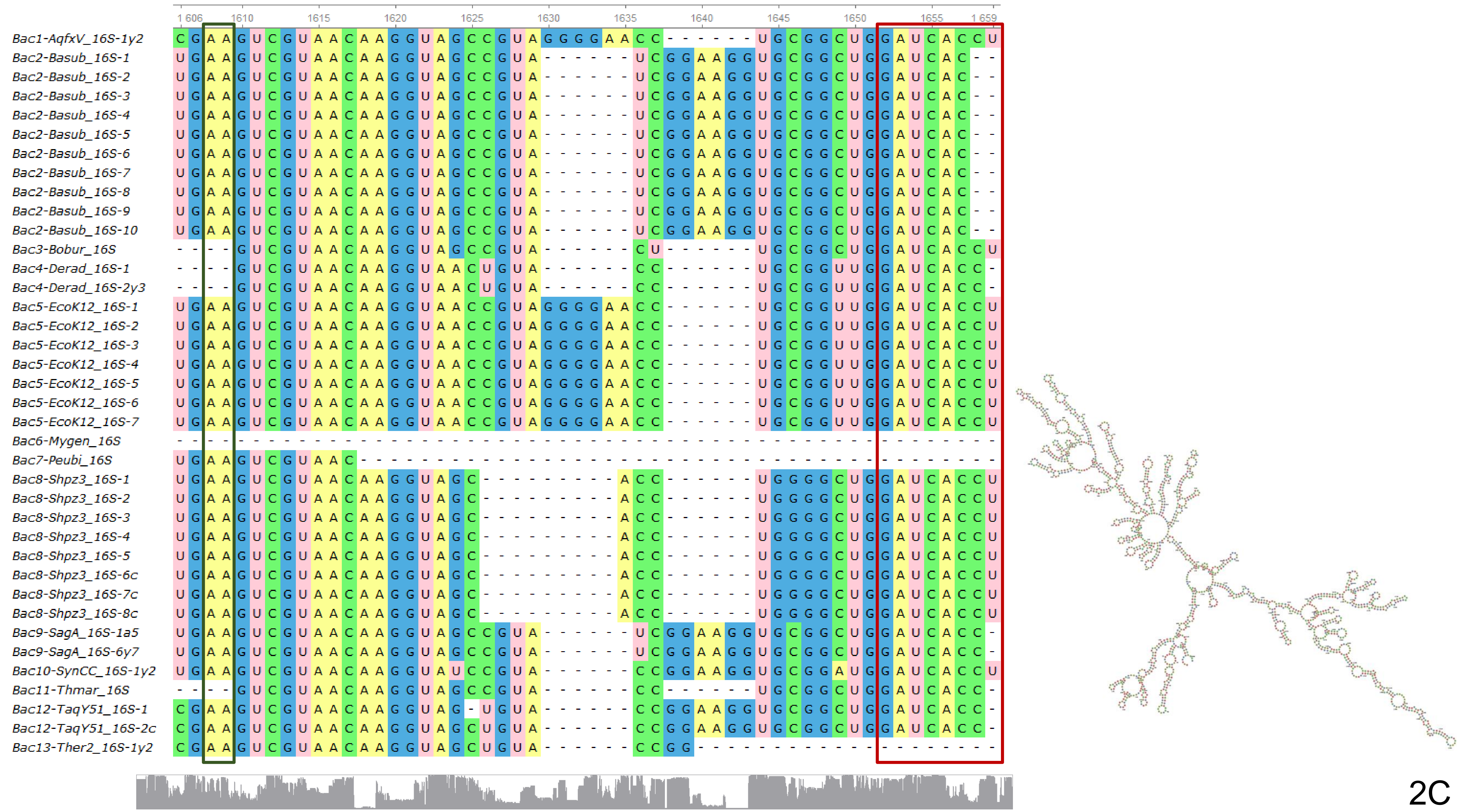
1 606
1610
1615
1620
1625
1630
1635
1640
1645
1650
1655
1 659
Bac1-AqfxV_16S-1y2
Bac2-Basub_16S-1
Bac2-Basub_16S-2
Bac2-Basub_16S-3
Bac2-Basub_16S-4
Bac2-Basub_16S-5
Bac2-Basub_16S-6
Bac2-Basub_16S-7
Bac2-Basub_16S-8
Bac2-Basub_16S-9
Bac2-Basub_16S-10
Bac3-Bobur_16S
Bac4-Derad_16S-1
Bac4-Derad_16S-2y3
Bac5-EcoK12_16S-1
Bac5-EcoK12_16S-2
Bac5-EcoK12_16S-3
Bac5-EcoK12_16S-4
Bac5-EcoK12_16S-5
Bac5-EcoK12_16S-6
Bac5-EcoK12_16S-7
Bac6-Mygen_16S
Bac7-Peubi_16S
Bac8-Shpz3_16S-1
Bac8-Shpz3_16S-2
Bac8-Shpz3_16S-3
Bac8-Shpz3_16S-4
Bac8-Shpz3_16S-5
Bac8-Shpz3_16S-6c
Bac8-Shpz3_16S-7c
Bac8-Shpz3_16S-8c
Bac9-SagA_16S-1a5
Bac9-SagA_16S-6y7
Bac10-SynCC_16S-1y2
Bac11-Thmar_16S
Bac12-TaqY51_16S-1
Bac12-TaqY51_16S-2c
Bac13-Ther2_16S-1y2

2C

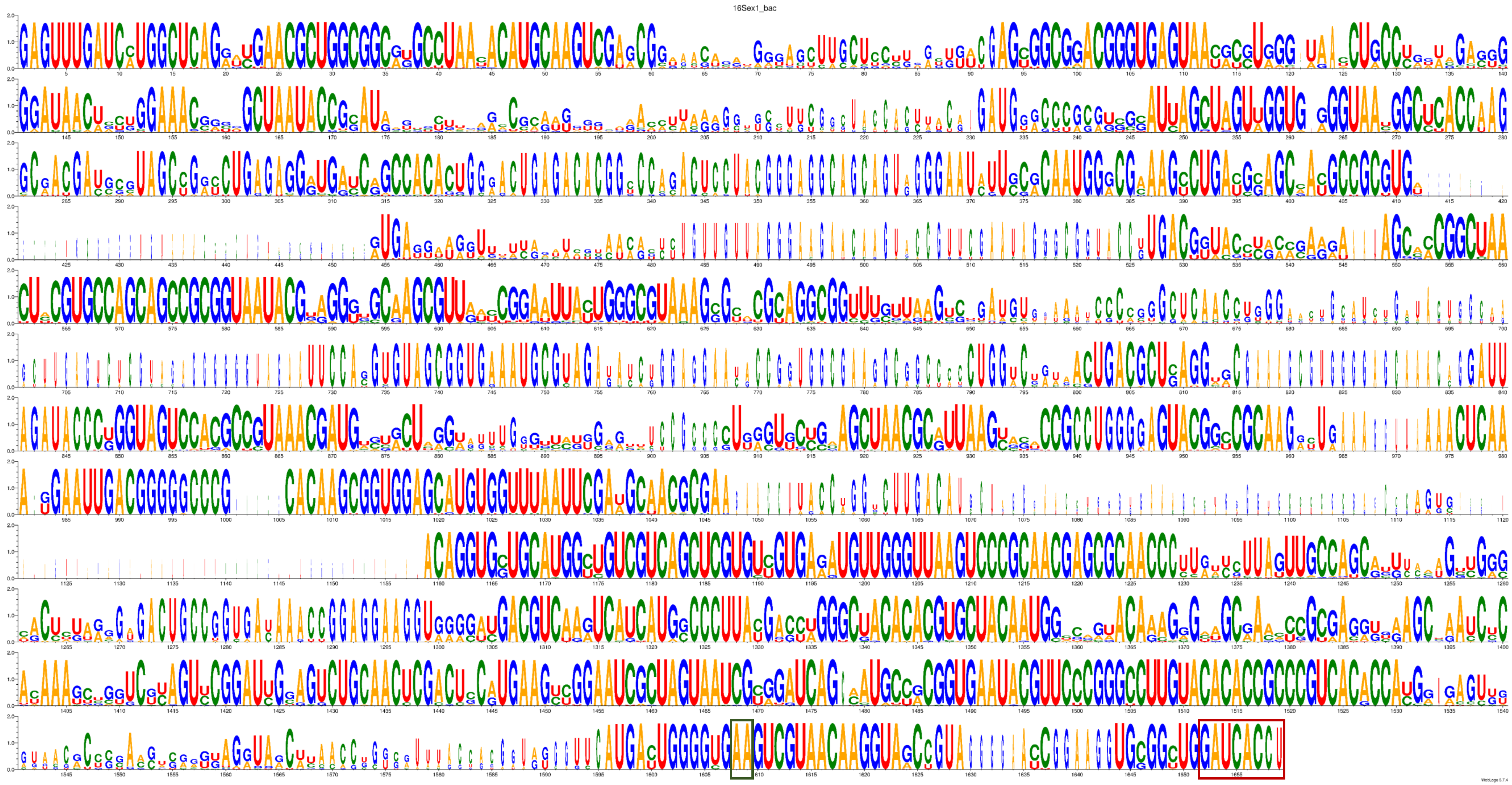

2C

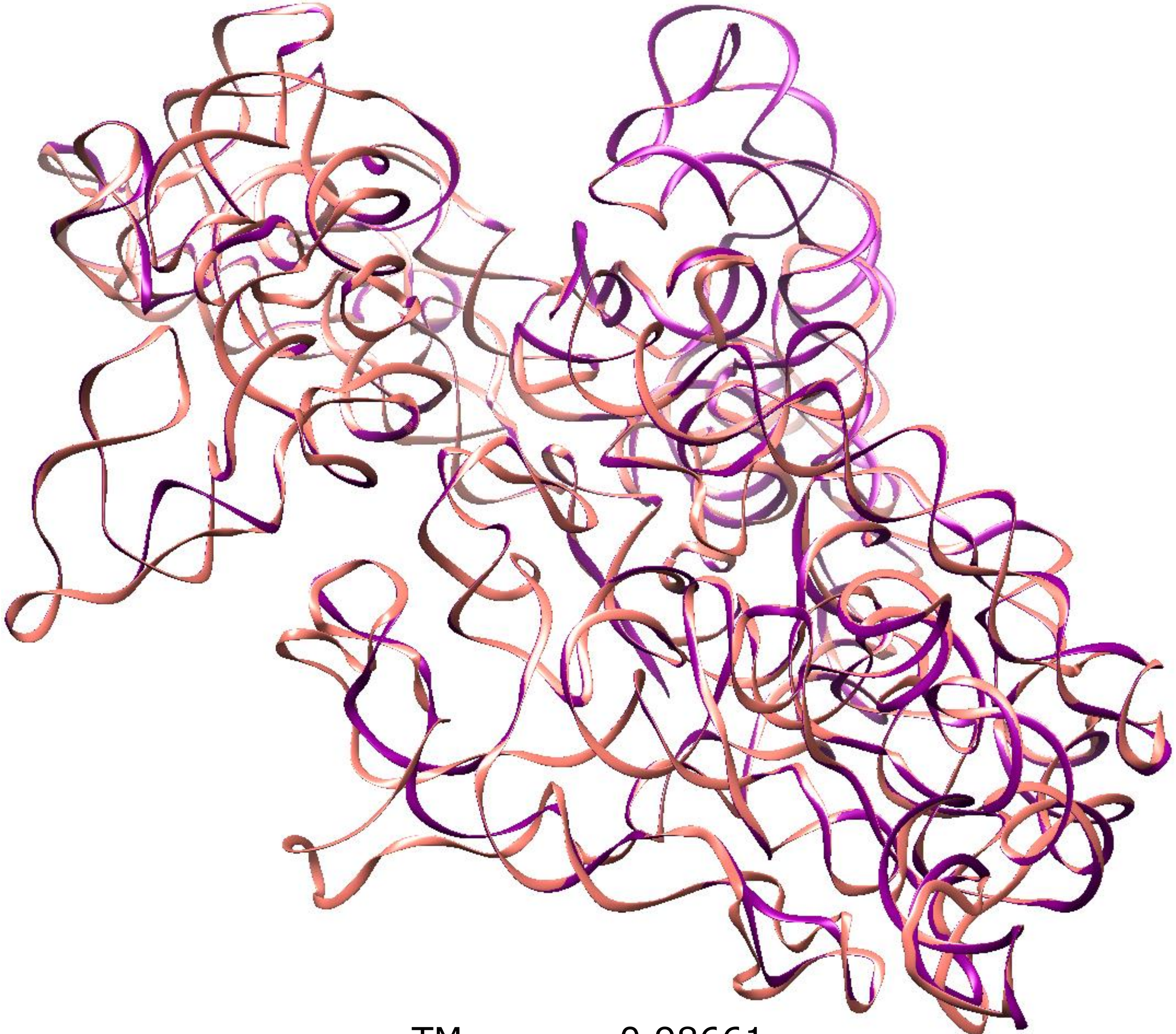

$TM_{score\ (2)} = 0.98661$

2C

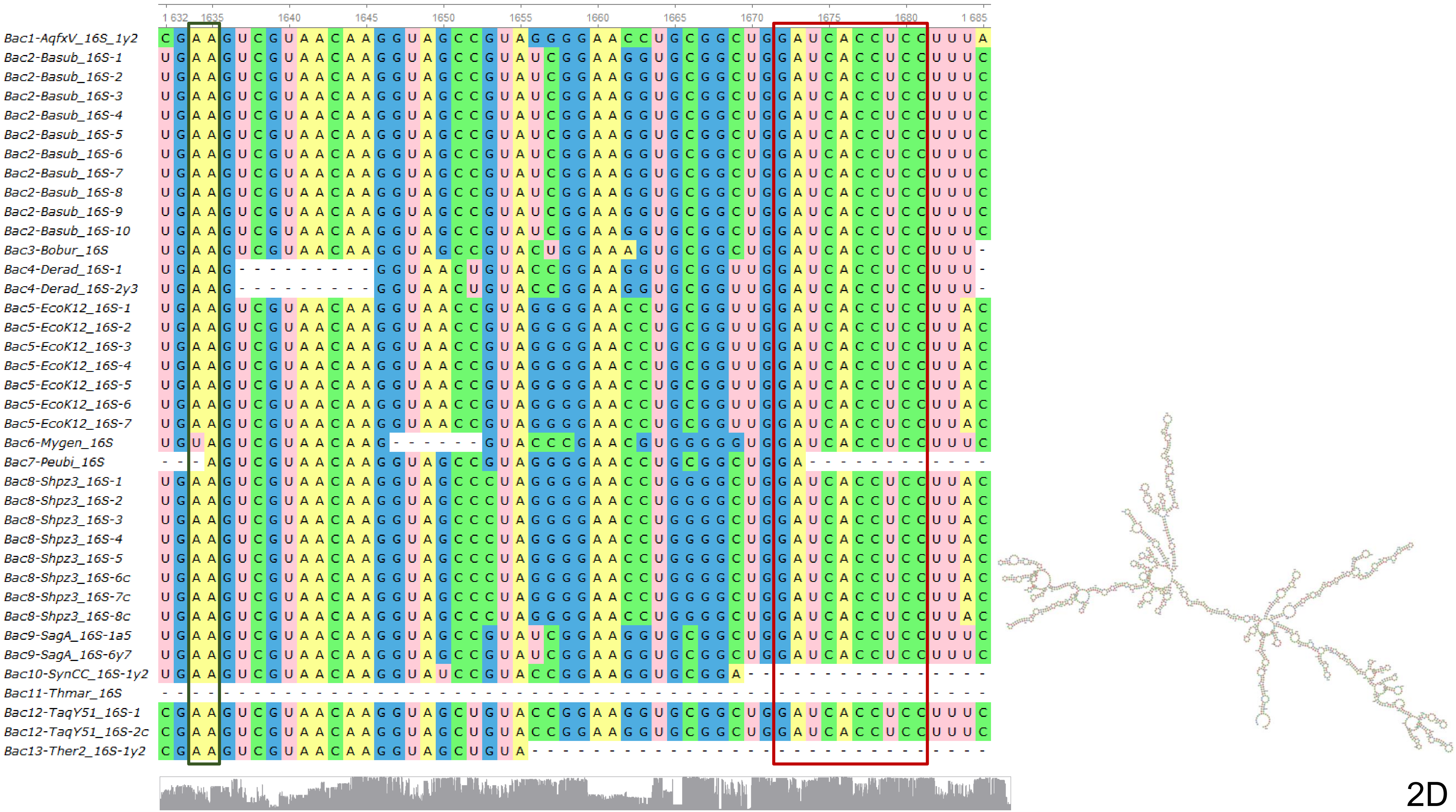

1 632 1635 1640 1645 1650 1655 1660 1665 1670 1675 1680 1 685
Bac1-AqfxV_16S_1y2 CGAAGUCGUAACAAGGUAGCCGUAGGGGAACCUGCGGCUGGAUCACCUCCUUUA
Bac2-Basub_16S-1 UGAAGUCGUAACAAGGUAGCCGUAUCGGAAGGUGCGGCUGGAUCACCUCCUUUC
Bac2-Basub_16S-2 UGAAGUCGUAACAAGGUAGCCGUAUCGGAAGGUGCGGCUGGAUCACCUCCUUUC
Bac2-Basub_16S-3 UGAAGUCGUAACAAGGUAGCCGUAUCGGAAGGUGCGGCUGGAUCACCUCCUUUC
Bac2-Basub_16S-4 UGAAGUCGUAACAAGGUAGCCGUAUCGGAAGGUGCGGCUGGAUCACCUCCUUUC
Bac2-Basub_16S-5 UGAAGUCGUAACAAGGUAGCCGUAUCGGAAGGUGCGGCUGGAUCACCUCCUUUC
Bac2-Basub_16S-6 UGAAGUCGUAACAAGGUAGCCGUAUCGGAAGGUGCGGCUGGAUCACCUCCUUUC
Bac2-Basub_16S-7 UGAAGUCGUAACAAGGUAGCCGUAUCGGAAGGUGCGGCUGGAUCACCUCCUUUC
Bac2-Basub_16S-8 UGAAGUCGUAACAAGGUAGCCGUAUCGGAAGGUGCGGCUGGAUCACCUCCUUUC
Bac2-Basub_16S-9 UGAAGUCGUAACAAGGUAGCCGUAUCGGAAGGUGCGGCUGGAUCACCUCCUUUC
Bac2-Basub_16S-10 UGAAGUCGUAACAAGGUAGCCGUAUCGGAAGGUGCGGCUGGAUCACCUCCUUUC
Bac3-Bobur_16S UGAAGUCGUAACAAGGUAGCCGUACUGGAAAGUGCGGCUGGAUCACCUCCUUU-
Bac4-Derad_16S-1 UGAAG---------GGUAACUGUACCGGAAGGUGCGGUUGGAUCACCUCCUUU-
Bac4-Derad_16S-2y3 UGAAG---------GGUAACUGUACCGGAAGGUGCGGUUGGAUCACCUCCUUU-
Bac5-EcoK12_16S-1 UGAAGUCGUAACAAGGUAACCGUAGGGGAACCUGCGGUUGGAUCACCUCCUUAC
Bac5-EcoK12_16S-2 UGAAGUCGUAACAAGGUAACCGUAGGGGAACCUGCGGUUGGAUCACCUCCUUAC
Bac5-EcoK12_16S-3 UGAAGUCGUAACAAGGUAACCGUAGGGGAACCUGCGGUUGGAUCACCUCCUUAC
Bac5-EcoK12_16S-4 UGAAGUCGUAACAAGGUAACCGUAGGGGAACCUGCGGUUGGAUCACCUCCUUAC
Bac5-EcoK12_16S-5 UGAAGUCGUAACAAGGUAACCGUAGGGGAACCUGCGGUUGGAUCACCUCCUUAC
Bac5-EcoK12_16S-6 UGAAGUCGUAACAAGGUAACCGUAGGGGAACCUGCGGUUGGAUCACCUCCUUAC
Bac5-EcoK12_16S-7 UGAAGUCGUAACAAGGUAACCGUAGGGGAACCUGCGGUUGGAUCACCUCCUUAC
Bac6-Mygen_16S UGUAGUCGUAACAAG------GUACCCGAACGUGGGGGUGGAUCACCUCCUUUC
Bac7-Peubi_16S ---AGUCGUAACAAGGUAGCCGUAGGGGAACCUGCGGCUGGA------------
Bac8-Shpz3_16S-1 UGAAGUCGUAACAAGGUAGCCCUAGGGGAACCUGGGGCUGGAUCACCUCCUUAC
Bac8-Shpz3_16S-2 UGAAGUCGUAACAAGGUAGCCCUAGGGGAACCUGGGGCUGGAUCACCUCCUUAC
Bac8-Shpz3_16S-3 UGAAGUCGUAACAAGGUAGCCCUAGGGGAACCUGGGGCUGGAUCACCUCCUUAC
Bac8-Shpz3_16S-4 UGAAGUCGUAACAAGGUAGCCCUAGGGGAACCUGGGGCUGGAUCACCUCCUUAC
Bac8-Shpz3_16S-5 UGAAGUCGUAACAAGGUAGCCCUAGGGGAACCUGGGGCUGGAUCACCUCCUUAC
Bac8-Shpz3_16S-6c UGAAGUCGUAACAAGGUAGCCCUAGGGGAACCUGGGGCUGGAUCACCUCCUUAC
Bac8-Shpz3_16S-7c UGAAGUCGUAACAAGGUAGCCCUAGGGGAACCUGGGGCUGGAUCACCUCCUUAC
Bac8-Shpz3_16S-8c UGAAGUCGUAACAAGGUAGCCCUAGGGGAACCUGGGGCUGGAUCACCUCCUUAC
Bac9-SagA_16S-1a5 UGAAGUCGUAACAAGGUAGCCGUAUCGGAAGGUGCGGCUGGAUCACCUCCUUUC
Bac9-SagA_16S-6y7 UGAAGUCGUAACAAGGUAGCCGUAUCGGAAGGUGCGGCUGGAUCACCUCCUUUC
Bac10-SynCC_16S-1y2 UGAAGUCGUAACAAGGUAUCCGUACCGGAAGGUGCGGA----------------
Bac11-Thmar_16S --
Bac12-TaqY51_16S-1 CGAAGUCGUAACAAGGUAGCUGUACCGGAAGGUGCGGCUGGAUCACCUCCUUUC
Bac12-TaqY51_16S-2c CGAAGUCGUAACAAGGUAGCUGUACCGGAAGGUGCGGCUGGAUCACCUCCUUUC
Bac13-Ther2_16S-1y2 CGAAGUCGUAACAAGGUAGCUGUA------------------------------

2D

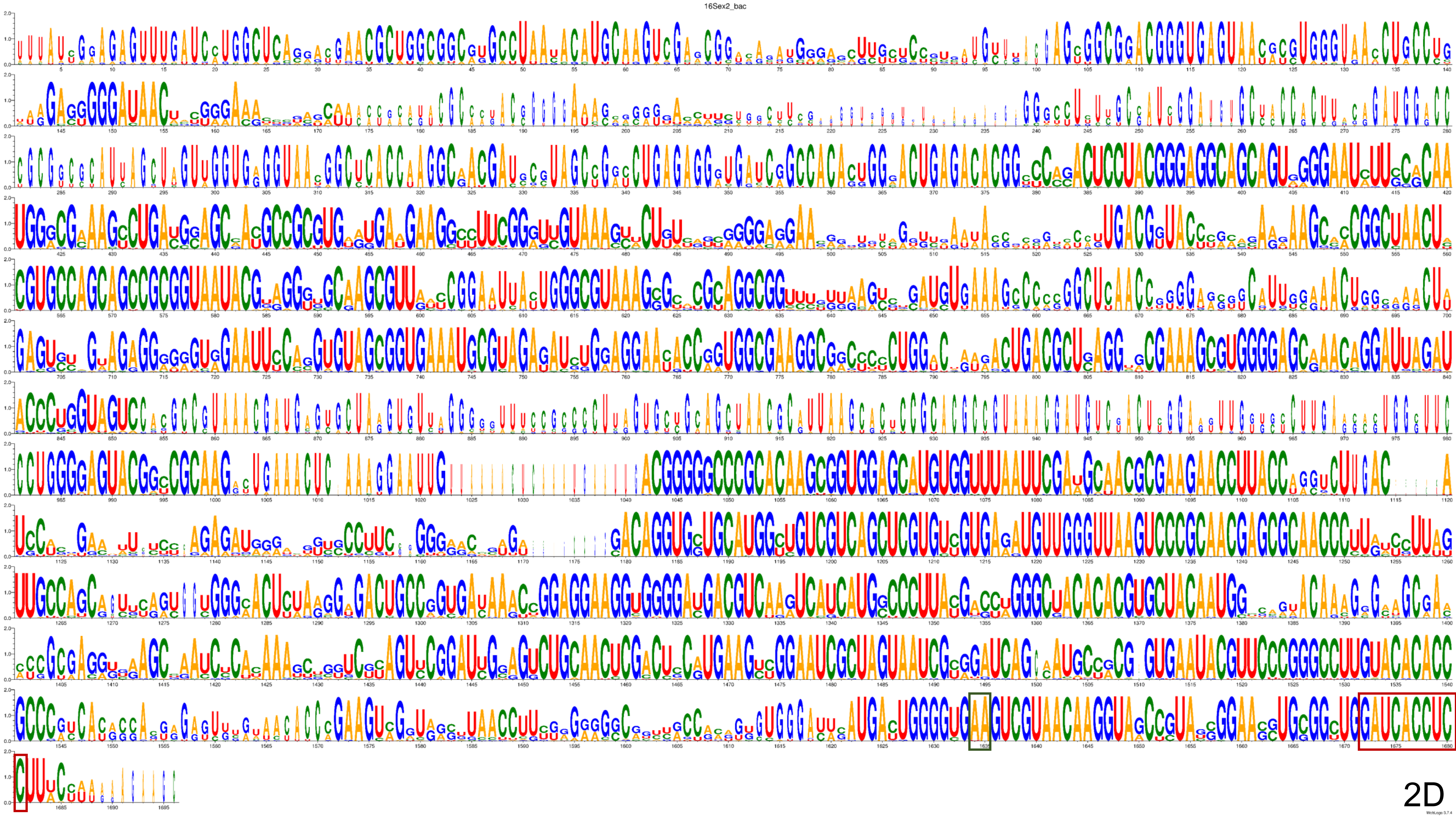

2D

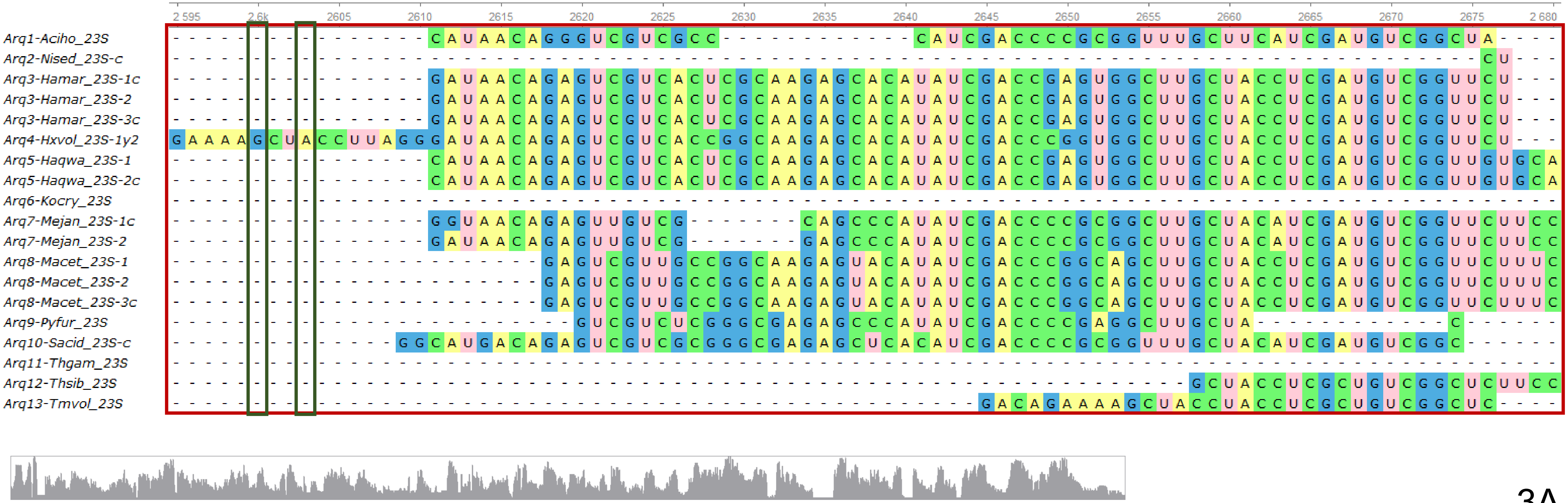
2 595
2.6k
2605
2610
2615
2620
2625
2630
2635
2640
2645
2650
2655
2660
2665
2670
2675
2 680
Arq1-Aciho_23S
Arq2-Nised_23S-c
Arq3-Hamar_23S-1c
Arq3-Hamar_23S-2
Arq3-Hamar_23S-3c
Arq4-Hxvol_23S-1y2
Arq5-Haqwa_23S-1
Arq5-Haqwa_23S-2c
Arq6-Kocry_23S
Arq7-Mejan_23S-1c
Arq7-Mejan_23S-2
Arq8-Macet_23S-1
Arq8-Macet_23S-2
Arq8-Macet_23S-3c
Arq9-Pyfur_23S
Arq10-Sacid_23S-c
Arq11-Thgam_23S
Arq12-Thsib_23S
Arq13-Tmvol_23S

3A

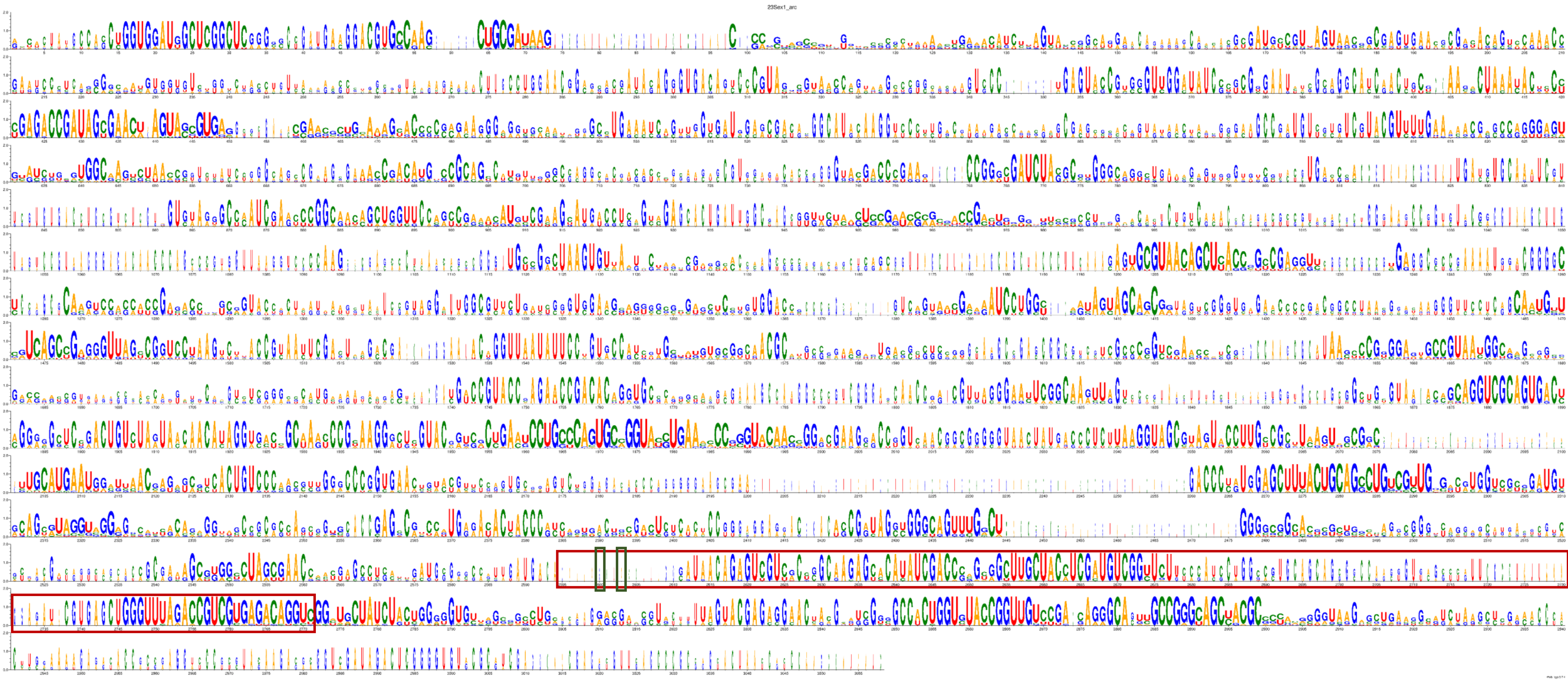

3A

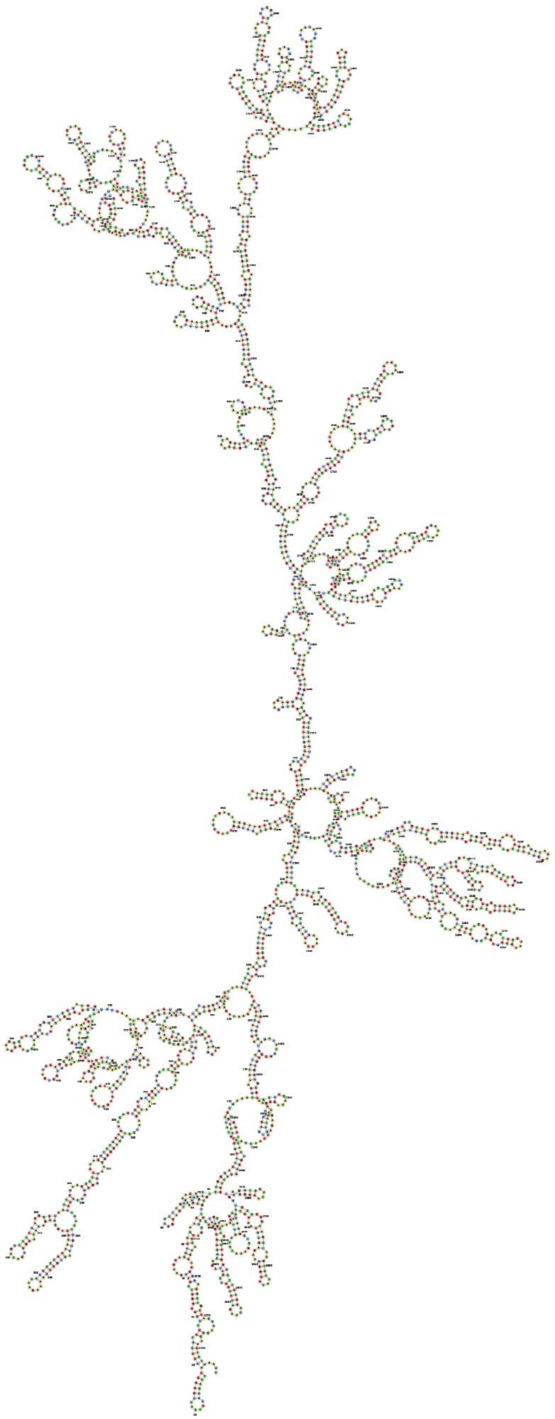

3A

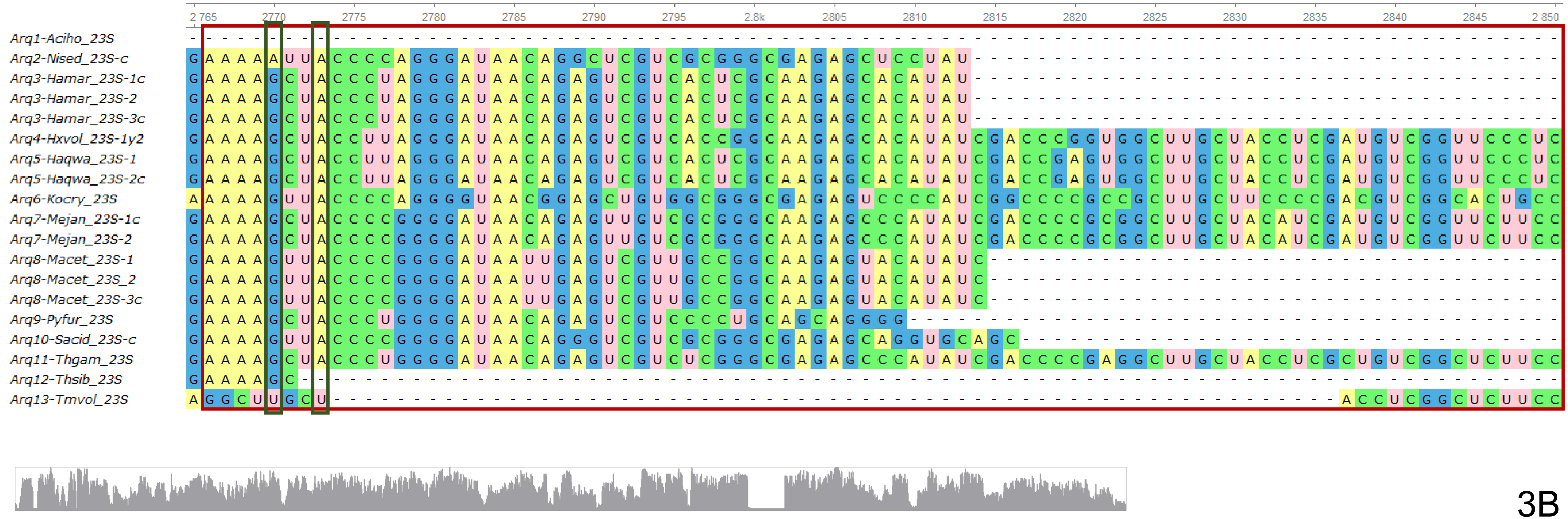
2 765
2770
2775
2780
2785
2790
2795
2.8k
2805
2810
2815
2820
2825
2830
2835
2840
2845
2 850
Arq1-Aciho_23S
Arq2-Nised_23S-c
Arq3-Hamar_23S-1c
Arq3-Hamar_23S-2
Arq3-Hamar_23S-3c
Arq4-Hxvol_23S-1y2
Arq5-Haqwa_23S-1
Arq5-Haqwa_23S-2c
Arq6-Kocry_23S
Arq7-Mejan_23S-1c
Arq7-Mejan_23S-2
Arq8-Macet_23S-1
Arq8-Macet_23S_2
Arq8-Macet_23S-3c
Arq9-Pyfur_23S
Arq10-Sacid_23S-c
Arq11-Thgam_23S
Arq12-Thsib_23S
Arq13-Tmvol_23S

3B

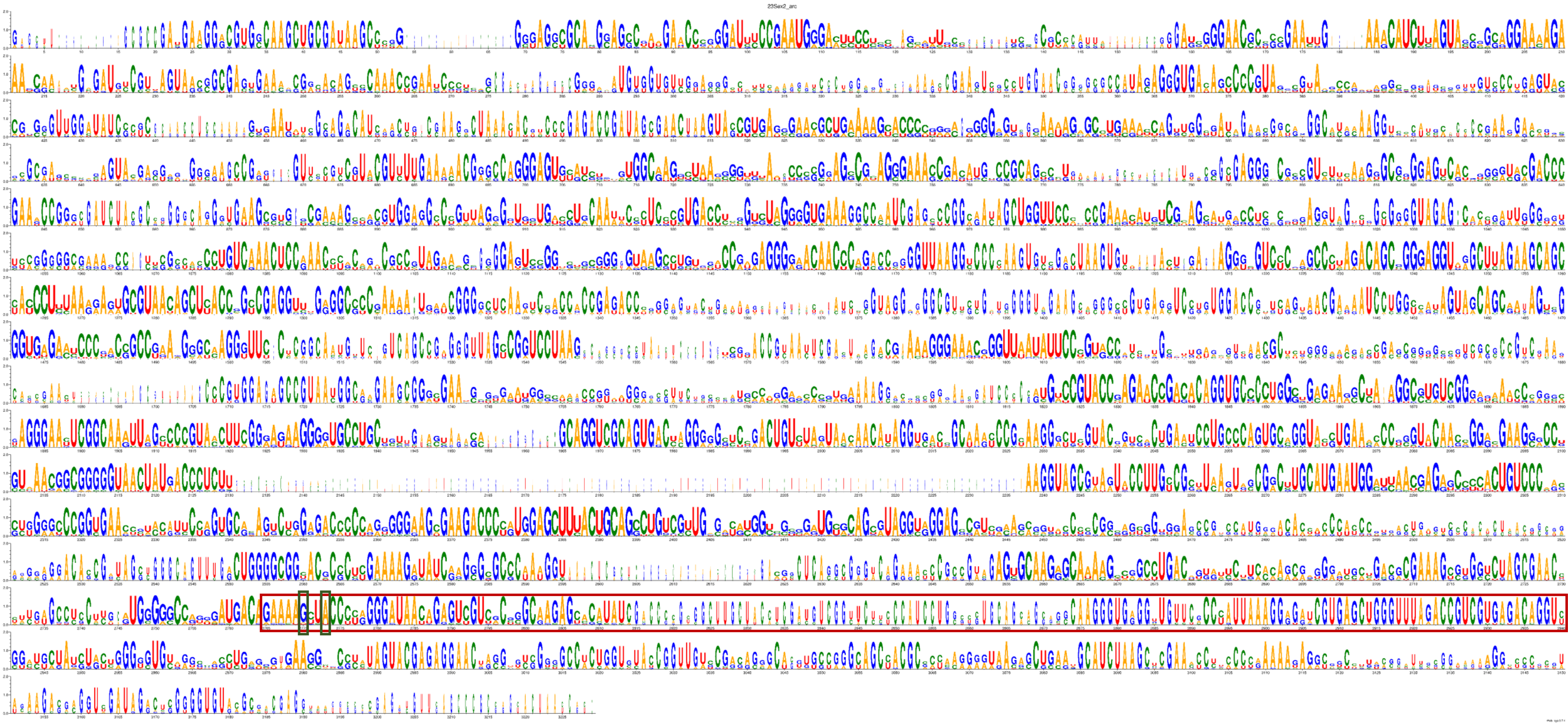

3B

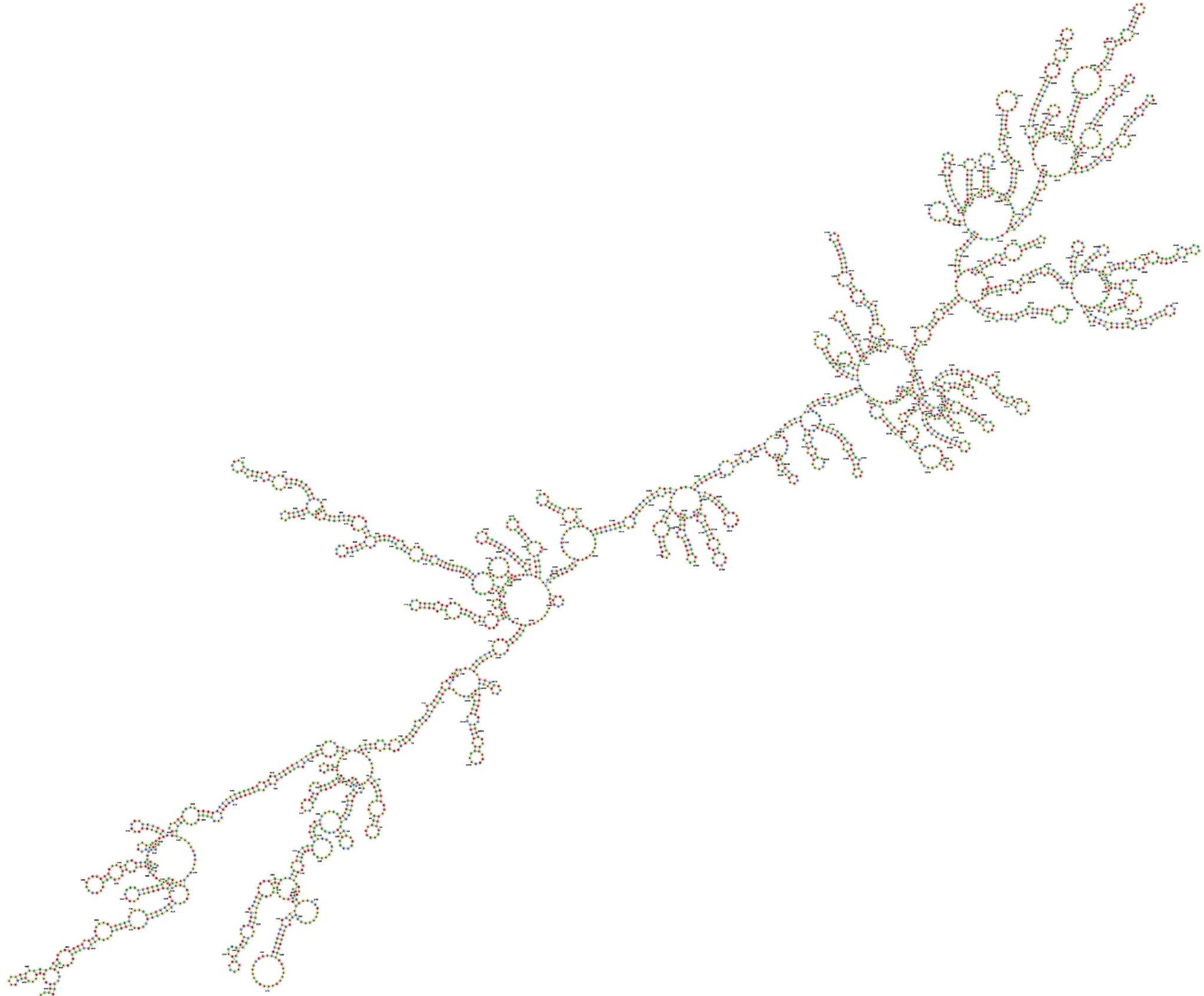

3B

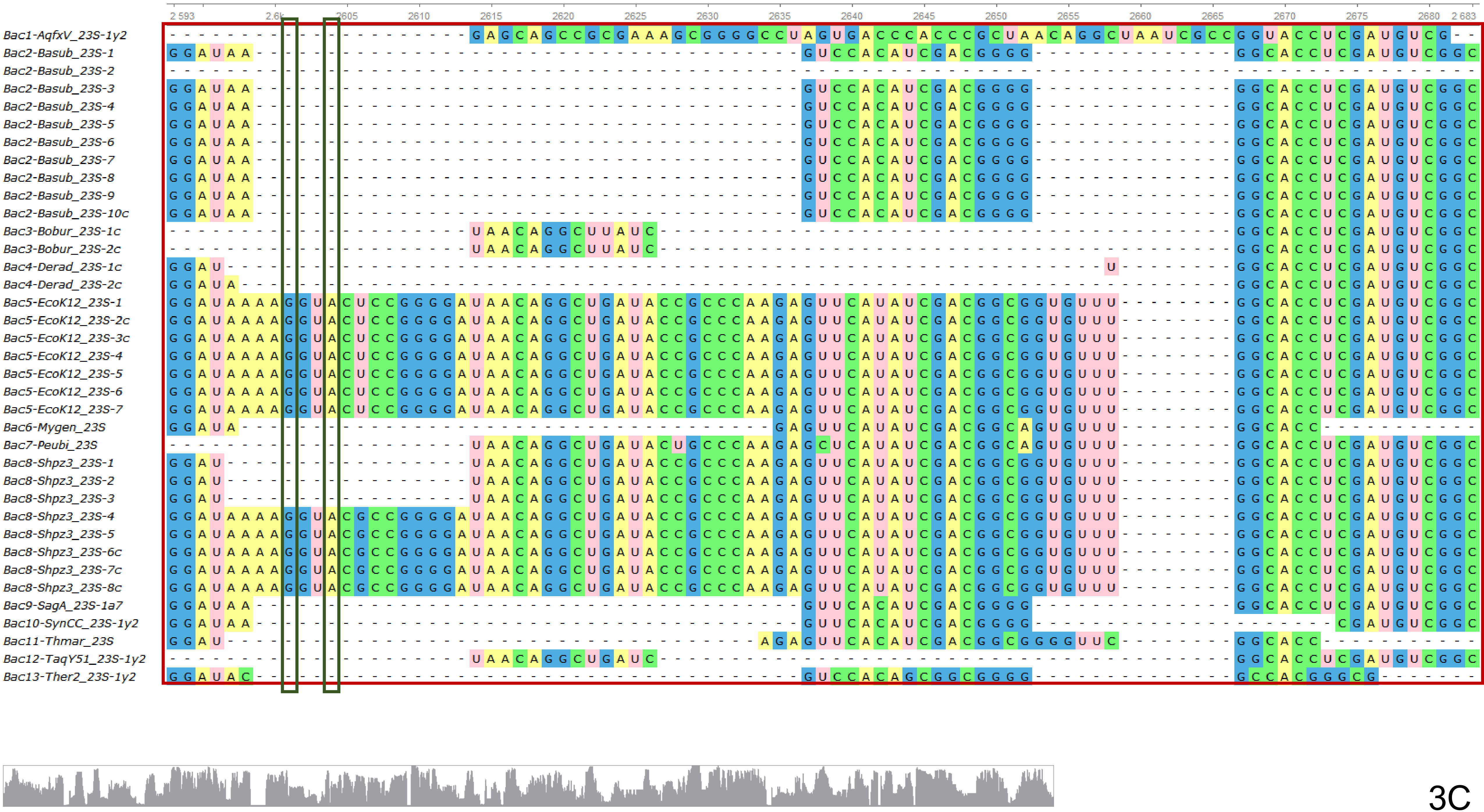
2 593
2.6k
2605
2610
2615
2620
2625
2630
2635
2640
2645
2650
2655
2660
2665
2670
2675
2680
2 683
Bac1-AqfxV_23S-1y2
Bac2-Basub_23S-1
Bac2-Basub_23S-2
Bac2-Basub_23S-3
Bac2-Basub_23S-4
Bac2-Basub_23S-5
Bac2-Basub_23S-6
Bac2-Basub_23S-7
Bac2-Basub_23S-8
Bac2-Basub_23S-9
Bac2-Basub_23S-10c
Bac3-Bobur_23S-1c
Bac3-Bobur_23S-2c
Bac4-Derad_23S-1c
Bac4-Derad_23S-2c
Bac5-EcoK12_23S-1
Bac5-EcoK12_23S-2c
Bac5-EcoK12_23S-3c
Bac5-EcoK12_23S-4
Bac5-EcoK12_23S-5
Bac5-EcoK12_23S-6
Bac5-EcoK12_23S-7
Bac6-Mygen_23S
Bac7-Peubi_23S
Bac8-Shpz3_23S-1
Bac8-Shpz3_23S-2
Bac8-Shpz3_23S-3
Bac8-Shpz3_23S-4
Bac8-Shpz3_23S-5
Bac8-Shpz3_23S-6c
Bac8-Shpz3_23S-7c
Bac8-Shpz3_23S-8c
Bac9-SagA_23S-1a7
Bac10-SynCC_23S-1y2
Bac11-Thmar_23S
Bac12-TaqY51_23S-1y2
Bac13-Ther2_23S-1y2

3C

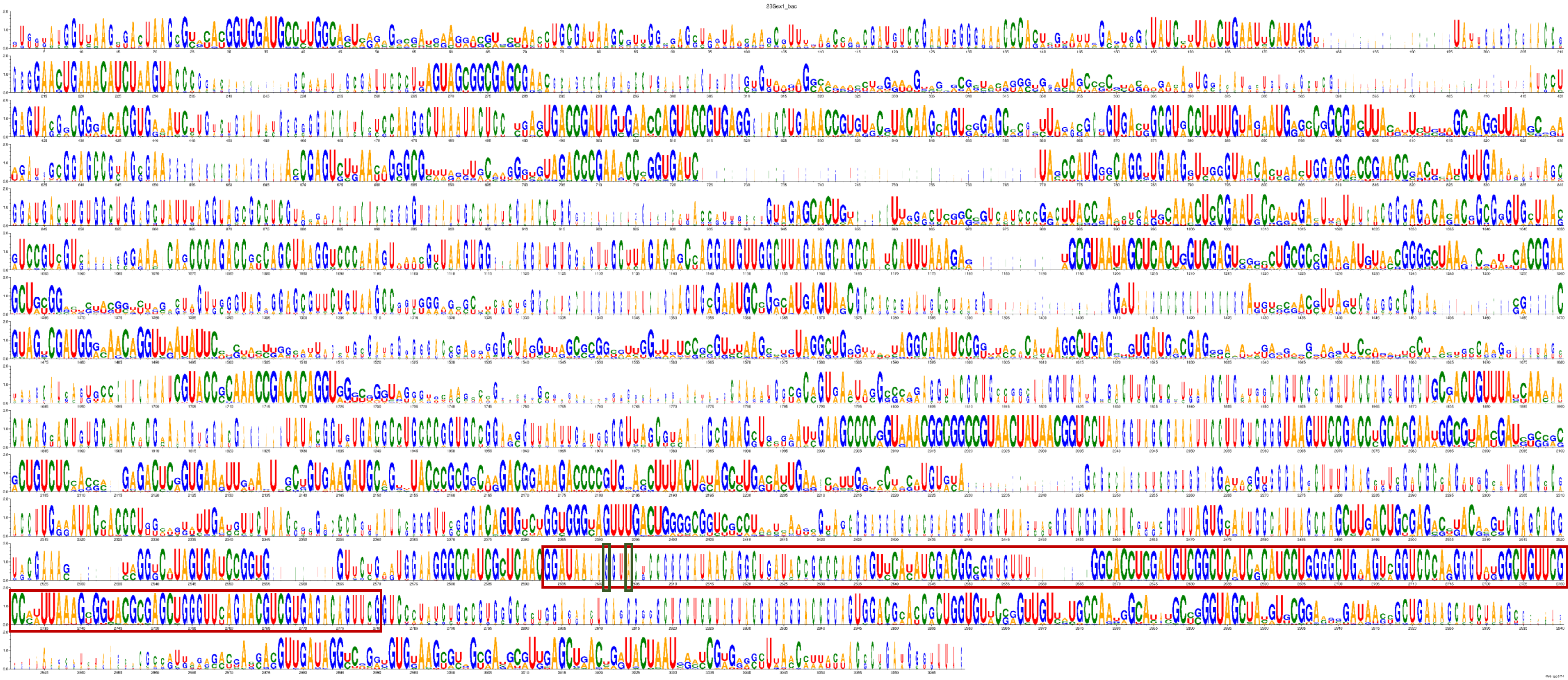

3C

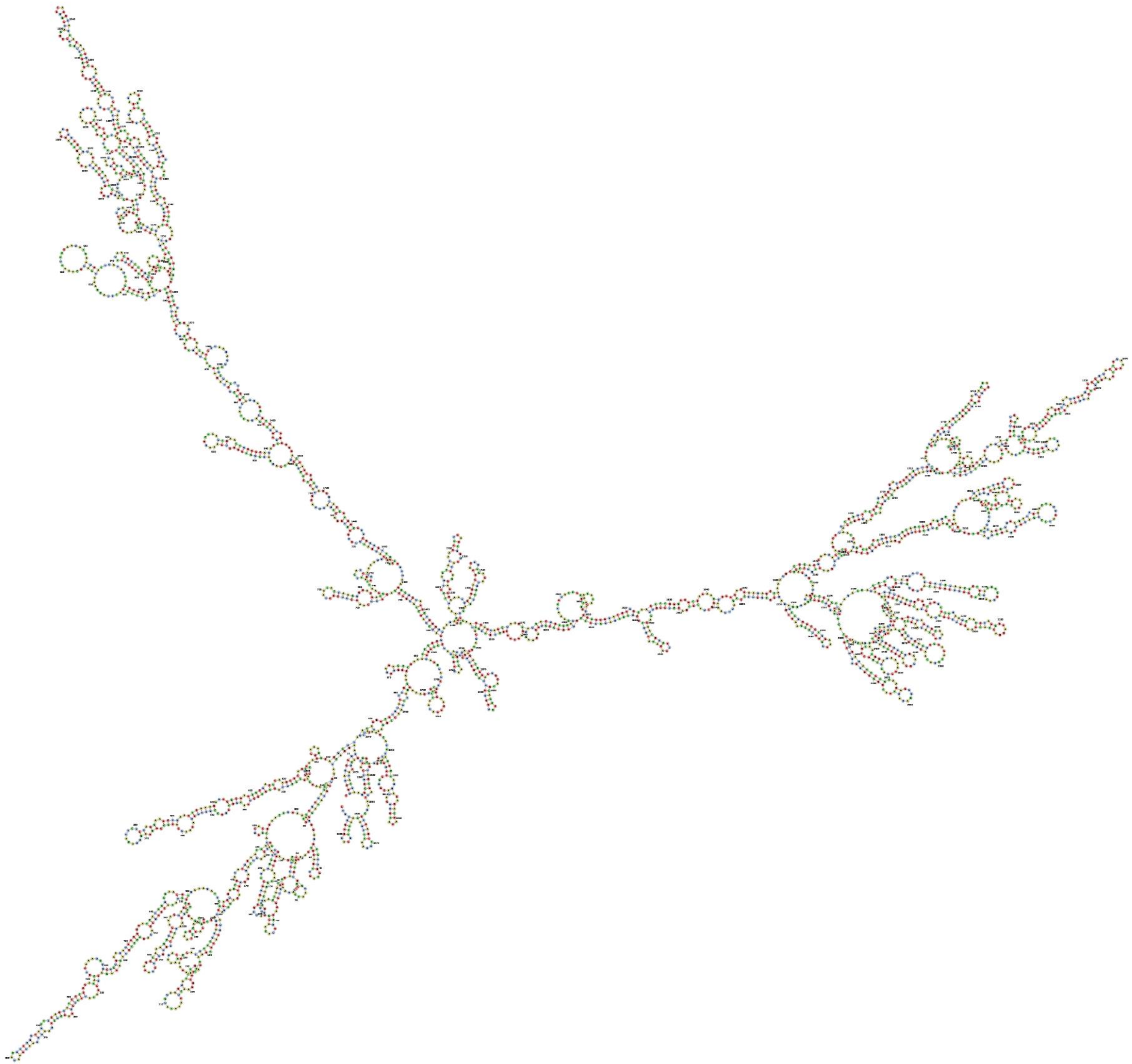

3C

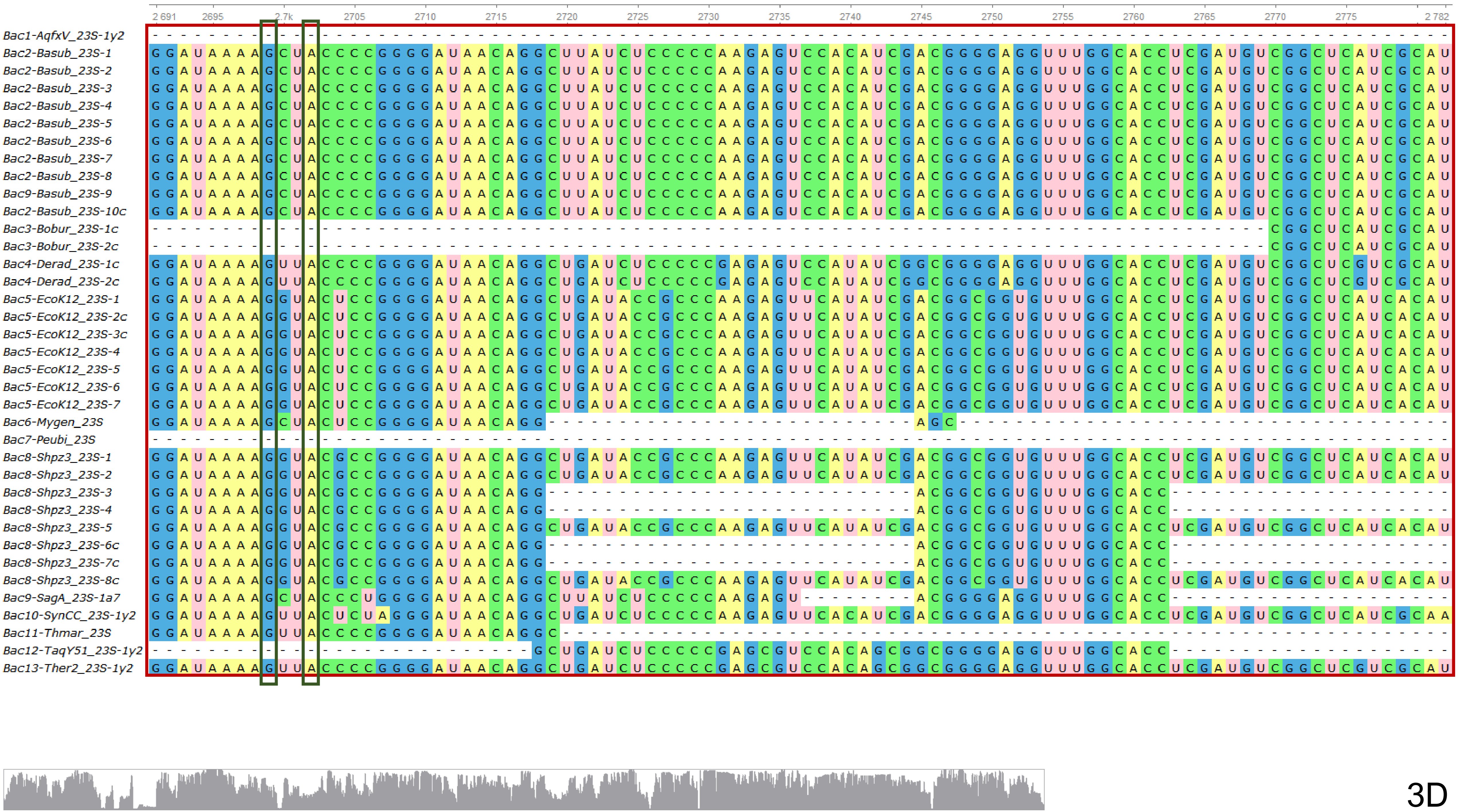
2 691
2695
2.7k
2705
2710
2715
2720
2725
2730
2735
2740
2745
2750
2755
2760
2765
2770
2775
2 782
Bac1-AqfxV_23S-1y2
Bac2-Basub_23S-1
Bac2-Basub_23S-2
Bac2-Basub_23S-3
Bac2-Basub_23S-4
Bac2-Basub_23S-5
Bac2-Basub_23S-6
Bac2-Basub_23S-7
Bac2-Basub_23S-8
Bac9-Basub_23S-9
Bac2-Basub_23S-10c
Bac3-Bobur_23S-1c
Bac3-Bobur_23S-2c
Bac4-Derad_23S-1c
Bac4-Derad_23S-2c
Bac5-EcoK12_23S-1
Bac5-EcoK12_23S-2c
Bac5-EcoK12_23S-3c
Bac5-EcoK12_23S-4
Bac5-EcoK12_23S-5
Bac5-EcoK12_23S-6
Bac5-EcoK12_23S-7
Bac6-Mygen_23S
Bac7-Peubi_23S
Bac8-Shpz3_23S-1
Bac8-Shpz3_23S-2
Bac8-Shpz3_23S-3
Bac8-Shpz3_23S-4
Bac8-Shpz3_23S-5
Bac8-Shpz3_23S-6c
Bac8-Shpz3_23S-7c
Bac8-Shpz3_23S-8c
Bac9-SagA_23S-1a7
Bac10-SynCC_23S-1y2
Bac11-Thmar_23S
Bac12-TaqY51_23S-1y2
Bac13-Ther2_23S-1y2

3D

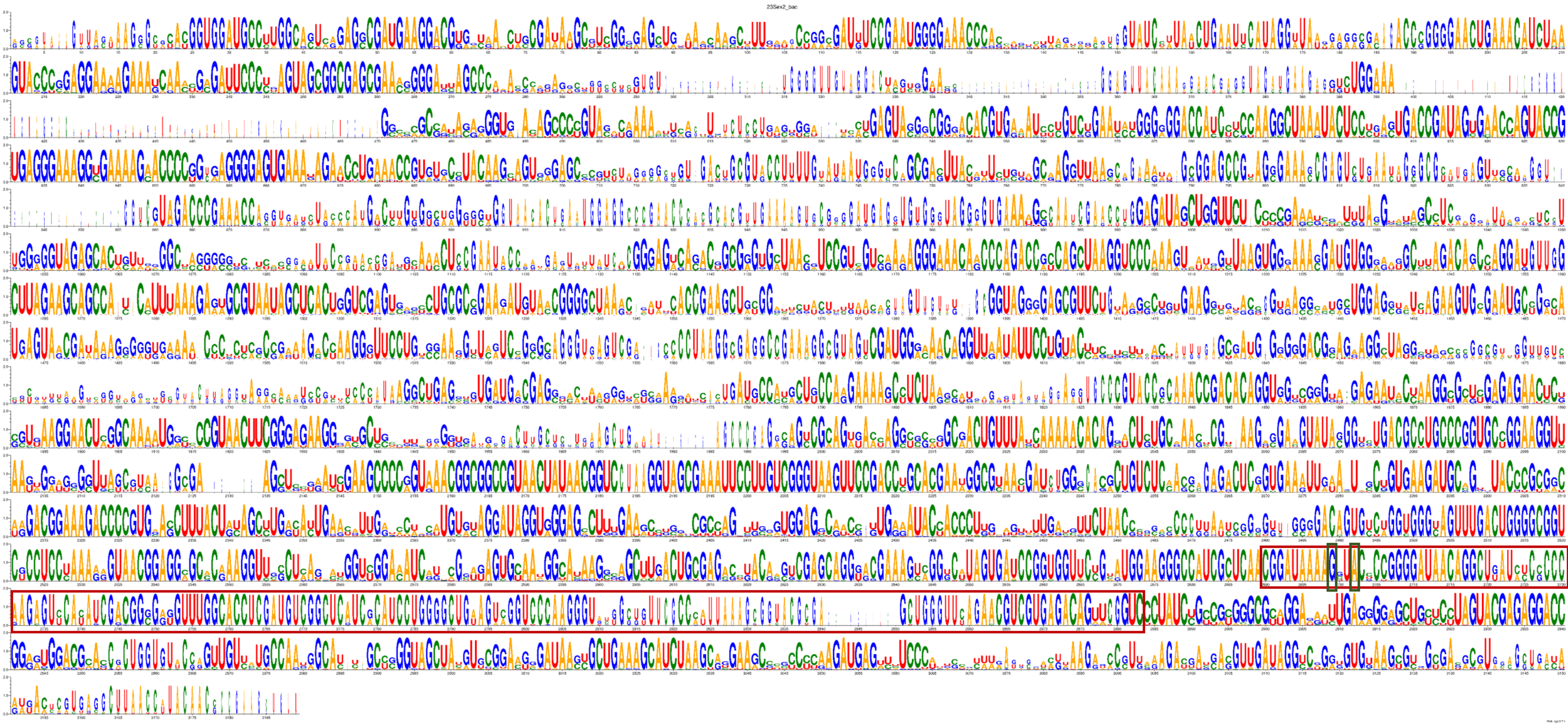

3D

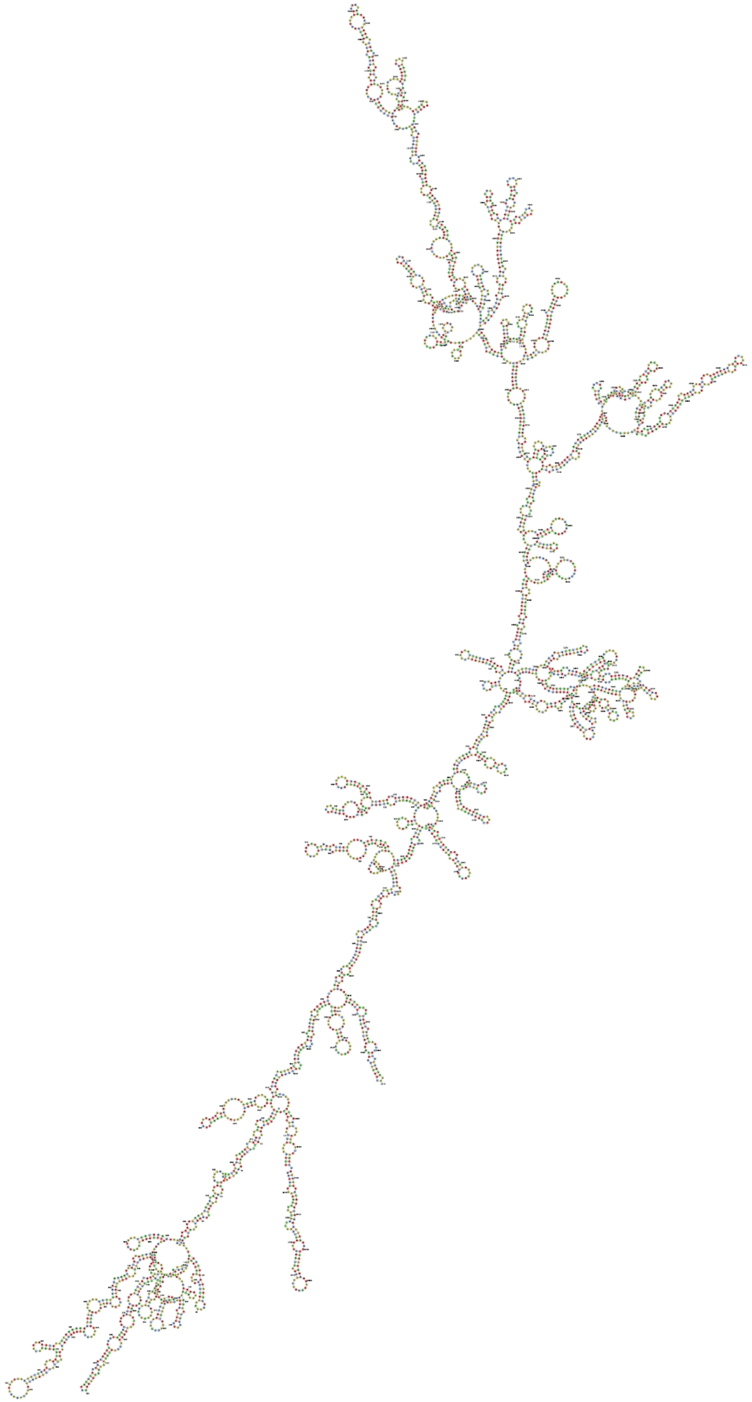

3D

6S RNA

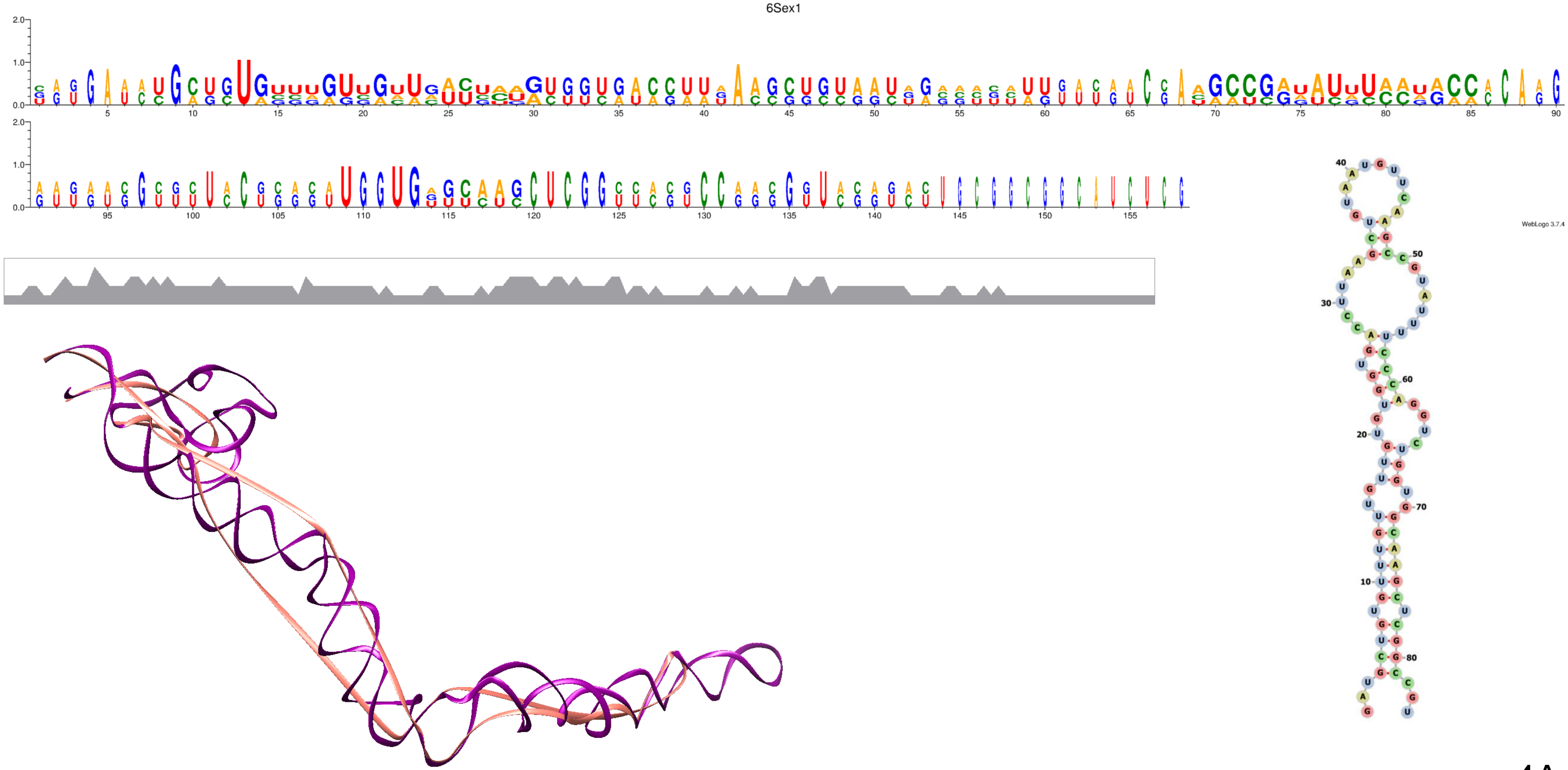

$TM_{score\,(2)} = 0.24615$

4A

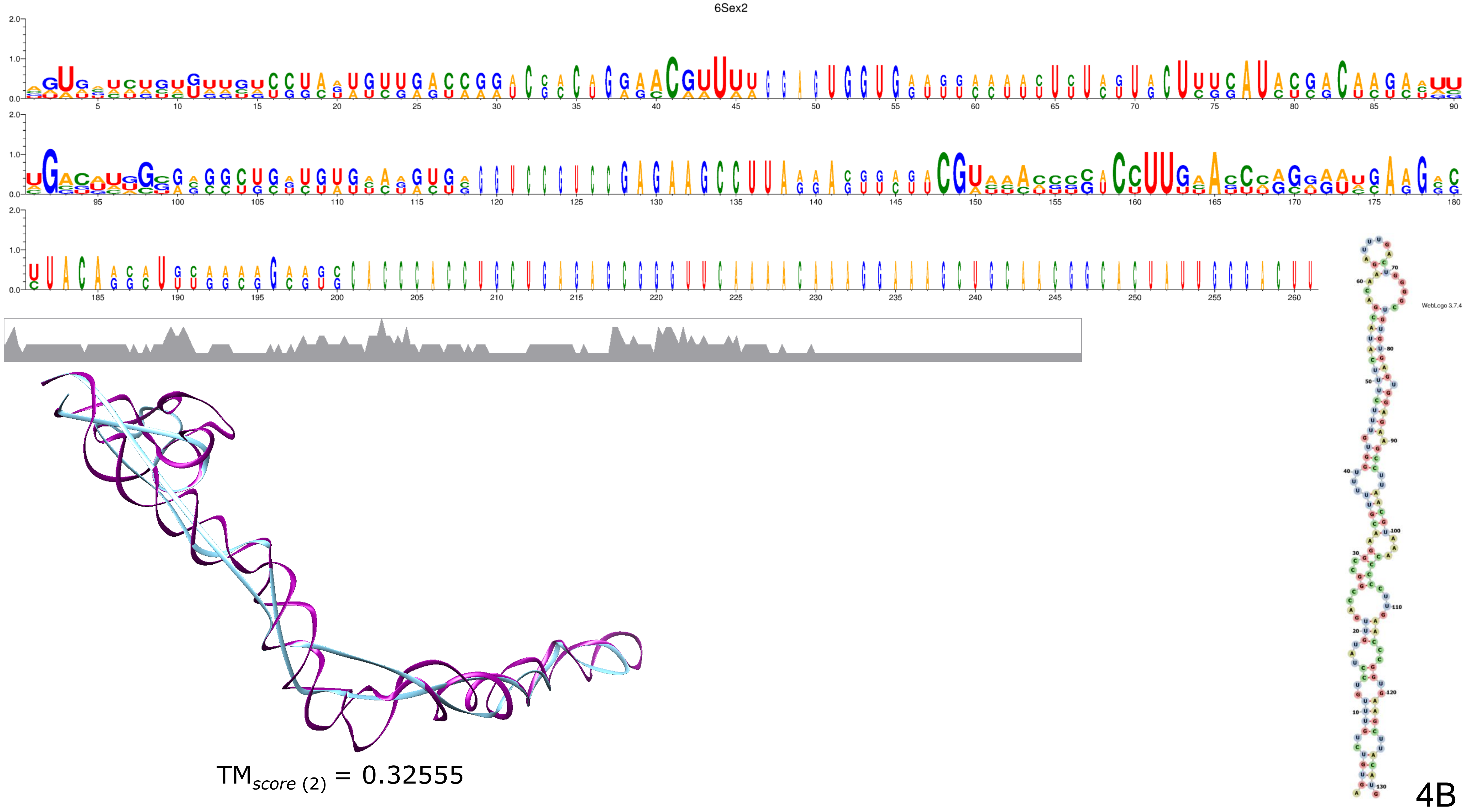
6Sex2
WebLogo 3.7.4
TM$_{score\ (2)}$ = 0.32555
4B

SRP-RNA

Order of helixes along SRP-RNA sequences

5’H1 – 5’H2 – H3 – Helix4 – 3’H2 – 5’endHelix5 – H6 – H8 – 3’endHelix5 – 3’H1

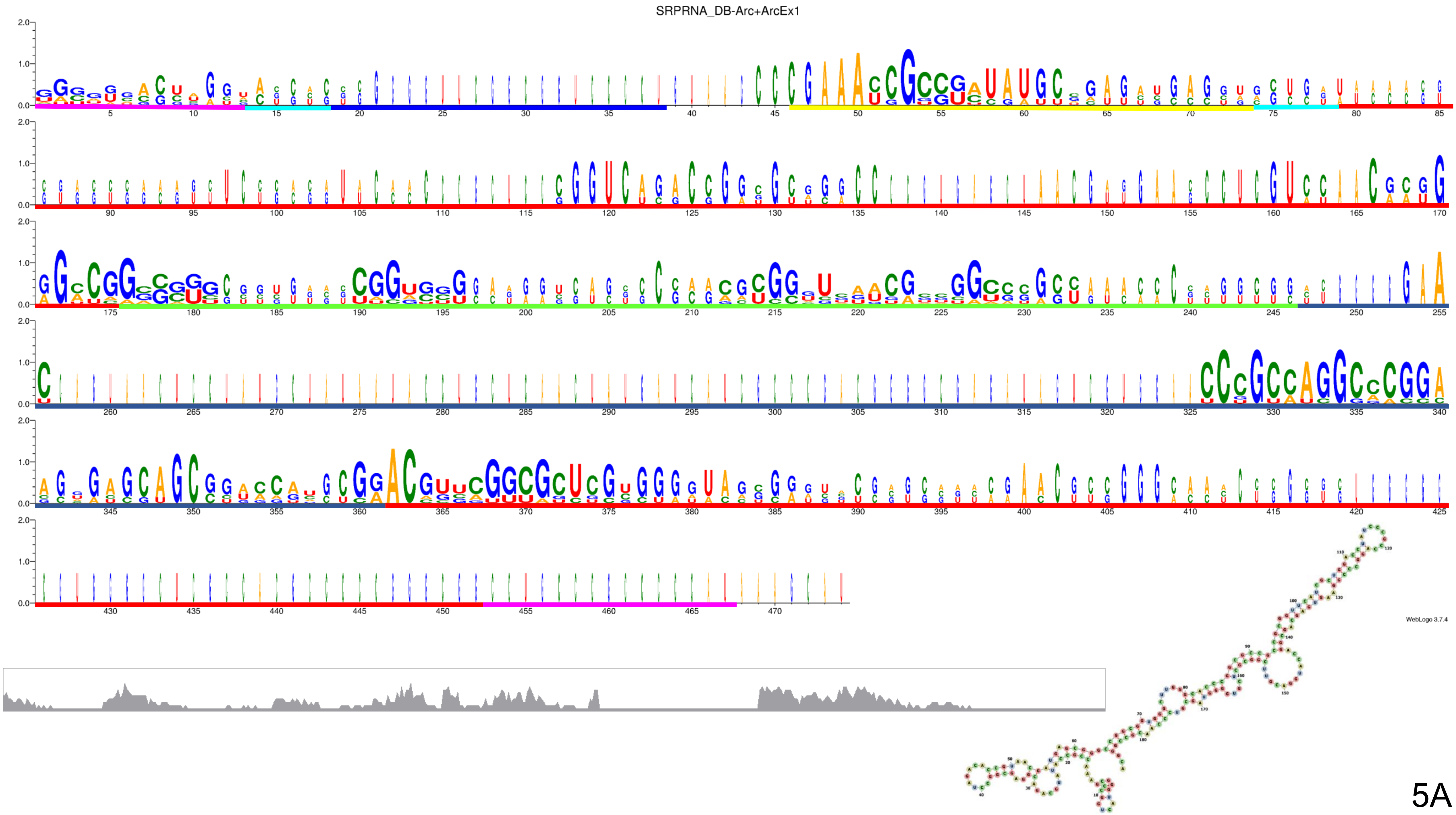

5A

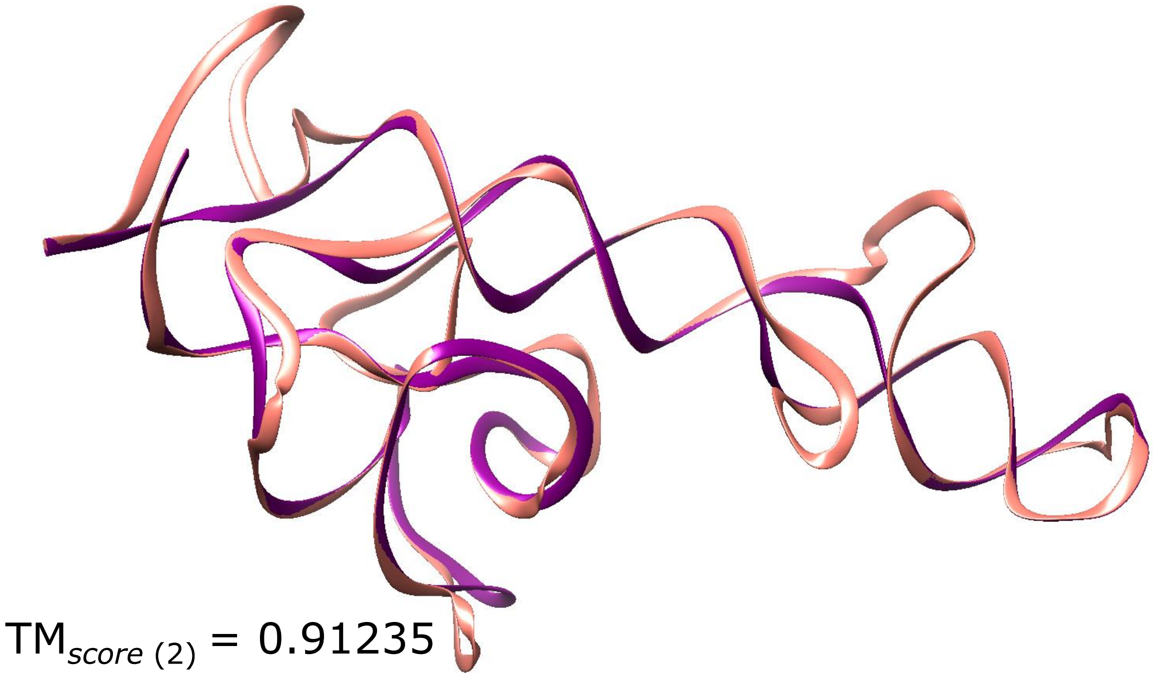

5A

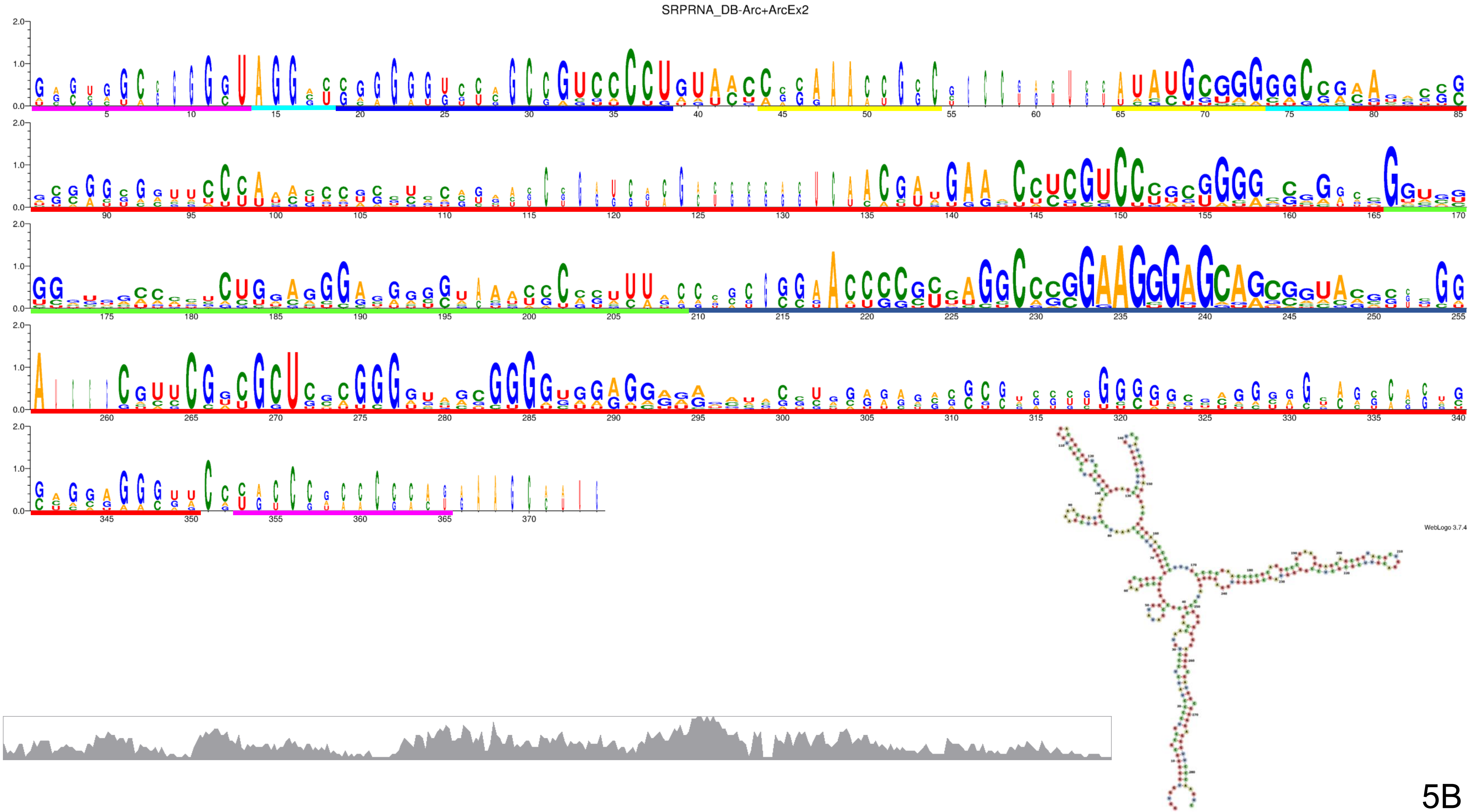

5B

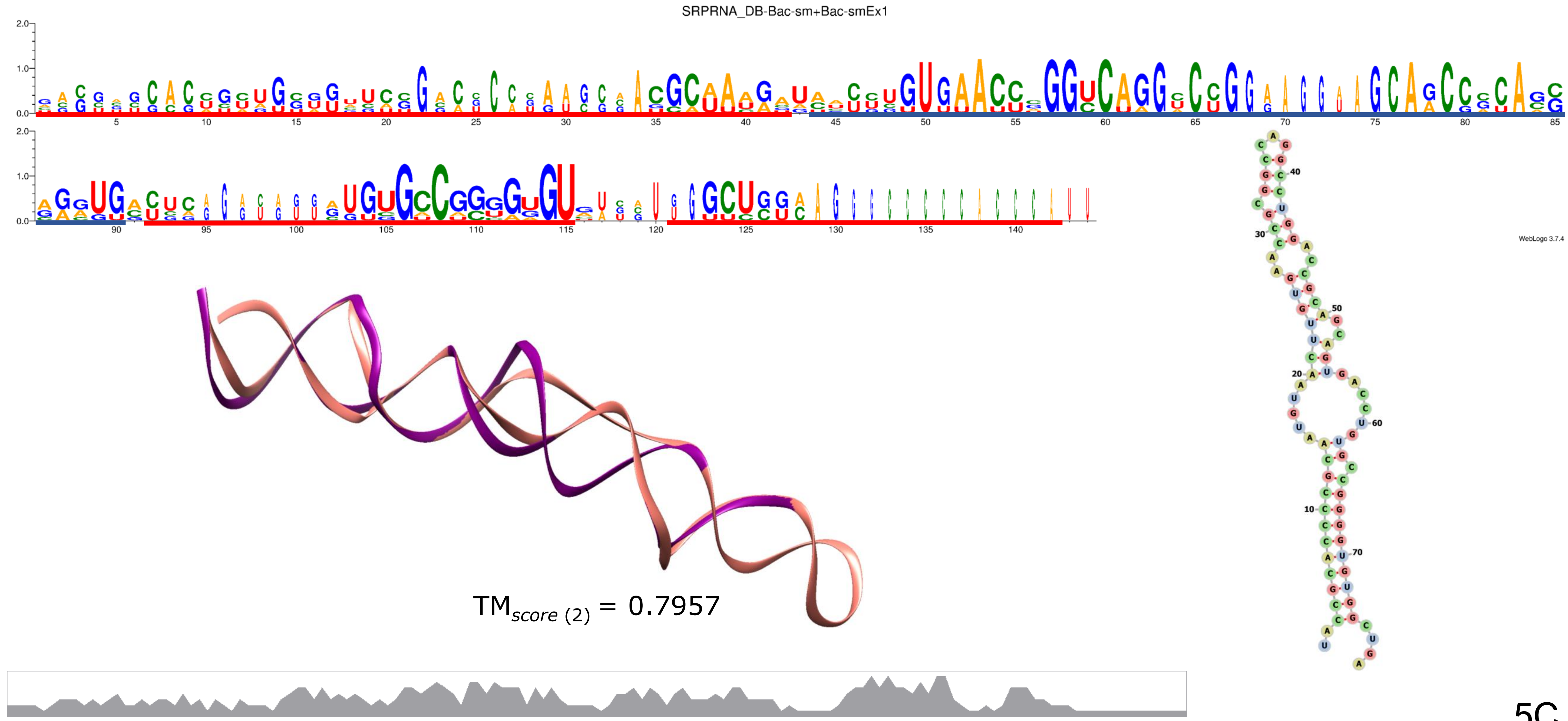

5C

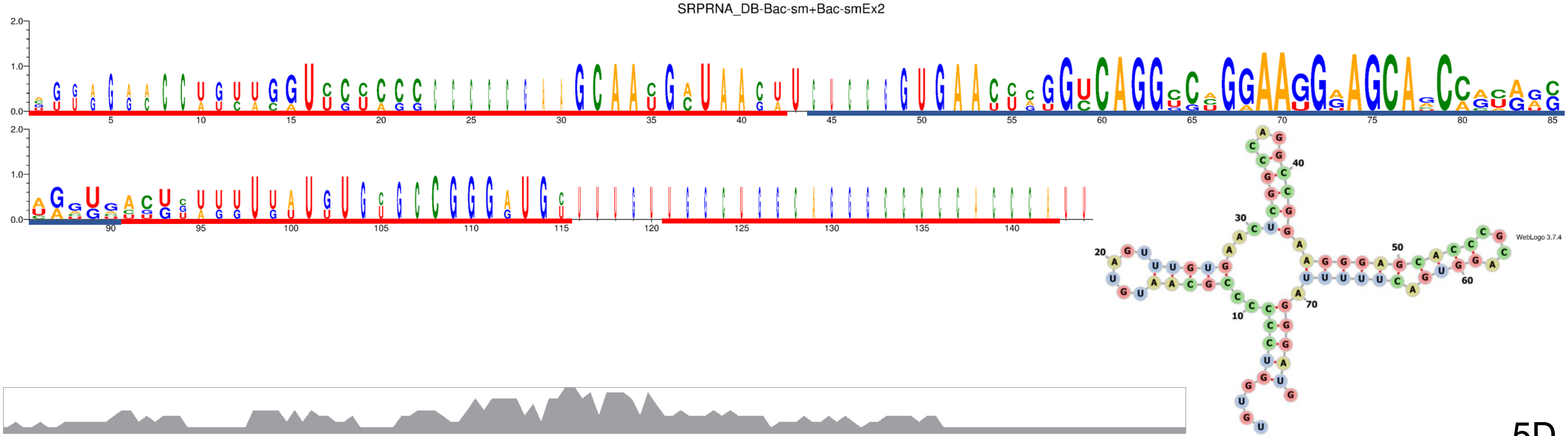

5D

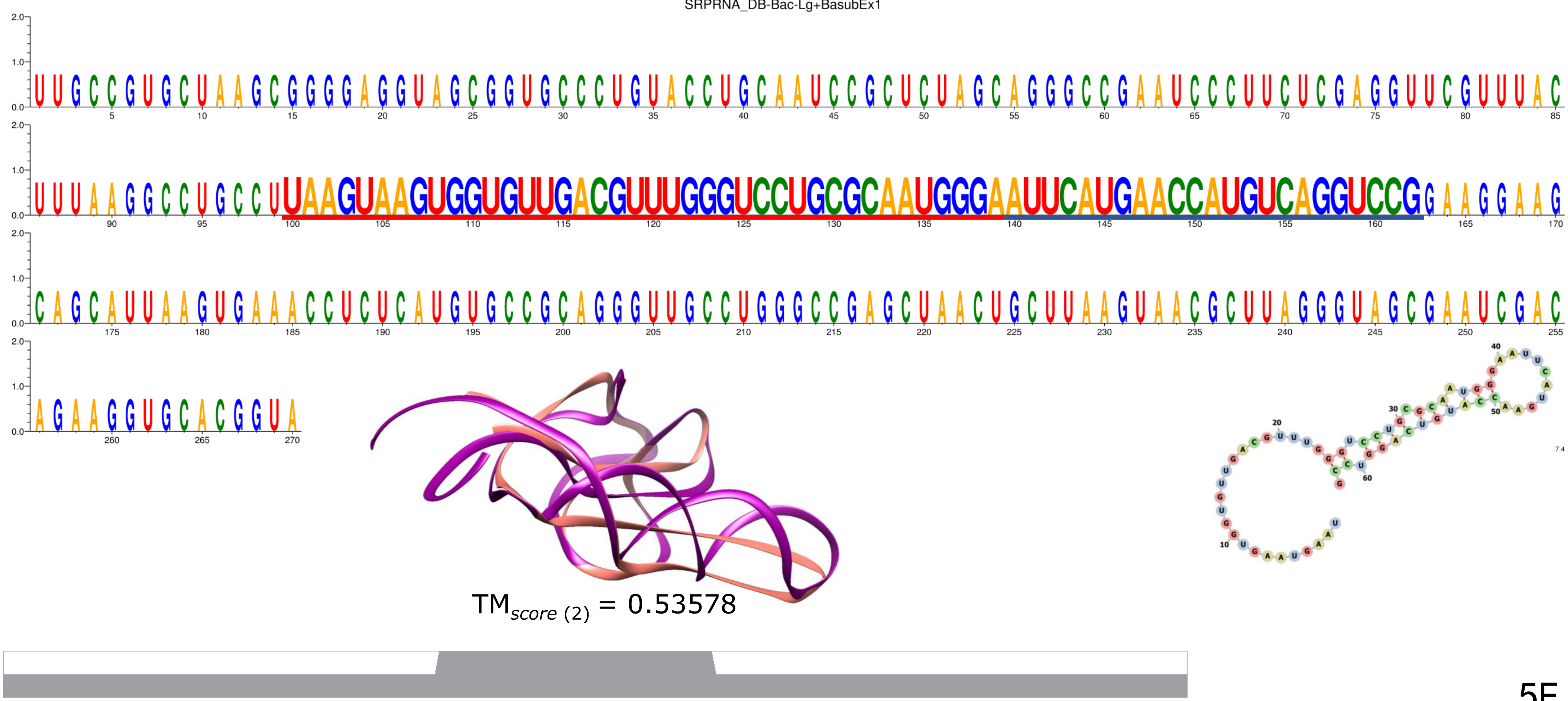

5E

SRPRNA_DB-Bac-Lg+BasubEx2

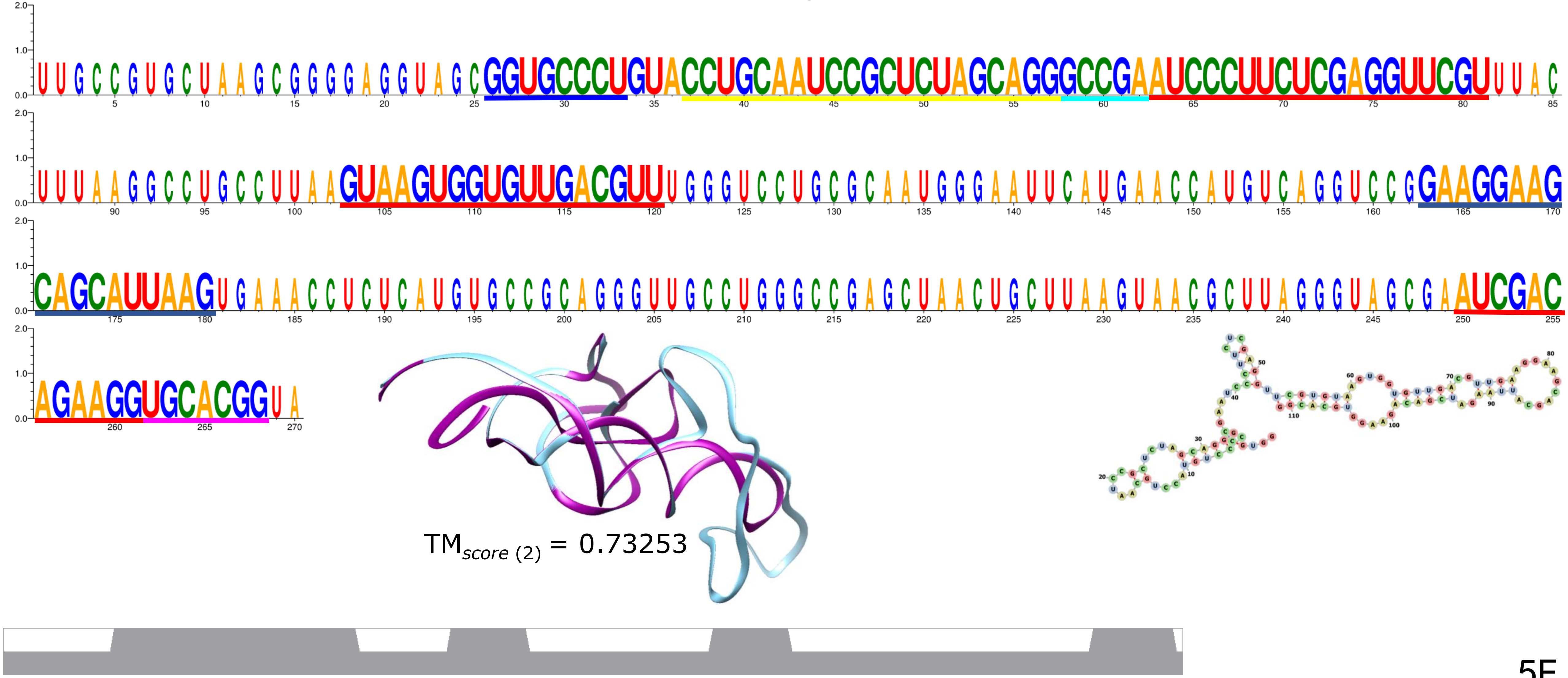

5F

tmRNA

Order of portions along tmRNA sequences

1 to 27 [G3] – 28 to 48 – 49 to 302 [A86 – 90 to 122 [G90,C91,A92 - U120,121A,122A]] – 303 to 325 – 1 to 27[U357]

D-arm 5′ – helical spacer 5′ – MLD with PKs [universal – tag peptide [resume codon – stop codon]] – helical spacer 3′ –T-arm 3′

TLD 5′ **MLD** **TLD 3′**

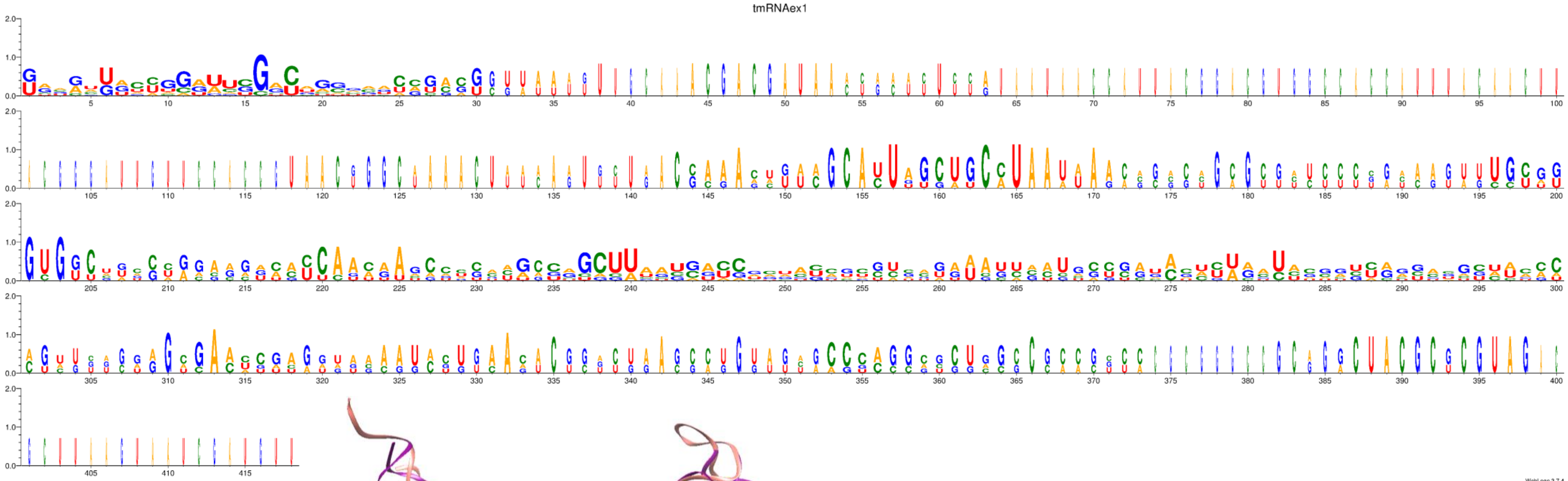

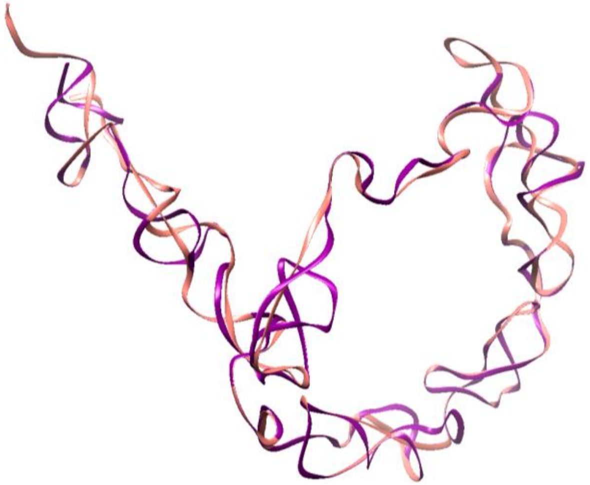

$TM_{score\ (2)} = 0.72324$

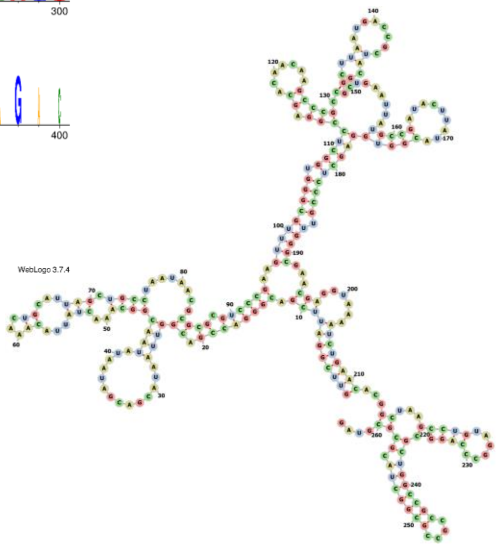

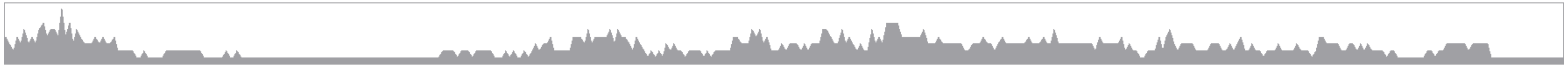

6A

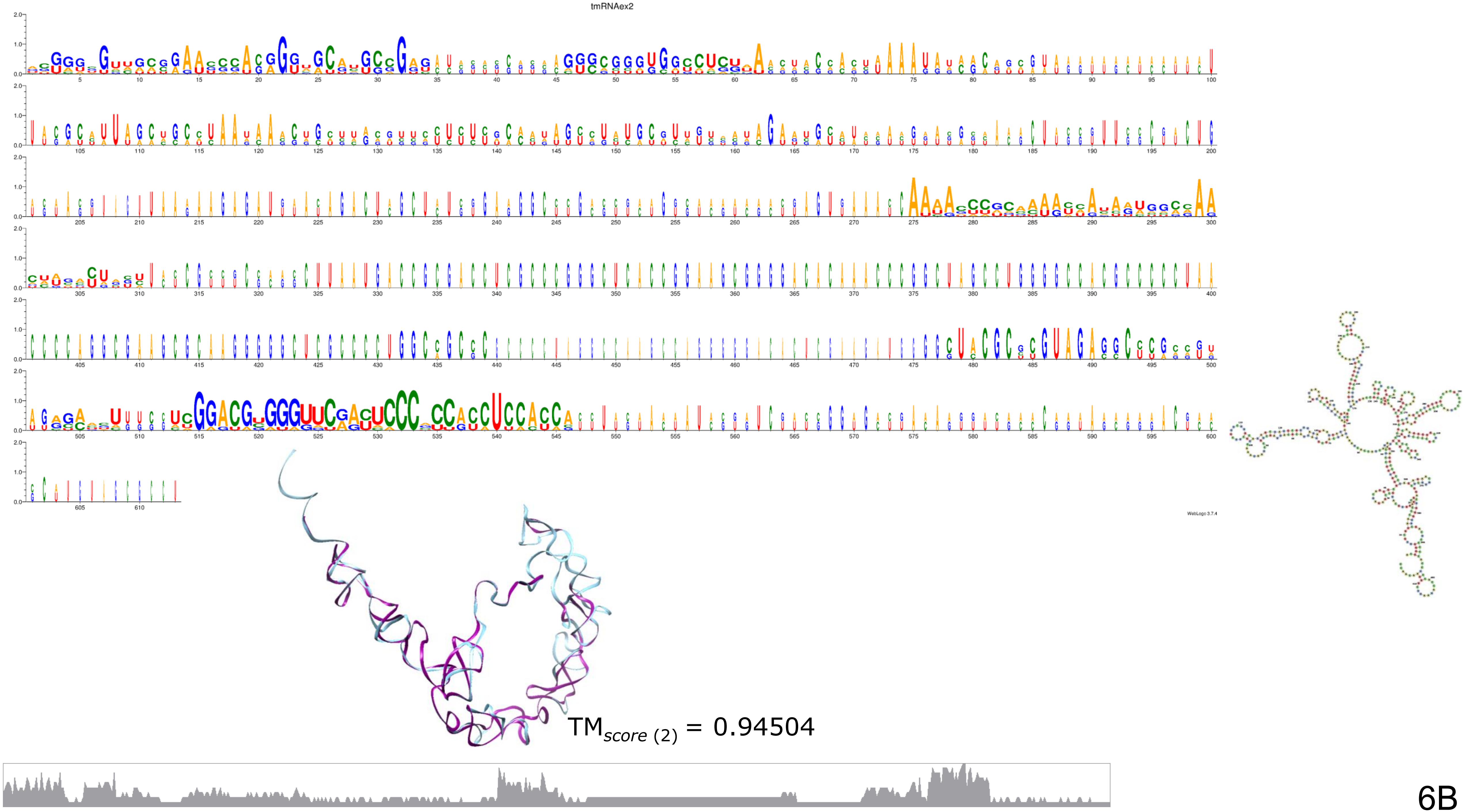

6B

RNA-P

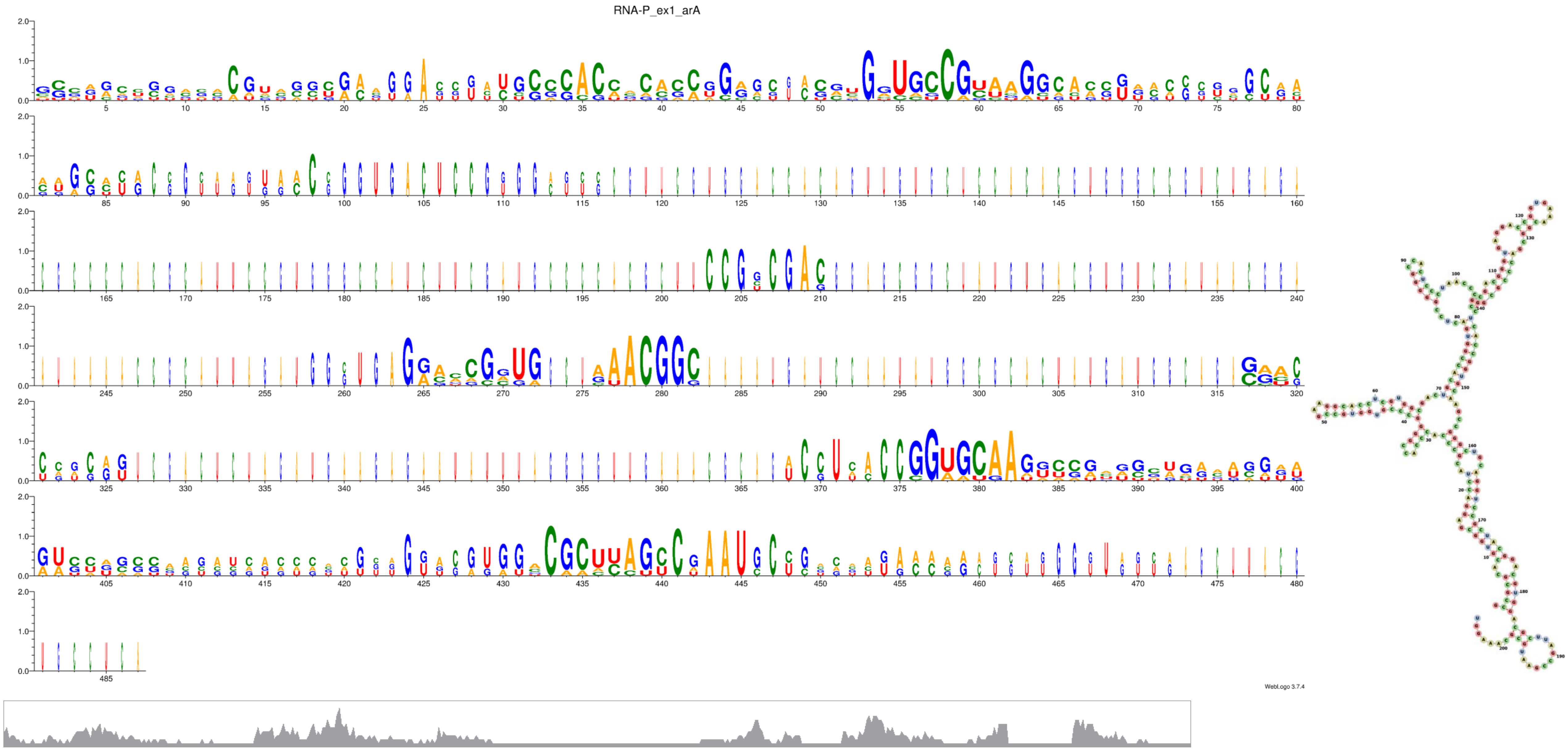

7A

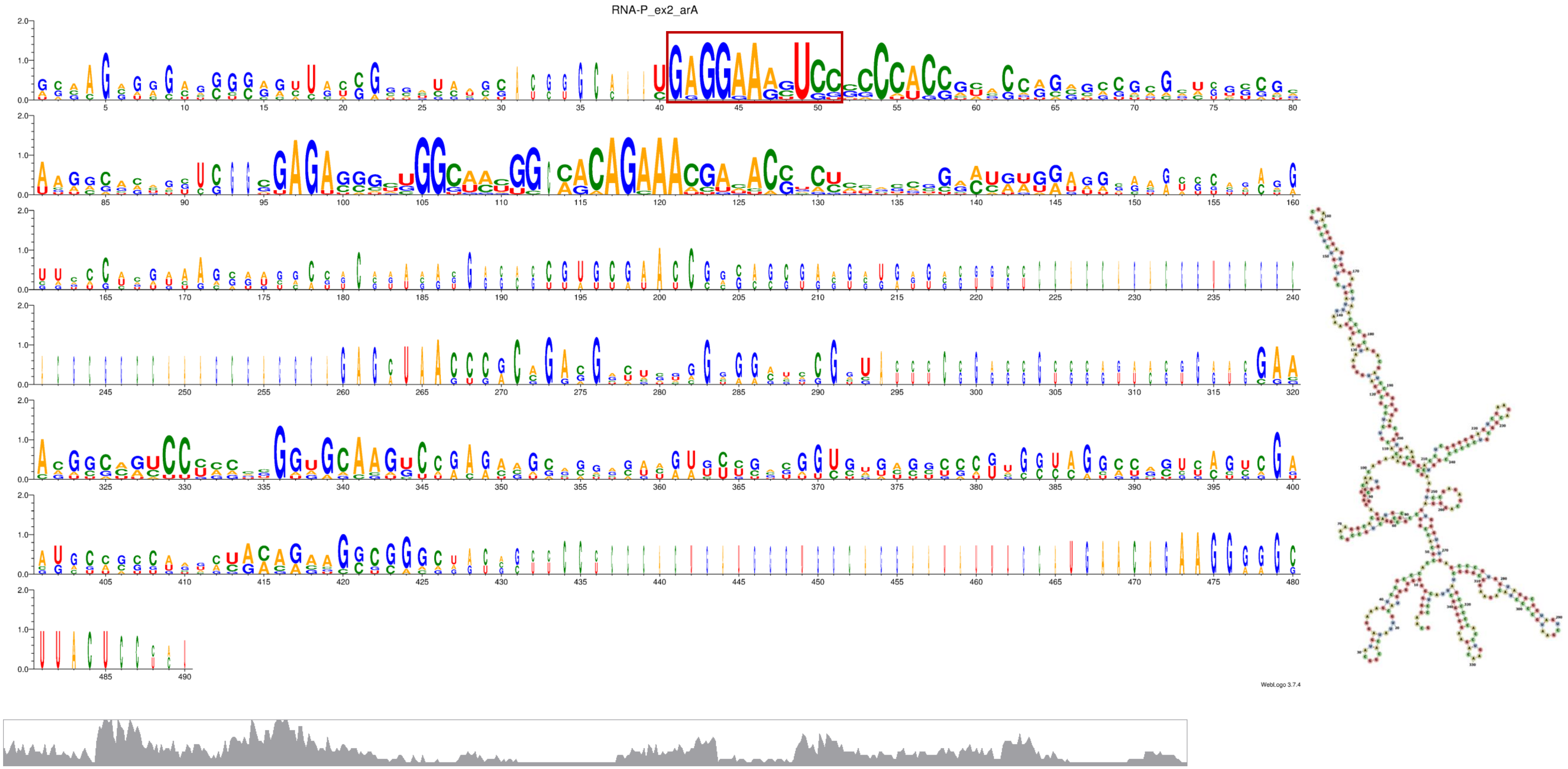

7B

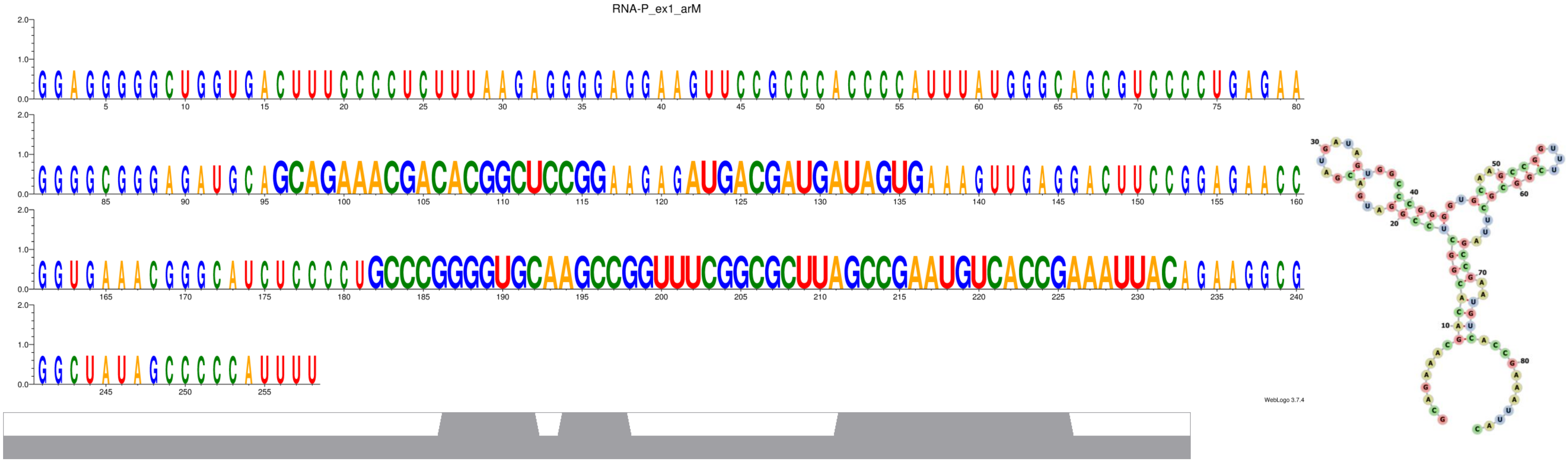

7C

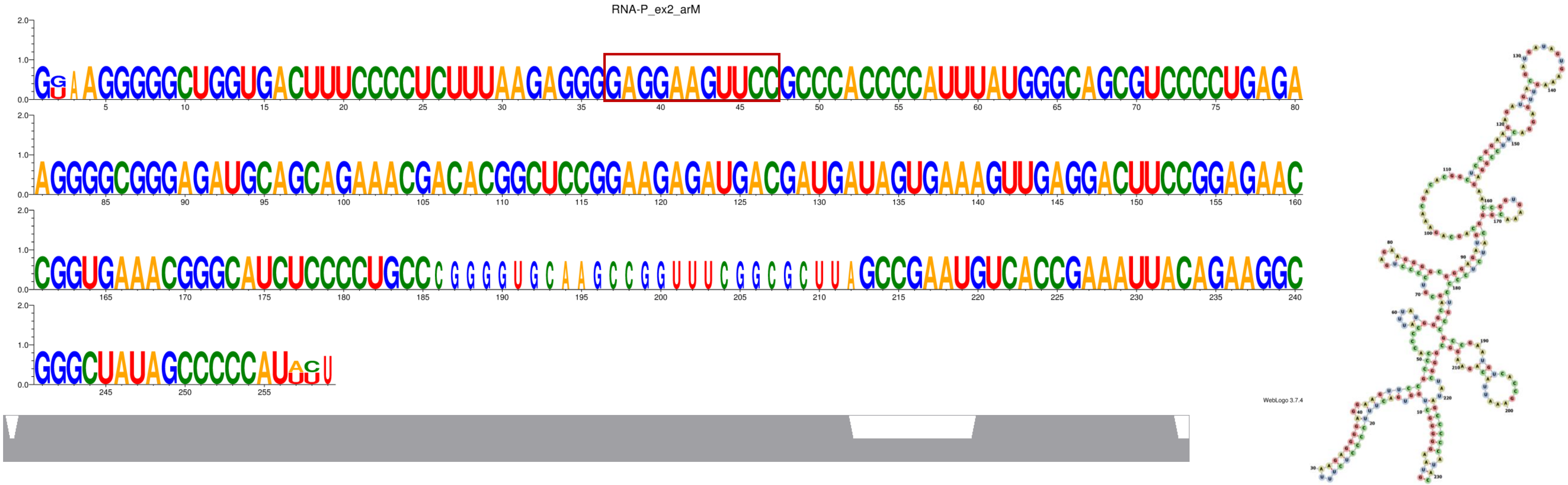

7D

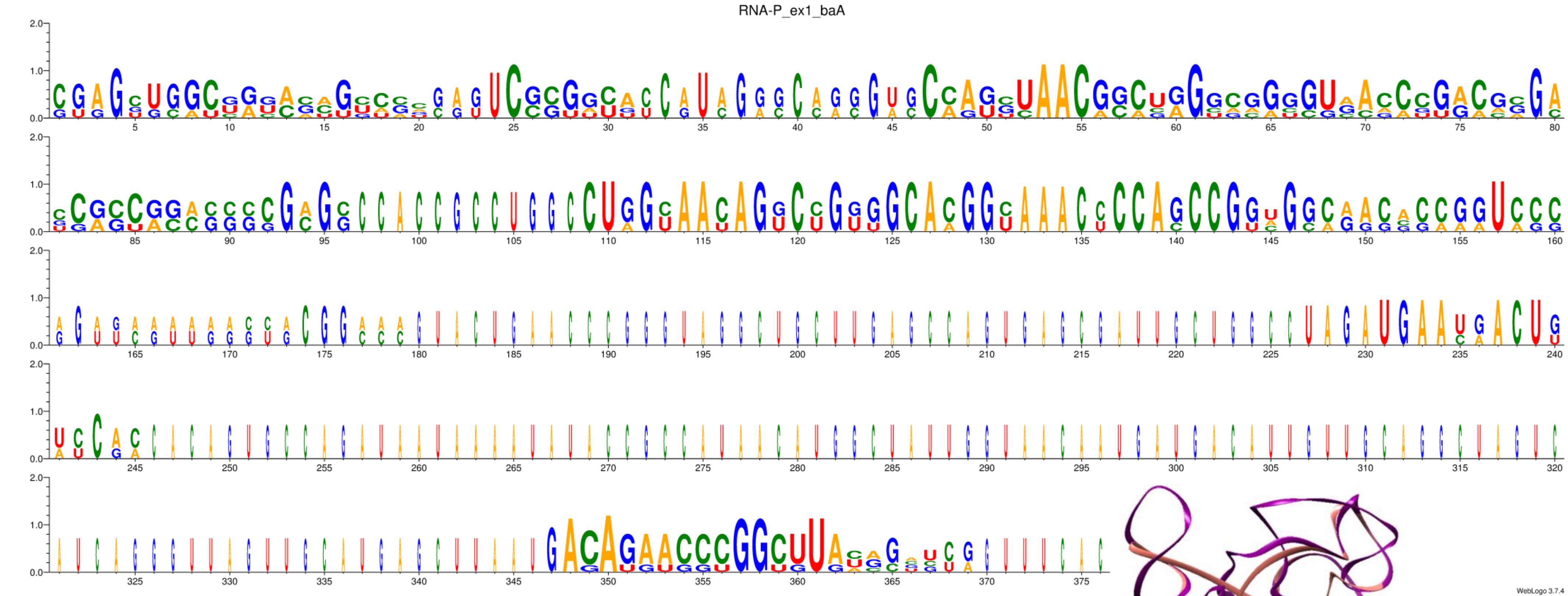

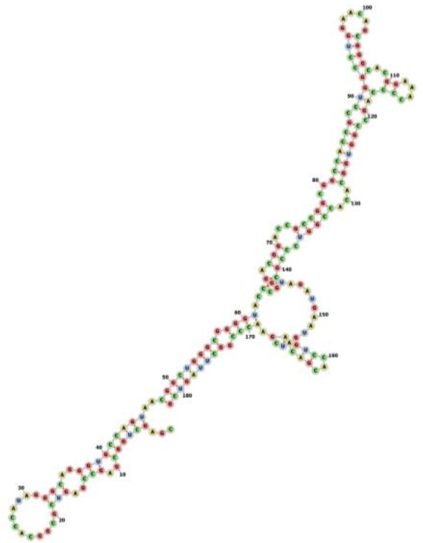

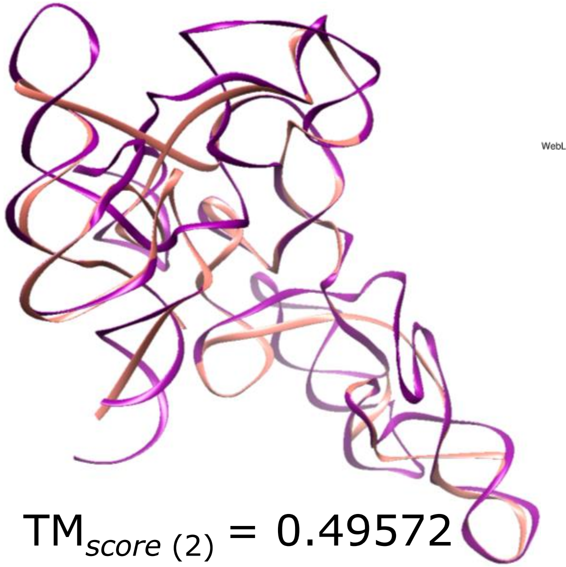

$TM_{score\ (2)} = 0.49572$

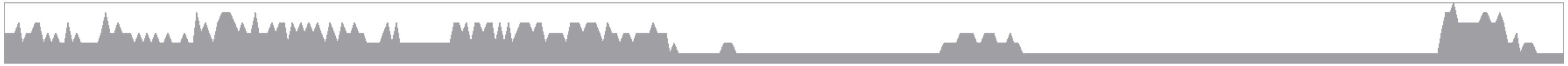

7E

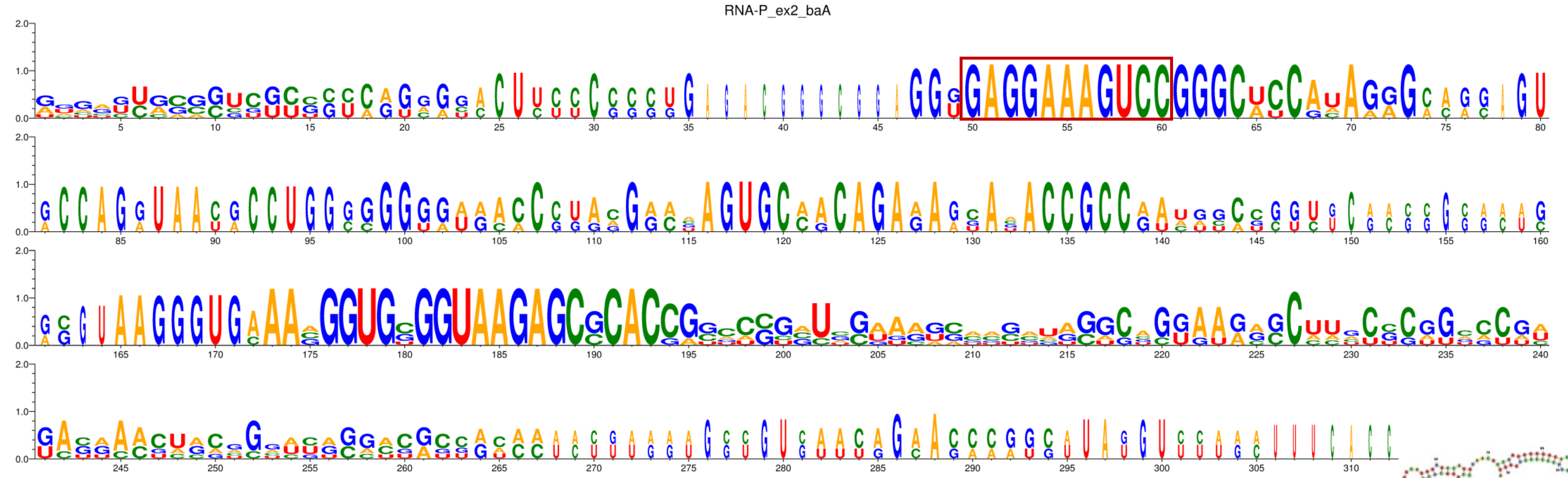

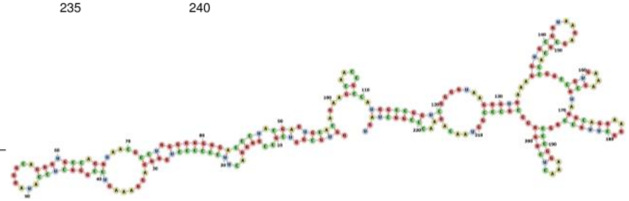

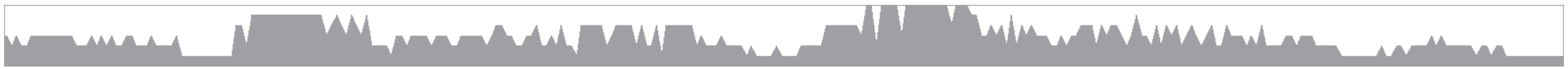

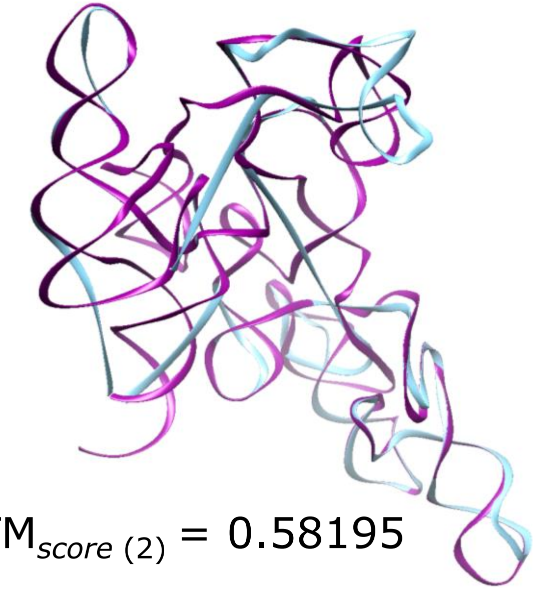

$TM_{score\ (2)} = 0.58195$

7F

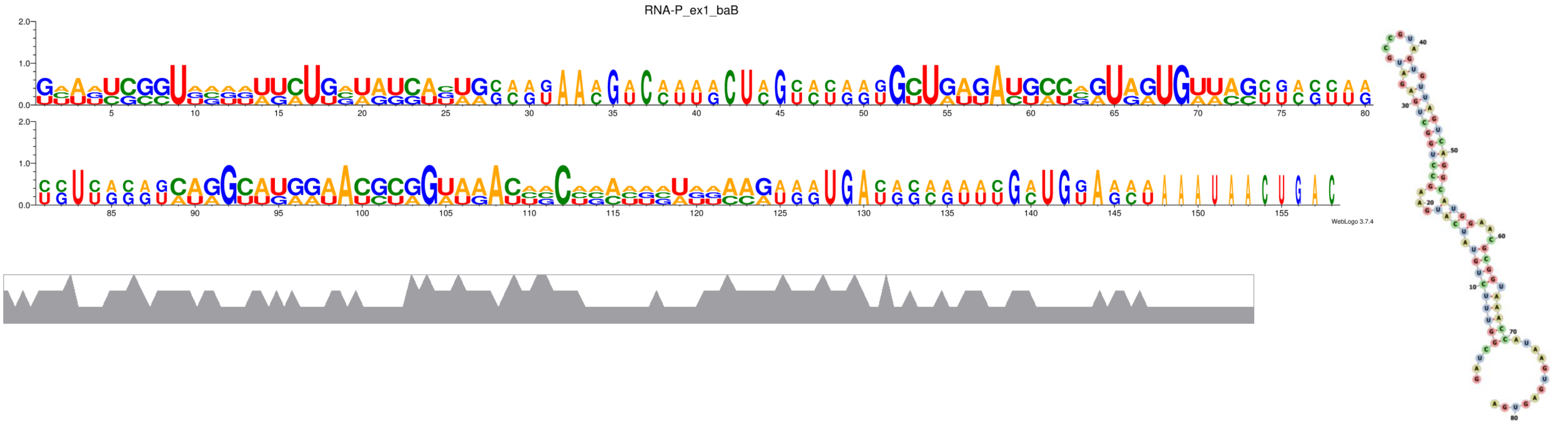

7G

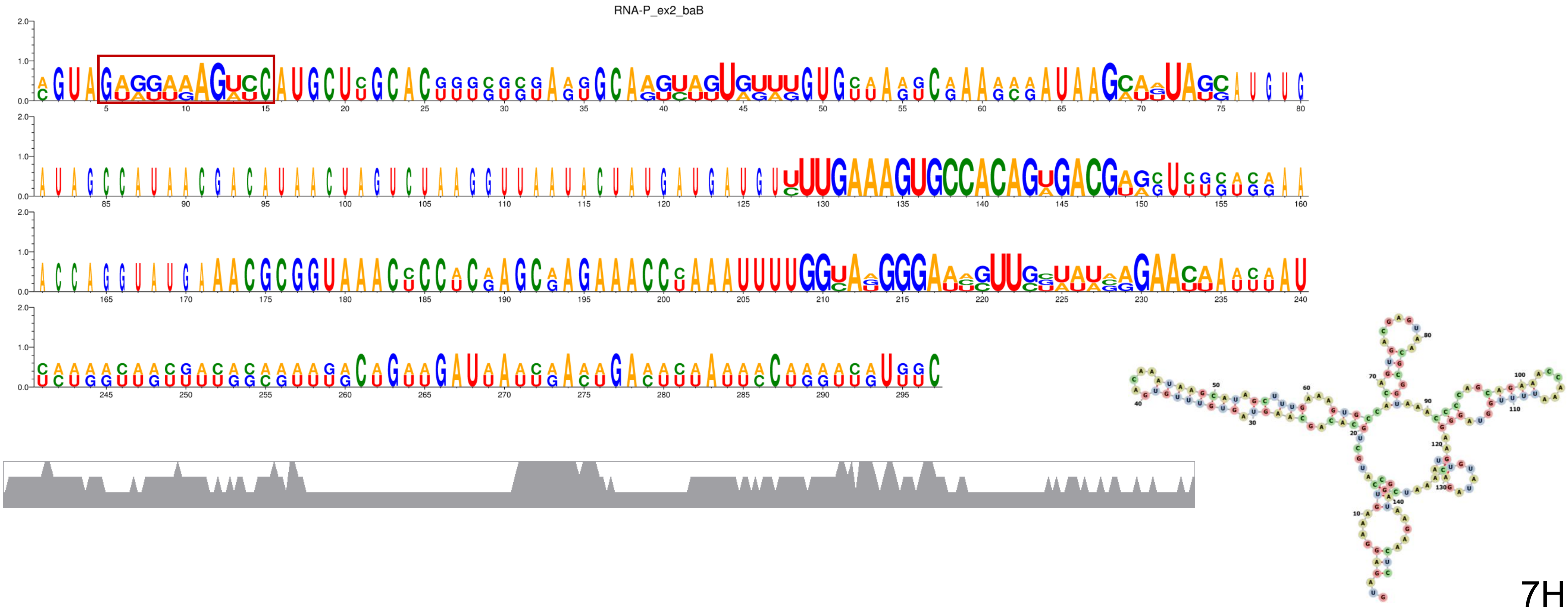

7H

tRNAs

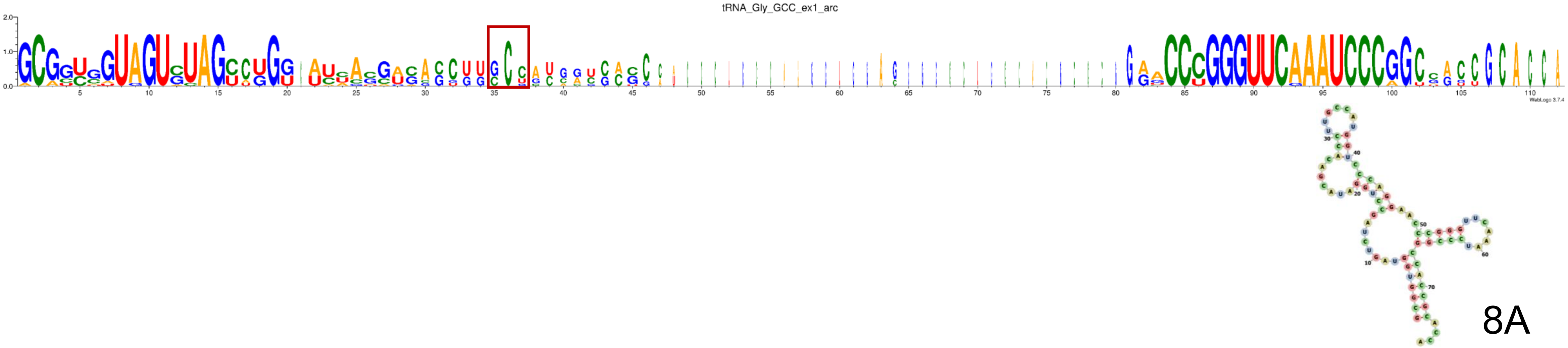
tRNA_Gly_GCC_ex1_arc
WebLogo 3.7.4

8A

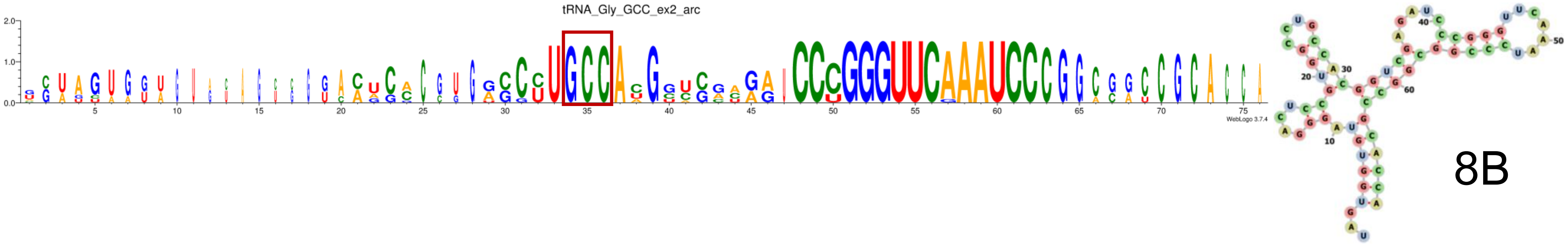
tRNA_Gly_GCC_ex2_arc
WebLogo 3.7.4

8B

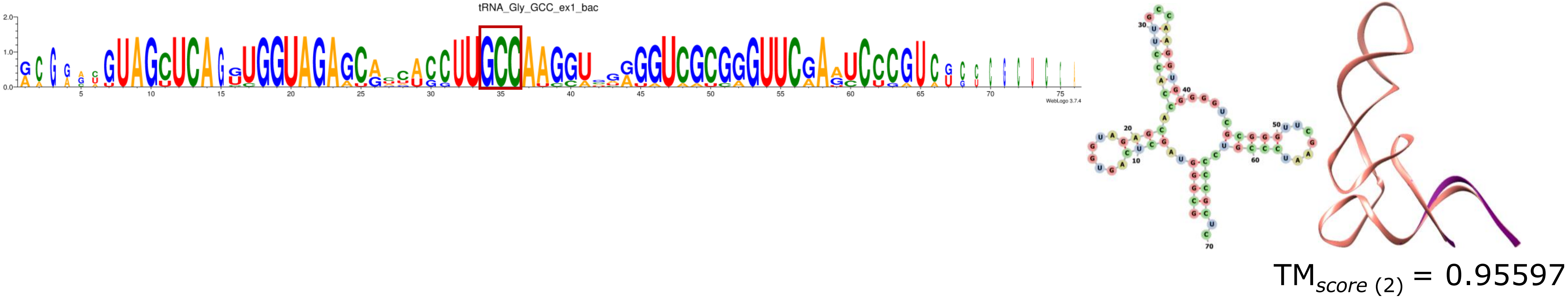
tRNA_Gly_GCC_ex1_bac
TM$_{score\ (2)}$ = 0.95597

8C

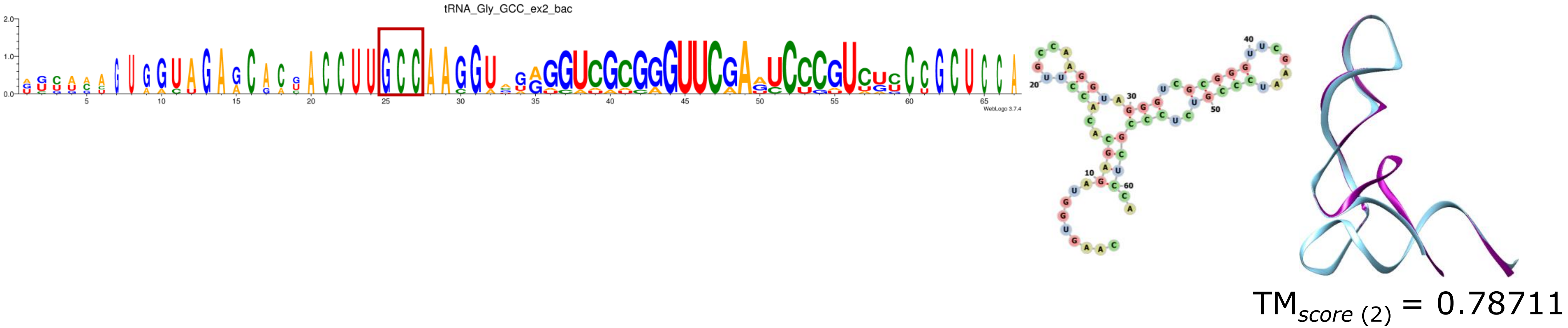
tRNA_Gly_GCC_ex2_bac
TM$_{score\ (2)}$ = 0.78711

8D

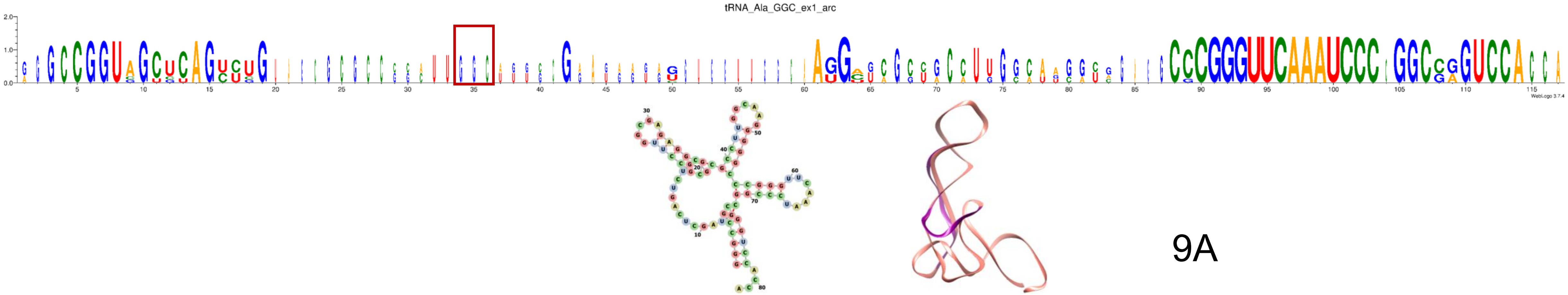

$TM_{score\ (2)} = 0.9318$

9A

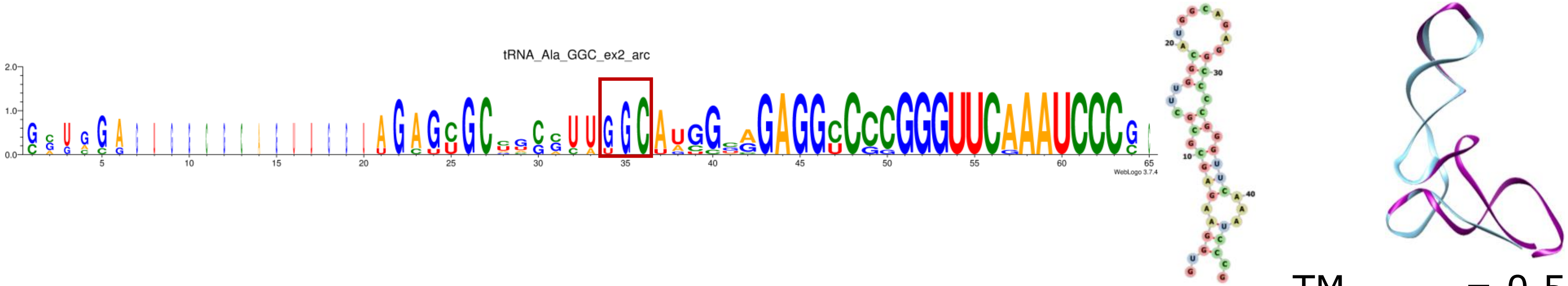

$TM_{score\ (2)} = 0.58445$

9B

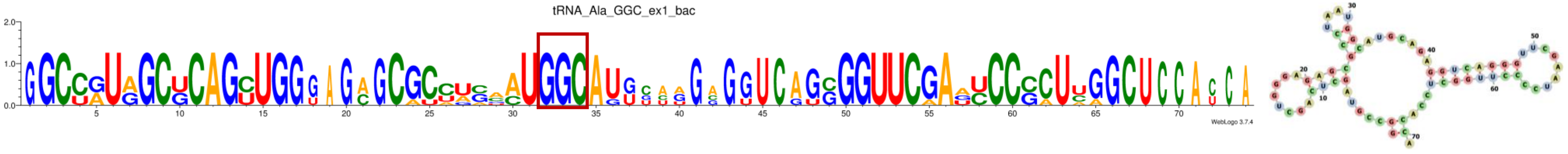
tRNA_Ala_GGC_ex1_bac
WebLogo 3.7.4

9C

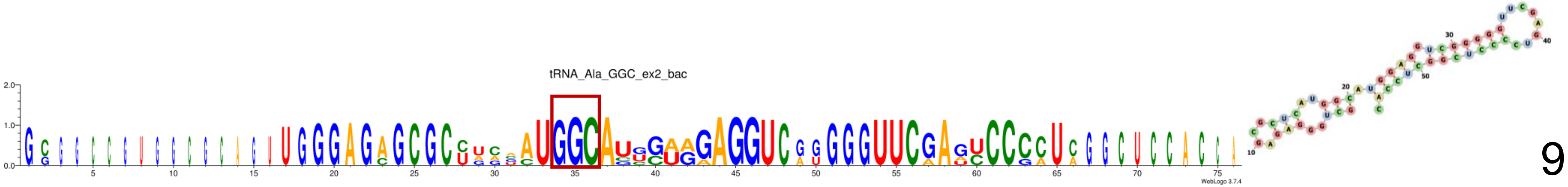
tRNA_Ala_GGC_ex2_bac
WebLogo 3.7.4

9D

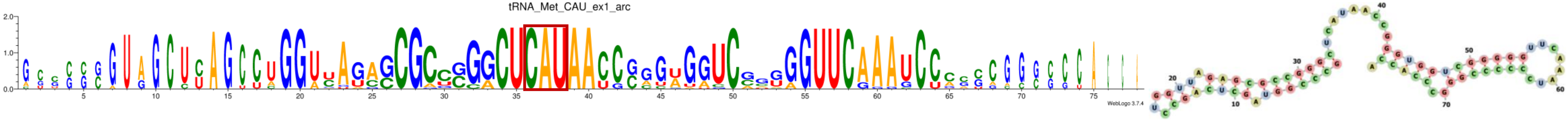
tRNA_Met_CAU_ex1_arc
WebLogo 3.7.4

10A

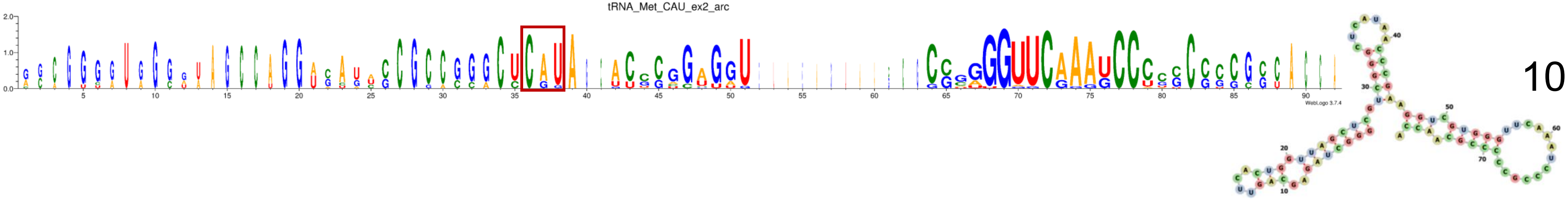
tRNA_Met_CAU_ex2_arc
WebLogo 3.7.4

10B

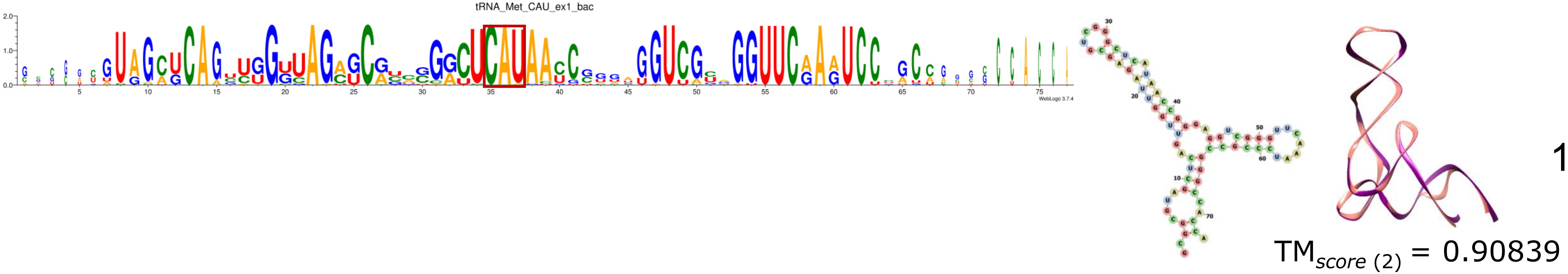
tRNA_Met_CAU_ex1_bac
WebLogo 3.7.4
TM$_{score\ (2)}$ = 0.90839

10C

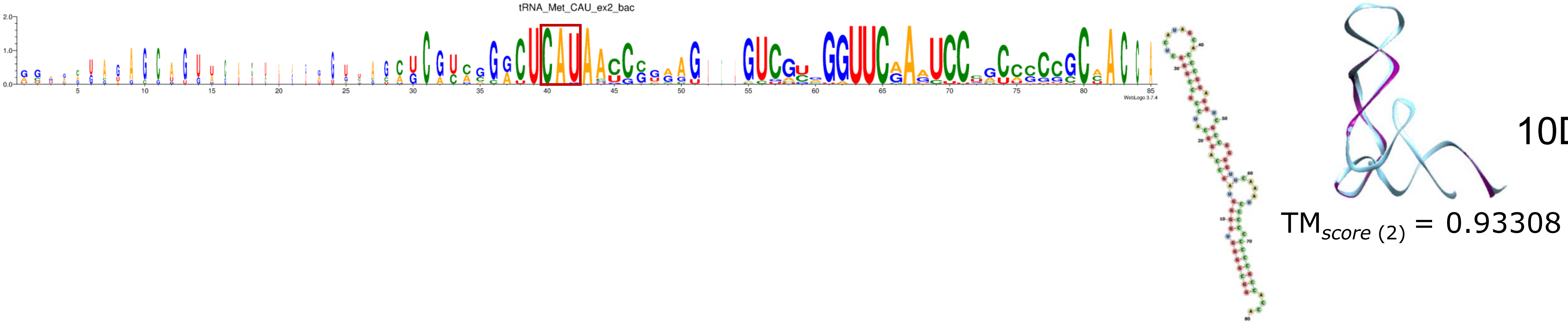
tRNA_Met_CAU_ex2_bac
WebLogo 3.7.4
TM$_{score\ (2)}$ = 0.93308

10D

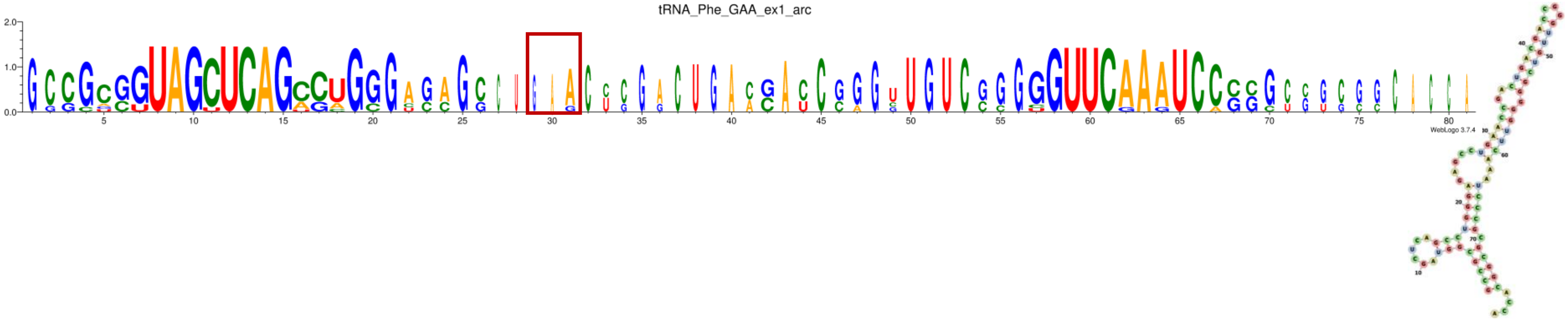
tRNA_Phe_GAA_ex1_arc
WebLogo 3.7.4

11A

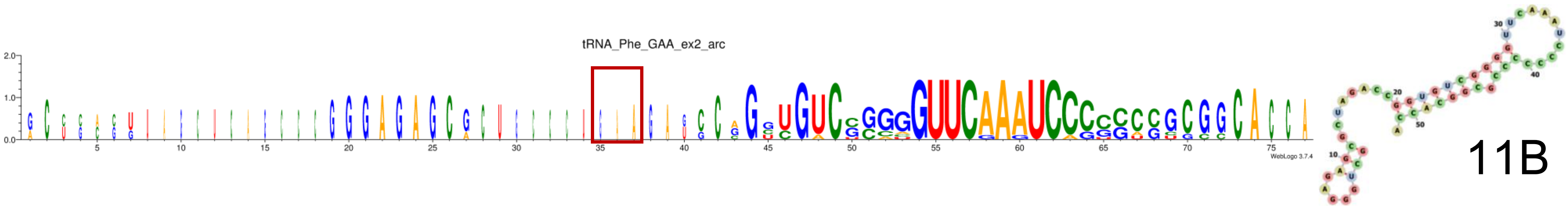
tRNA_Phe_GAA_ex2_arc
WebLogo 3.7.4

11B

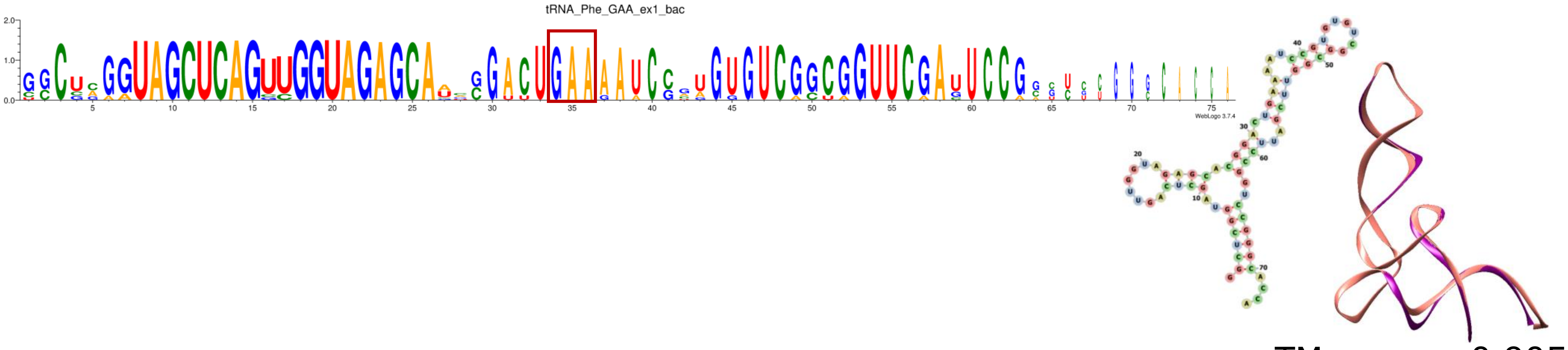

$TM_{score\ (2)} = 0.90501$

11C

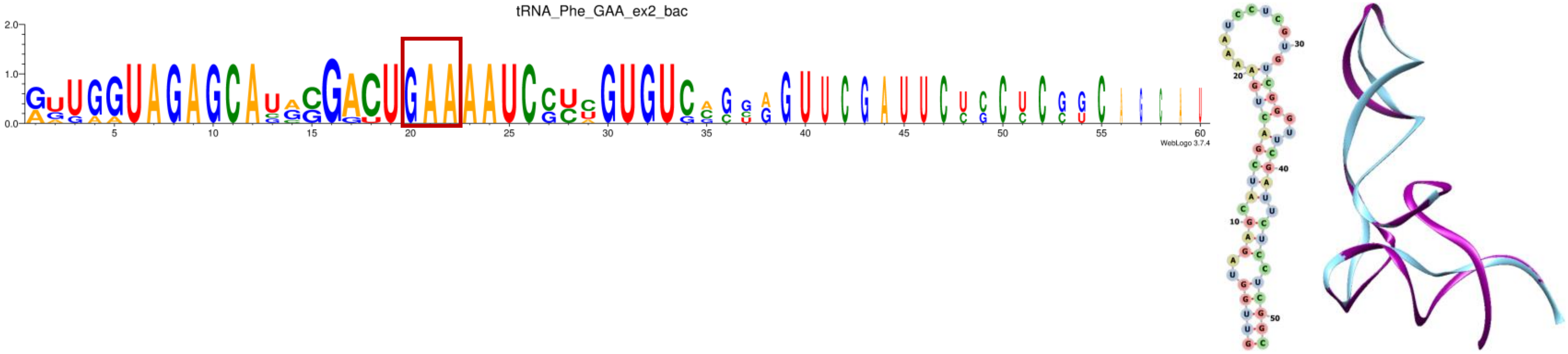

$TM_{score\ (2)} = 0.64502$

11D

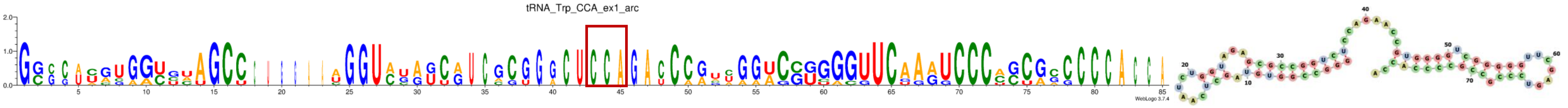
tRNA_Trp_CCA_ex1_arc
WebLogo 3.7.4

12A

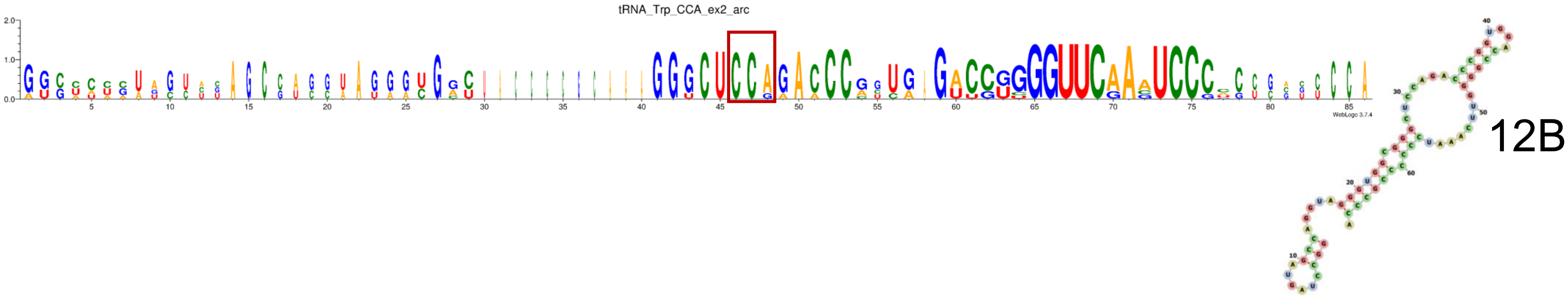
tRNA_Trp_CCA_ex2_arc
WebLogo 3.7.4

12B

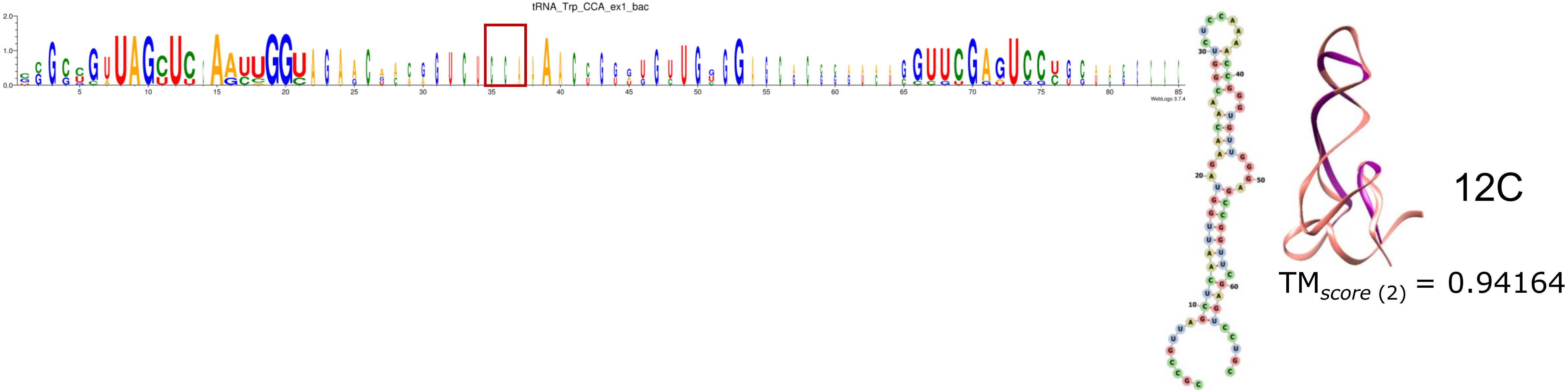
tRNA_Trp_CCA_ex1_bac
WebLogo 3.7.4
$TM_{score\ (2)} = 0.94164$

12C

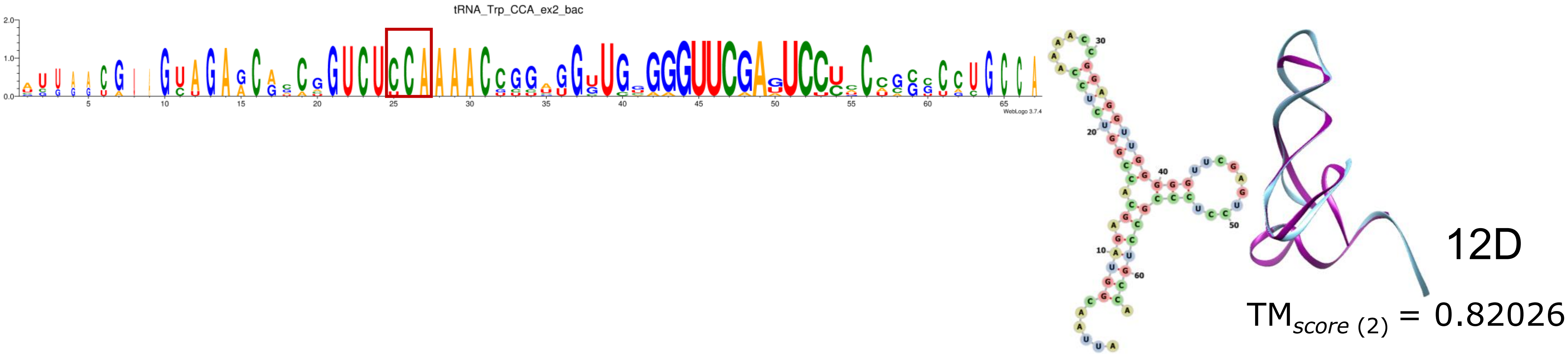
tRNA_Trp_CCA_ex2_bac
WebLogo 3.7.4
$TM_{score\ (2)} = 0.82026$

12D
