# Supplementary material for: A Proposal for the RNAome at the Dawn of the Last Universal Common Ancestor
Source: Genes (Basel). 2024 Sep 11;15(9):1195. doi: 10.3390/genes15091195 (PMC11431127; doi:10.3390/genes15091195)

S1. Supplementary material for

A proposal of the RNAome to the very dawn of LUCA

Miryam Palacios-Pérez [a] & Marco V. José [a,*]

[a] *Theoretical Biology Group, Instituto de Investigaciones Biomédicas, Universidad Nacional Autónoma de México, Ciudad de México CDMX, C.P. 04510, México*
First author E-mail address: mir.pape@iibiomedicas.unam.mx
[*] *Corresponding author E-mail address*: marcojose@biomedicas.unam.mx

Structural modelling of the extended RNAome

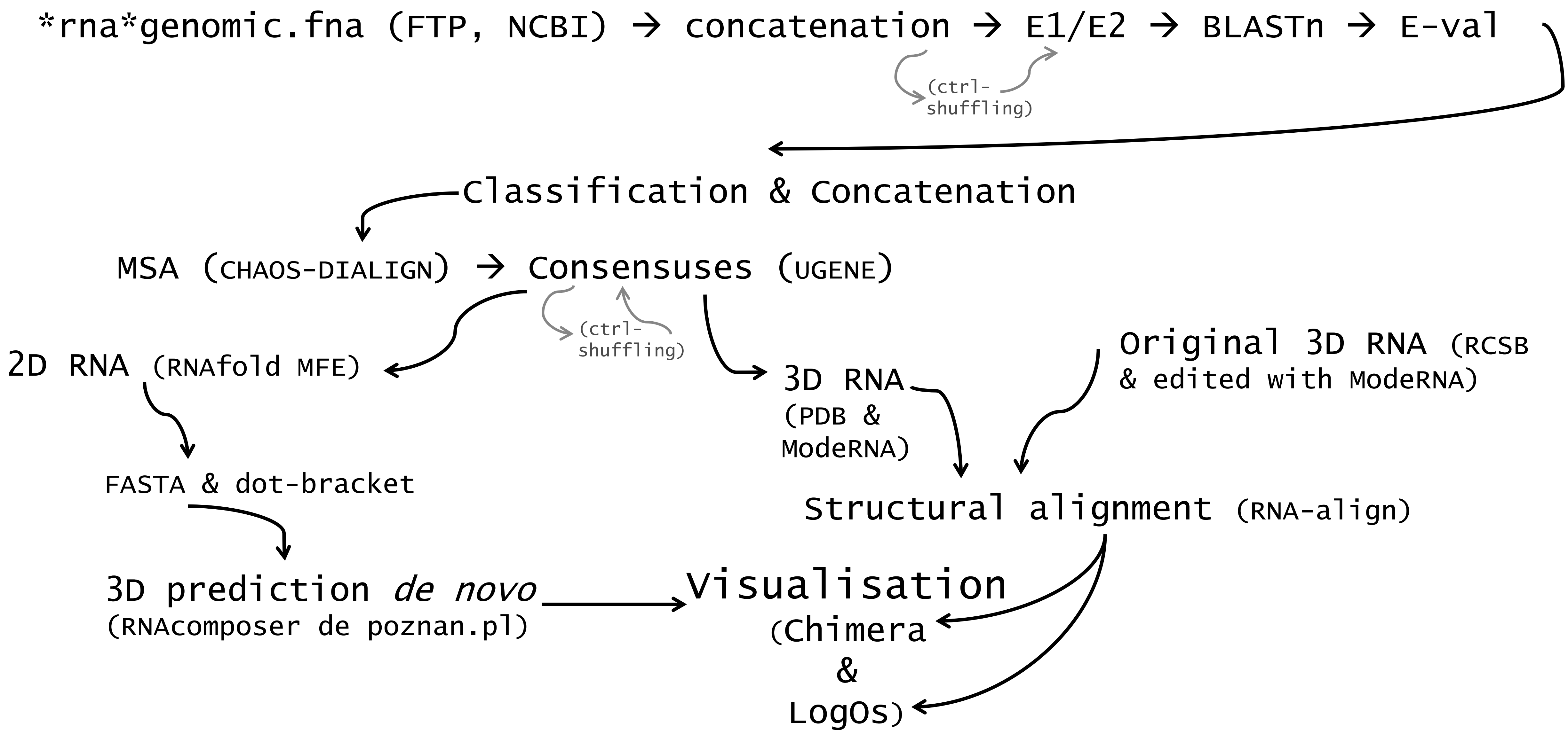

Supplement: Supplementary file 1 [file genes-15-01195-s001.zip › S1. Flowchart_RNAsbeforeLUCA_aug2024.pdf]
